# A Network Pharmacology Review of Plant-Derived Anticancer Compounds in Lung, Breast, Colorectal and Prostate Cancer

**DOI:** 10.3390/ijms27146177

**Published:** 2026-07-10

**Authors:** Anna Merecz-Sadowska, Arkadiusz Sadowski, Karolina Zajdel, Aneta Jęcek, Przemysław Sitarek, Radosław Zajdel

**Affiliations:** 1 Department of Economic and Medical Informatics, University of Lodz, 90-214 Lodz, Poland; arkadiusz.sadowski@uni.lodz.pl (A.S.); radoslaw.zajdel@uni.lodz.pl (R.Z.); 2 Department of Public Health, Medical University of Lodz, 90-752 Lodz, Poland; karolina.smigiel@umed.lodz.pl (K.Z.); aneta.jecek@umed.lodz.pl (A.J.); 3 Department of Medical Biology, Medical University of Lodz, Muszynskiego 1, 90-151 Lodz, Poland; przemyslaw.sitarek@umed.lodz.pl

**Keywords:** network pharmacology, plant-derived compounds, anticancer, lung cancer, breast cancer, colorectal cancer, prostate cancer, PI3K/AKT/mTOR, multitarget drug discovery, in silico validation

## Abstract

Lung, breast, colorectal and prostate cancer account for over 41% of global cancer incidence and 39% of mortality, yet durable control of advanced disease remains limited. Plant secondary metabolites are promising multitarget leads, but their polypharmacological mechanisms cannot be captured by single-target approaches, and the evidence across these four cancers has not been synthesised within a unified framework. This review provides an integrated comparative analysis of network-pharmacology studies of plant-derived anticancer compounds across the four cancers, cataloguing phytochemical profiles, identifying shared and cancer-specific targets, quantifying the concordance between computational predictions and experimental validation, and appraising the translational gap. A systematic search of biomedical databases (2016–2026) identified 101 peer-reviewed studies (40 breast, 33 colorectal, 24 lung, and 14 prostate) combining network pharmacology with experimental validation. AKT1, EGFR, TP53, STAT3, MAPK1/3, CASP3, and HSP90AA1 recurred as cross-cancer hub genes, with the phosphoinositide 3-kinase/AKT and mitogen-activated protein kinase pathways most frequently implicated. Cancer-specific signatures comprised the androgen receptor in prostate, the oestrogen receptor and human epidermal growth factor receptor 2 in breast, β-catenin/Wnt in colorectal, and the epidermal growth factor receptor/RAS axis with epithelial-to-mesenchymal transition effectors in lung cancer. Flavonoids, terpenoids, alkaloids, and polyphenols predominated. The persistent validation gap remains the principal barrier to translation.

## 1. Introduction

Cancer remains one of the leading causes of morbidity and mortality worldwide. According to the GLOBOCAN 2022 [1] estimates published by the International Agency for Research on Cancer, approximately 19.97 million new cancer cases and 9.74 million cancer-related deaths occurred globally across 185 countries in that year. Among the more than thirty cancer sites catalogued, four malignancies, namely lung, breast, colorectal, and prostate cancer, jointly accounted for over 8.17 million new cases (approximately 41% of global incidence) and an estimated 3.78 million deaths (approximately 39% of cancer mortality) in 2022. Lung cancer was both the most frequently diagnosed malignancy and the leading cause of cancer death, with 2,480,675 new cases and 1,817,469 deaths; it was followed by female breast cancer (2,296,840 cases; 666,103 deaths), colorectal cancer (1,926,425 cases; 904,019 deaths) and prostate cancer (1,467,854 cases; 397,430 deaths) [1]. These figures establish the four cancers examined in this review as priorities not only for primary prevention but also for the development of more effective and better tolerated systemic therapies.

Despite considerable progress in surgical resection, radiotherapy, cytotoxic chemotherapy, molecularly targeted small-molecule inhibitors and immune-checkpoint blockade, durable disease control is not achieved in a substantial proportion of patients with advanced or recurrent disease. Common obstacles include intrinsic and acquired drug resistance, dose-limiting toxicities, narrow therapeutic indices, and the genetic, epigenetic, and microenvironmental heterogeneity of tumours that allows clonal escape from single-target interventions. In each of the four malignancies considered here, resistance to first-line systemic treatment, such as tyrosine-kinase inhibitor resistance in non-small-cell lung cancer, endocrine resistance in luminal breast cancer, castration-resistant progression in prostate cancer, and failure of 5-fluorouracil and oxaliplatin in colorectal cancer, is an established cause of treatment failure and motivates the continued search for chemically diverse, multitarget therapeutic leads [2].

Plants have provided some of the most clinically successful anticancer drugs used in modern oncology. Notable examples include: the vinca alkaloids vinblastine and vincristine, isolated from *Catharanthus roseus*; the taxanes paclitaxel and docetaxel, originally derived from *Taxus brevifolia*; the podophyllotoxin-based topoisomerase II inhibitors etoposide and teniposide; and the camptothecin analogues topotecan and irinotecan, obtained from *Camptotheca acuminata* [3,4]. In their analysis of new chemical entities approved between 1981 and 2019, Newman and Cragg reported that approximately 65% of all small-molecule anticancer drugs are either natural products, direct semisynthetic derivatives of natural products, or natural-product mimetics, demonstrating that plants and other natural sources remain a major source of leads for oncological drug discovery despite advances in synthetic medicinal chemistry [5]. Beyond purified single compounds, complex plant preparations and their secondary metabolites, particularly alkaloids, flavonoids, terpenoids and polyphenols, exert pleiotropic effects on multiple cancer hallmarks, including proliferation, apoptosis, angiogenesis, invasion, metabolic reprogramming, and immune evasion [6,7].

The multitarget activity of plant-derived compounds, which accounts for much of their therapeutic potential, also poses a methodological challenge, because linking a chemically complex extract to defined molecular targets and signalling pathways cannot be achieved with conventional single-target approaches. To address this challenge, Hopkins formalised the concept of network pharmacology in 2008 as a new paradigm in drug discovery that treats biological systems as molecular networks and exploits the polypharmacology of small molecules to modulate disease-relevant hubs and modules rather than isolated proteins [8]. In the subsequent two decades, network pharmacology has been widely adopted across drug discovery and natural-product research, including the study of plant-derived therapies, with approximately 9000 network-pharmacology publications indexed in PubMed in 2024 alone; over 40% of these concerned traditional Chinese medicine (TCM) theory, prescriptions, or single herbs [9]. The standard workflow typically integrates compound identification from plant extracts, in silico prediction of putative targets using cheminformatic and bioinformatic databases such as the Traditional Chinese Medicine Systems Pharmacology database (TCMSP), SwissTargetPrediction, GeneCards and OMIM, construction of compound-target and protein–protein interaction networks in STRING and Cytoscape, functional enrichment of biological pathways through the Kyoto Encyclopedia of Genes and Genomes (KEGG) and Gene Ontology (GO), and molecular docking or dynamics simulations to assess binding plausibility [10].

This integrative workflow is particularly well suited to plant-based interventions because it accommodates multicomponent, multitarget mechanisms and generates testable hypotheses at relatively modest experimental cost. Despite the rapid expansion of network-pharmacology research, the published literature remains fragmented across single-cancer reports and methodological papers that are not specific to any particular cancer, and no synthesis has so far placed the four globally leading cancers within a single methodological framework to map the molecular and chemical commonalities and the cancer-specific signatures that emerge from the cumulative evidence base.

The present review provides an integrated comparative analysis of network-pharmacology applications to plant-derived anticancer compounds across lung, breast, colorectal and prostate cancer, which together account for over 41% of global cancer incidence and 39% of cancer mortality. We systematically examine 101 peer-reviewed studies retrieved through a Preferred Reporting Items for Systematic Reviews and Meta-Analyses (PRISMA)-compliant search of four biomedical databases, with the explicit objectives of: cataloguing the plant species, extracts and individual phytochemicals most frequently investigated against the four selected cancers; identifying the cross-cancer hub genes and enriched signalling pathways that recur as polypharmacological anchors; delineating cancer-specific molecular signatures that complement the shared targets; quantifying the concordance between in silico predictions and in vitro or in vivo experimental validation, with particular emphasis on the persistent in vivo validation gap; and appraising the translational potential of the identified leads together with the methodological limitations that currently constrain the field. By integrating these five dimensions, this review highlights both shared molecular vulnerabilities, such as the phosphoinositide 3-kinase/AKT/mechanistic target of rapamycin (PI3K/AKT/mTOR), mitogen-activated protein kinase (MAPK), p53, nuclear factor κB (NF-κB) and Wnt/β-catenin axes, and tumour-specific targets, and outlines how network pharmacology can serve as a quantitative link between phytochemistry and clinical oncology, while offering practical recommendations to accelerate the transition of plant-derived multitarget candidates from in silico hypotheses to clinically validated agents.

## 2. Review Methodology

**Figure 1:**
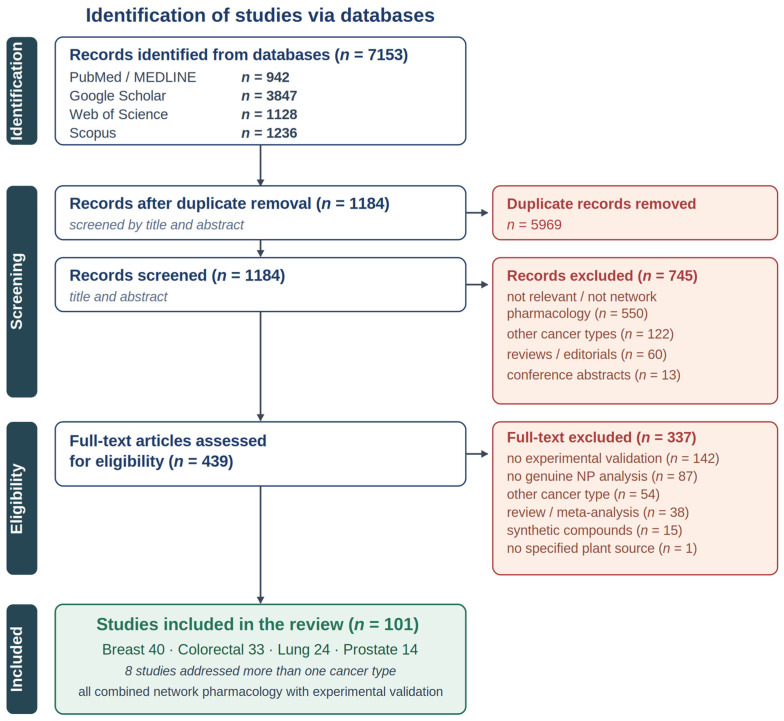
PRISMA flow diagram of the literature search and selection process.

A targeted literature search was conducted in four major biomedical databases, namely PubMed/MEDLINE, Google Scholar, Web of Science and Scopus, covering articles published between January 2016 and May 2026. The search combined the core search term “network pharmacology” with the terms “plant”, “phytochemical”, “natural product”, “herbal extract” or “traditional Chinese medicine”, and with at least one of four cancer descriptors: “lung cancer” (including “non-small-cell lung cancer” and “lung adenocarcinoma”), “breast cancer” (including “triple-negative breast cancer”), “colorectal cancer” (including “colon cancer”) or “prostate cancer”. Boolean operators (AND, OR) and truncation symbols were used to broaden the search. The complete search and selection process is summarised as a PRISMA flow diagram in Figure 1.

Studies were considered eligible if they reported a network-pharmacology analysis of a plant extract, fraction or polyherbal formulation, or an individual phytochemical isolated from a specified plant material; included experimental validation of the predicted targets or pathways, either in vitro or in vivo; and addressed at least one of the four selected cancers. Peer-reviewed original research articles indexed in at least one of the four databases were retained. Studies were excluded if they consisted of purely in silico analyses without experimental validation; were review articles, meta-analyses, conference abstracts or opinion pieces; focused on synthetic small molecules not of plant origin; or addressed tumour types other than lung, breast, colorectal or prostate cancer.

The initial search returned 7153 records across the four databases (PubMed, *n* = 942; Google Scholar, *n* = 3847; Web of Science, *n* = 1128; Scopus, *n* = 1236). After removal of 5969 duplicates, 1184 unique records were screened by title and abstract, of which 745 were excluded (550 were not relevant to the review topic or were not network-pharmacology studies, 122 addressed cancer types other than the four selected cancers, 60 wee review articles or editorials, and 13 were conference abstracts). The remaining 439 full-text articles were assessed for eligibility, and 337 were excluded for the following reasons: absence of experimental validation (*n* = 142); absence of a genuine network-pharmacology analysis (*n* = 87); focus on a cancer type other than the four selected cancers (*n* = 54); review or meta-analysis (*n* = 38); focus on synthetic compounds not of plant origin (*n* = 15); and focus on an individual phytochemical not isolated from a specified plant source (*n* = 1). After this multi-step selection, 101 reports fulfilled all inclusion criteria. The corpus comprised 40 articles on breast cancer, 33 on colorectal cancer, 24 on lung cancer and 14 on prostate cancer; eight studies addressed more than one cancer type and were therefore counted in each relevant category (two of them in three categories), which accounts for the difference between the total number of unique studies (101) and the sum of the cancer-specific groups (111). 

For each included study, we recorded the plant source (species or formula, plant part, and preparation), the representative extract or compound investigated, the major chemical classes of the bioactive constituents, the in silico methodology used (compound libraries, target-prediction tools, network-construction software and pathway-enrichment databases), the top five predicted hub genes, the enriched signalling pathways, the in vitro and in vivo experimental models used for validation, any clinical relevance reported by the original authors, and the source reference. These data are summarised, organised by cancer type, in dedicated Supplementary Materials (Table S1 for lung cancer, Table S2 for breast, Table S3 for colorectal, and Table S4 for prostate).

Two limitations of this search strategy should be acknowledged. First, although no explicit language restriction was applied, the search terms were formulated in English, and all of the studies that ultimately met the inclusion criteria were published in English; relevant network-pharmacology studies reported in other languages, in particular the substantial Chinese-language literature on traditional Chinese medicine, may therefore have been under-represented or missed, introducing a potential language bias. Second, because studies reporting positive computational and experimental findings are more likely to be published than those with negative or inconclusive results, a degree of publication bias cannot be excluded. The present synthesis should accordingly be interpreted as a structured map of the reported evidence rather than as an unbiased estimate of the anticancer efficacy of plant-derived compounds.

## 3. Theoretical Framework

### 3.1. Anticancer Mechanisms of Plant Secondary Metabolites

**Figure 2:**
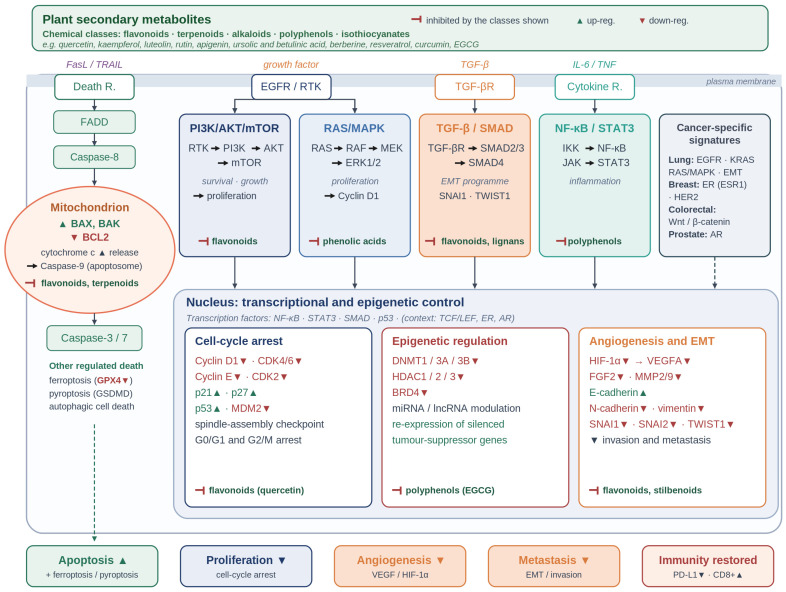
Signalling pathways and molecular mechanisms through which plant secondary metabolites exert anticancer activity. Membrane receptors (death receptors, EGFR/receptor tyrosine kinases, the TGF-β receptor and cytokine receptors) activate the shared cascades that recur across the reviewed network-pharmacology studies (PI3K/AKT/mTOR, RAS/MAPK, TGF-β/SMAD and NF-κB/STAT3), while the intrinsic (mitochondrial) and extrinsic (death-receptor) apoptotic pathways, together with ferroptosis, pyroptosis and autophagic cell death, drive regulated cell death. These cascades converge on the nucleus, where plant metabolites promote cell-cycle arrest, epigenetic re-activation of silenced tumour-suppressor genes, and suppression of angiogenesis and the epithelial-to-mesenchymal transition. For each node, the chemical classes most frequently implicated are indicated (a blunt-ended symbol denotes inhibition by the compound classes; ▲ denotes upregulation and ▼ denotes downregulation; the colours of the symbols carry no additional meaning). Solid arrows indicate direct signalling and dashed arrows indicate indirect or multi-step effects. Cancer-type-specific signatures (EGFR/KRAS in lung, ER/HER2 in breast, Wnt/β-catenin in colorectal, and the androgen receptor in prostate cancer) act in addition to the shared core. The net effect is increased apoptosis and reduced proliferation, angiogenesis, metastasis, and immune evasion. Abbreviations: AKT, AKT serine/threonine kinase; AR, androgen receptor; BAK, BCL2 antagonist/killer; BAX, BCL2-associated X protein; BCL2, B-cell lymphoma 2; BRD4, bromodomain-containing protein 4; CD8, cluster of differentiation 8; CDK, cyclin-dependent kinase; DNMT, DNA methyltransferase; EGCG, epigallocatechin-3-gallate; EGFR, epidermal growth factor receptor; EMT, epithelial-to-mesenchymal transition; ER, oestrogen receptor; ERK, extracellular signal-regulated kinase; ESR1, oestrogen receptor 1; FADD, Fas-associated death domain protein; FasL, Fas ligand; FGF2, fibroblast growth factor 2; GPX4, glutathione peroxidase 4; GSDMD, gasdermin D; HDAC, histone deacetylase; HER2, human epidermal growth factor receptor 2; HIF-1α, hypoxia-inducible factor 1α; IKK, IκB kinase; JAK, Janus kinase; KRAS, Kirsten rat sarcoma viral oncogene homologue; lncRNA, long non-coding RNA; MAPK, mitogen-activated protein kinase; MDM2, mouse double minute 2 homologue; MEK, MAPK/ERK; miRNA, microRNA; MMP, matrix metalloproteinase; mTOR, mechanistic target of rapamycin; NF-κB, nuclear factor κB; PD-L1, programmed death-ligand 1; PI3K, phosphoinositide 3-kinase; RAF, rapidly accelerated fibrosarcoma; RAS, rat sarcoma; RTK, receptor tyrosine kinase; SMAD, mothers against decapentaplegic homologue; SNAI1, snail family transcriptional repressor 1; SNAI2, snail family transcriptional repressor 2; STAT3, signal transducer and activator of transcription 3; TCF/LEF, T-cell factor/lymphoid enhancer factor; TGF-β, transforming growth factor β; TRAIL, TNF-related apoptosis-inducing ligand; TWIST1, twist family bHLH transcription factor 1; VEGF, vascular endothelial growth factor; VEGFA, vascular endothelial growth factor A.

Plant-derived compounds exert their antitumour activity through several interconnected mechanisms, which are summarised schematically in Figure 2. Plant secondary metabolites induce programmed cell death in cancer cells through both the extrinsic (death-receptor) and the intrinsic (mitochondrial) apoptotic pathways. Many flavonoids, alkaloids and terpenoids upregulate pro-apoptotic Bcl-2 family proteins (BAX, BAK, and BIM) and downregulate antiapoptotic members (BCL2, BCL-XL, and MCL1), trigger mitochondrial outer membrane permeabilisation and cytochrome c release, and activate the caspase cascade (caspase-9, followed by effector caspase-3 and caspase-7), leading to cleavage of poly(ADP-ribose) polymerase (PARP) and DNA fragmentation [11,12]. In parallel, several phytochemicals engage death receptors such as Fas and the tumour necrosis factor (TNF)-related apoptosis-inducing ligand receptors TRAIL-R1 and TRAIL-R2 to recruit Fas-associated death domain protein (FADD) and procaspase-8, activating the extrinsic apoptotic pathway, and act simultaneously on both pathways through tBid-mediated crosstalk [13]. Additional forms of regulated cell death, including ferroptosis (GPX4 inhibition and lipid peroxidation), pyroptosis (gasdermin cleavage) and autophagic cell death, have been reported for selected polyphenols and terpenoids [14].

Plant-derived compounds frequently inhibit cancer cell proliferation by inducing cell-cycle arrest at the G0/G1, S or G2/M checkpoints through modulation of cyclin and cyclin-dependent kinase (CDK) complexes together with their endogenous inhibitors. Polyphenols such as resveratrol and quercetin, and flavonols including kaempferol, downregulate cyclin D1, cyclin E and CDK2, CDK4 and CDK6, while concurrently upregulating p21WAF1/CIP1 and p27KIP1, leading to G0/G1 arrest [15,16]. Alkaloids and taxane-type terpenoids interfere with microtubule dynamics, either by stabilisation (taxanes) or by polymerisation inhibition (vinca alkaloids), and, consequently, arrest cells at the G2/M boundary by activating the spindle-assembly checkpoint [17]. Reactivation of p53, inhibition of MDM2 and suppression of the PI3K/AKT/mTOR proliferation axis are additional mechanisms commonly reported across in vitro models [18].

Tumour angiogenesis depends on the balance between pro-angiogenic factors (VEGFA, FGF2, PDGFB, MMP2 and MMP9) and endogenous inhibitors (THBS1, angiostatin). Plant secondary metabolites including flavonoids, stilbenoids and lignans suppress VEGFA expression, hypoxia-inducible factor 1α (HIF-1α) stabilisation and endothelial tube formation, and they inhibit matrix metalloproteinases involved in extracellular matrix degradation [19,20]. The same compounds frequently impair the epithelial-to-mesenchymal transition (EMT), restoring E-cadherin and downregulating N-cadherin, vimentin and the SNAI1, SNAI2 and TWIST1 transcription factors that drive metastatic dissemination [21]. Many of these effects map onto axes that include AKT and glycogen synthase kinase 3β (GSK3β), MAPK and transforming growth factor β (TGF-β)/SMAD, which recur as hub modules in the reviewed network-pharmacology studies.

The tumour microenvironment (TME) comprises stromal cells, immune cells, extracellular matrix and a complex cytokine network that supports tumour growth and treatment resistance. Several plant metabolites remodel the TME by polarising tumour-associated macrophages from a tumour-promoting M2 phenotype towards a tumouricidal M1 phenotype by reducing infiltration of myeloid-derived suppressor cells and regulatory T cells, and by enhancing cytotoxic CD8+ T-cell activity [22,23]. Direct effects on immune checkpoints, including downregulation of programmed death-ligand 1 (PD-L1) in tumour cells, have been reported for polyphenols such as curcumin and resveratrol, supporting potential combination strategies with immune-checkpoint inhibitors [24]. Anti-inflammatory actions mediated by suppression of NF-κB, signal transducer and activator of transcription 3 (STAT3) and cyclooxygenase-2 (COX-2) reinforce these immunomodulatory effects and converge on pathways frequently identified in pathway-enrichment analyses across the reviewed studies.

Plant secondary metabolites also act on the cancer epigenome by inhibiting DNA methyltransferases (DNMT1, DNMT3A and DNMT3B), histone deacetylases (HDAC1, HDAC2 and HDAC3) and the bromodomain reader BRD4, and by altering microRNA expression profiles. Dietary polyphenols such as epigallocatechin-3-gallate (EGCG), resveratrol and curcumin restore the expression of silenced tumour-suppressor genes through DNA hypomethylation and increased histone acetylation [25,26]. Long non-coding RNAs and microRNAs that regulate hub genes recurrently identified in network-pharmacology studies (TP53, EGFR, and BCL2) are increasingly recognised as downstream effectors of phytochemical action, providing a molecular link between dietary or pharmacological exposure to plant compounds and changes in tumour cell phenotype.

### 3.2. Network-Pharmacology Workflow in Cancer Research

**Figure 3:**
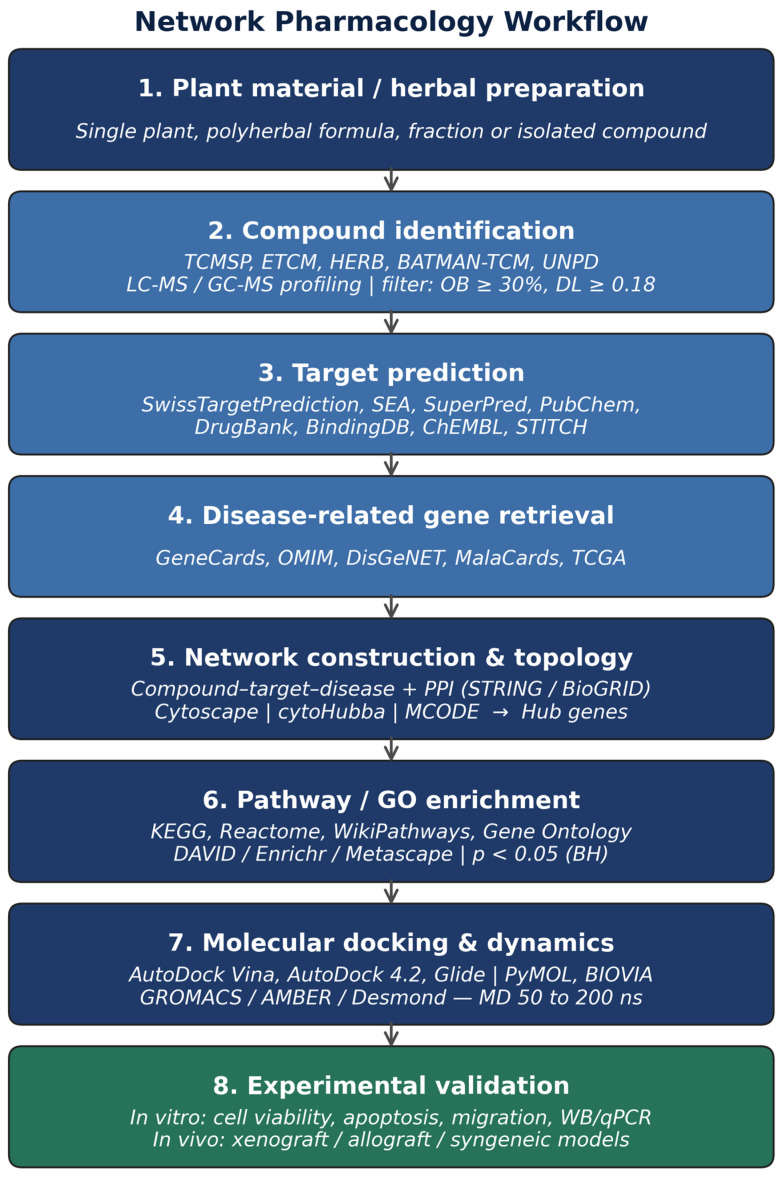
Schematic workflow of network pharmacology applied to plant-derived anticancer compounds. The pipeline begins with chemical characterisation of the plant material, proceeds through in silico target prediction and network analysis, and culminates in experimental validation in vitro and in vivo.

The first step of any network-pharmacology analysis (Figure 3) is the systematic identification of the bioactive constituents of the plant material under study. For traditional Chinese medicine and other well-characterised herbal materials, TCMSP (https://tcmsp-e.com, accessed on 10 April 2026) provides curated information on chemical structures, oral bioavailability (OB), drug-likeness (DL), Caco-2 permeability and predicted targets for more than 30,000 ingredients from 499 herbs [27]. Complementary resources include the Encyclopaedia of Traditional Chinese Medicine (ETCM), the BATMAN-TCM platform, HERB, the Universal Natural Products Database (UNPD) and SymMap, while phytochemical profiles obtained experimentally by liquid chromatography–mass spectrometry (LC-MS) or gas chromatography–mass spectrometry (GC-MS) are routinely used to confirm the actual constituents of the extract under investigation [10]. Default screening criteria of OB ≥ 30% and DL ≥ 0.18 are widely applied, although these thresholds are increasingly relaxed when sufficient chromatographic evidence is available.

Once the chemical composition of the plant material is defined, putative protein targets for each compound are predicted using three complementary strategies: ligand-similarity methods that exploit chemical resemblance to drugs of known activity, implemented in SwissTargetPrediction, SuperPred and the Similarity Ensemble Approach (SEA); docking-based reverse screening against panels of cancer-relevant protein structures retrieved from the Protein Data Bank; and text-mining and curated database lookup in PubChem, DrugBank, BindingDB, ChEMBL, the Therapeutic Target Database (TTD) and STITCH [28,29]. Predicted targets are then intersected with disease-associated gene sets compiled from GeneCards, OMIM, DisGeNET, MalaCards and The Cancer Genome Atlas (TCGA) for the cancer of interest, with the overlap defining the shared compound and disease target list that is carried forward to network analysis. Hub-gene candidates emerging from this intersection are routinely confirmed by Western blot, qPCR or immunohistochemistry in subsequent in vitro experiments.

The intersected compound and target sets are imported into Cytoscape to build compound-target bipartite networks and, after integration with protein–protein interaction (PPI) data from STRING, BioGRID or HuRI, expanded into target–target PPI networks [30,31]. Hub genes are identified by topological measures such as degree, betweenness centrality, closeness centrality and Maximal Clique Centrality (MCC), commonly calculated with the cytoHubba plugin. Module-detection algorithms (MCODE, ClusterONE) further partition the network into densely connected functional subunits, while plugins such as NetworkAnalyzer and CentiScaPe support comparison across studies. In the reviewed corpus, networks typically contained between 50 and 300 nodes and 100 to 1500 edges, and the top five to 20 hub genes were carried forward to pathway enrichment.

Hub genes and entire target lists are subjected to functional enrichment analysis to identify the biological processes, molecular functions and cellular components affected (Gene Ontology, GO) and the signalling pathways involved (KEGG, Reactome, WikiPathways) [32,33]. The DAVID, Enrichr, Metascape and ClusterProfiler tools are most often used in the reviewed studies, with statistical thresholds typically set at *p* < 0.05 (Benjamini–Hochberg correction) and minimum gene counts of three to five per category. Across cancer types, pathway enrichment recurrently identifies “Pathways in cancer”, “PI3K-Akt signaling pathway”, “MAPK signaling pathway”, “p53 signaling pathway”, “Apoptosis”, “Cell cycle” and tumour-specific KEGG entries (e.g., “Non-small cell lung cancer”, “Prostate cancer”, “Colorectal cancer”, and “Breast cancer”), suggesting that plant compounds preferentially engage a relatively conserved set of oncogenic modules.

Following the identification of priority hub targets, molecular docking simulations are used to assess whether the most abundant plant compounds can bind the predicted proteins with thermodynamically plausible affinities. AutoDock Vina, AutoDock and Schrödinger Glide are the most widely used docking engines among the reviewed studies, with PyMOL, Discovery Studio Visualizer and BIOVIA used for visualisation of binding modes and intermolecular contacts [34]. Binding energies more negative than −5.0 to −7.0 kcal/mol are commonly considered indicative of favourable interaction, although thresholds vary across studies. To assess complex stability over time, molecular-dynamics simulations (typically 50 to 200 ns trajectories in GROMACS, AMBER or Desmond) are increasingly applied, with root mean square deviation (RMSD), root mean square fluctuation (RMSF), radius of gyration, solvent-accessible surface area (SASA) and hydrogen-bond analyses used to quantify the conformational behaviour of the protein and ligand. These in silico predictions are subsequently tested experimentally, both in vitro and in vivo.

## 4. Network Pharmacology in the Study of Specific Cancer Types

### 4.1. Lung Cancer

Lung cancer remains the most frequently diagnosed and most lethal malignancy worldwide [1]. Approximately 85% of cases are of non-small-cell lung cancer (NSCLC) histology, with lung adenocarcinoma (LUAD) and lung squamous-cell carcinoma (LUSC) representing the two dominant subtypes, while small-cell lung cancer (SCLC) accounts for the remaining 15% [35]. The molecular heterogeneity of NSCLC is profound. Lung adenocarcinomas frequently carry activating mutations in EGFR, KRAS or BRAF, gene fusions involving ALK, ROS1, RET or NTRK, and copy-number alterations in MET, while squamous-cell carcinomas show frequent disruption of the PI3K, FGFR and NRF2 pathways. The clinical management of NSCLC has evolved considerably over the past two decades, with the routine implementation of EGFR, ALK, ROS1 and BRAF tyrosine-kinase inhibitors in molecularly selected patients and the broad adoption of immune-checkpoint blockade targeting the programmed death-1 (PD-1) and PD-L1 axis in both early-stage and metastatic disease. Despite these advances, five-year survival of metastatic NSCLC remains below 10%, in large part because acquired resistance mechanisms emerge almost inevitably during treatment. The best-characterised mechanisms include secondary EGFR mutations such as T790M and C797S, activation of bypass pathways through MET or HER2 amplification, transformation to small-cell or neuroendocrine phenotypes, and the emergence of phenotypically plastic, epithelial-to-mesenchymal-like tumour cell states that confer multidrug resistance [36]. These persistent challenges have motivated an intensive search for chemically complex, multitarget plant-derived agents capable of simultaneously engaging several oncogenic nodes within a single compound mixture, an approach that aligns with the polypharmacology principle underlying network pharmacology.

The investigations analysed in this section evaluated a remarkably diverse set of plant materials, drawn from several different geographical regions. The first and larger cluster comprised plant materials investigated as crude extracts, solvent fractions or chemically characterised preparations, including the rhizomatous *Stemona tuberosa* [37], the bulb-forming *Anemarrhena asphodeloides* [38], the milkvetch *Astragalus mongholicus* [39], the liquorice *Glycyrrhiza glabra* [40], the honeysuckle *Lonicera japonica* [41], the ginsengs *Panax ginseng* [42] and *Panax quinquefolius* [43], the tuberous *Pinellia ternata* [44], the yam *Dioscorea zingiberensis* [45], the herbaceous *Scleromitrion diffusum* [46], a clinically used eighteen-herb Chinese Herbal Medicine formula associated with a reported survival benefit in stage IV lung adenocarcinoma [47], the lemon balm *Melissa officinalis* [48], the South American macela *Achyrocline satureioides* [49], the ornamental *Nandina domestica* [50], the leafy climber *Cissus trifoliata* [51], the dye plant *Coreopsis tinctoria* [52], the Indian borage *Coleus amboinicus* [53], the silverberry *Elaeagnus caudata* [54], the fish mint *Houttuynia cordata* [55], the common dandelion *Taraxacum officinale* [56], and the Egyptian Amaryllidaceae bulbs of *Crinum bulbispermum*, *Pancratium maritimum* and *Hippeastrum vittatum* together with the flowering aerial parts of Centaurea scoparia [57]. A second cluster comprised plants whose principal bioactive material is a complex volatile essential oil characterised by GC-MS profiling, exemplified by the Indian bay leaf *Cinnamomum tamala* [58], the perennial grass *Chrysopogon zizanioides* (vetiver) [59] and the niaouli tree *Melaleuca quinquenervia* [60]. The dominant chemical classes investigated against NSCLC across these studies were flavonoids, most prominently quercetin, kaempferol, luteolin, apigenin, baicalein and epigallocatechin-3-gallate, together with phytosterols such as β-sitosterol and stigmasterol, a wide range of triterpenoids and triterpene saponins that included ursolic acid, taraxasterol, 18α-glycyrrhetinic acid, the steroidal saponin dioscin and the ginsenosides Rb3, Rc, Rh2 and F2, the Amaryllidaceae-type alkaloids crinamine, ismine, lycorine and hemanthidine, and the sesquiterpenoid-rich essential oils mentioned above.

Across this body of work, the network-pharmacology analyses converge on a highly conserved set of NSCLC hub genes. The serine and threonine protein kinase AKT1 emerges as the single most frequently identified target, followed in approximate order of recurrence by the epidermal growth factor receptor EGFR, the tumour suppressor TP53, the mitogen-activated protein kinases MAPK1 and MAPK3 (also known as ERK2 and ERK1), the apoptosis effector caspase-3, the transcription factor STAT3, the molecular chaperone HSP90AA1 and the regulatory and catalytic subunits of phosphoinositide 3-kinase, PIK3R1 and PIK3CA. Additional nodes that recur in several studies include the non-receptor tyrosine-kinase SRC, the antiapoptotic regulator BCL2, the mechanistic target of rapamycin mTOR, the matrix metalloproteinase MMP9 and the activator protein 1 (AP-1) component JUN. Pathway-enrichment analyses are dominated by the PI3K-Akt signalling pathway, which is identified more often than any other functional module, followed by the MAPK signalling pathway, the lung-cancer-specific KEGG entry “Non-small cell lung cancer”, the pathway annotated as “EGFR tyrosine-kinase inhibitor resistance”, the broad term “Pathways in cancer”, the apoptosis pathway, and the inflammation-linked TNF and interleukin-17 (IL-17) signalling pathways. Taken together, this pattern indicates that plant-derived NSCLC modulators preferentially engage the canonical EGFR/PI3K/AKT/mTOR and EGFR/RAS/MAPK signalling axes that are also targeted by clinically approved tyrosine-kinase inhibitors, but they do so in a polypharmacological fashion that can simultaneously affect downstream regulators of apoptosis through BCL2, BAX, caspase-3 and p53, of tumour inflammation through TNF and IL-17, and of metastatic dissemination through MMP9 and the principal transcription factors that drive epithelial-to-mesenchymal transition.

Experimental validation in this corpus was, almost without exception, anchored in the human lung adenocarcinoma cell line A549, which has become the de facto reference model for NSCLC research and was used in nearly every investigation discussed here. To establish selectivity, A549 was commonly paired with the non-tumourigenic bronchial epithelial line BEAS-2B or with the murine fibroblast line L929, and occasionally with the simian kidney line CV-1, with normal human embryonic kidney HEK-293 cells or with neonatal human dermal fibroblasts. A subset of studies extended the analysis to other NSCLC-derived cell lines, notably the EGFR-wild-type lines H1299 and NCI-H460, the EGFR-mutant line HCC827 and the LUSC-derived line NCI-H1395. The standard experimental toolkit was likewise consistent across studies. Cell viability was measured by the MTT or CCK-8 colorimetric assays, clonogenic survival by colony-formation assays, cell migration and invasion by wound-healing scratch assays and transwell chambers, apoptosis induction by Annexin V and propidium iodide dual staining or by terminal deoxynucleotidyl transferase dUTP nick-end labelling (TUNEL), and cell-cycle distribution by flow cytometry. At the molecular level, modulation of the predicted hub genes was confirmed either by Western blot quantification of total and phosphorylated forms of AKT, ERK1/2, EGFR and STAT3 or by quantitative reverse-transcription PCR (qRT-PCR) of the corresponding mRNAs. In vivo validation among the network-pharmacology studies reviewed here, however, remained scarce: only a minority of investigations advanced beyond cell culture, with the two animal studies in this corpus both using A549 xenografts implanted in immunodeficient mice, namely an investigation of total flavonoids from *Coreopsis tinctoria* [52] and an investigation of quercetin obtained from *Achyrocline satureioides* [49]. This consistent under-representation of in vivo evidence is one of the most striking limitations of the lung cancer network-pharmacology literature reviewed here.

Several investigations within this corpus warrant individual discussion because of the depth and originality of their integrated validation. The methanolic extract of *Anemarrhena asphodeloides* [38] was screened through TCMSP, yielding fifteen bioactive constituents that mapped to several hundred NSCLC-related genes, with kaempferol, asperglaucide and coumaroyltyramine emerging as the most active compounds; their predicted binding to AKT1, SRC and HSP90AA1 was confirmed in 100-nanosecond molecular-dynamics simulations and, in parallel, in transwell migration assays, in TUNEL-positive apoptosis quantification and in qRT-PCR validation of the corresponding hub-gene transcripts in A549 cells, with sparing of BEAS-2B controls. The methanolic extract of *Achyrocline satureioides* [49] combined four distinct experimental layers within a single study: high-resolution ultra-high-performance liquid chromatography tandem mass spectrometry (UHPLC-MS/MS) for phytochemical characterisation, network-pharmacology target prediction, intracellular metabolomic profiling, and an A549 xenograft model in nude mice; subsequent matrix-assisted laser desorption/ionisation (MALDI) mass-spectrometry imaging of the resulting tumour tissue identified more than fifty differentially abundant metabolites and revealed a pronounced disruption of the tricarboxylic acid cycle in vivo. The compound amorphigenin, isolated from the methanolic leaf extract of *Elaeagnus caudata* [54], displayed an HSP90AA1 docking energy more favourable than that of the reference inhibitor geldanamycin and was selectively cytotoxic to A549 cells while sparing normal L929 fibroblasts. Other particularly notable contributions came from the essential oil of *Cinnamomum tamala* [58], which induced caspase-dependent apoptosis driven by reactive oxygen species (ROS) through coordinated engagement of TP53, JUN, MAPK3 and HIF1A, and from the steroidal saponin dioscin obtained from *Dioscorea zingiberensis* [45], which reversed the epithelial-to-mesenchymal transition phenotype in lung adenocarcinoma cells through inactivation of the AKT/GSK3β/mTOR signalling axis. The vetiver essential oil of *Chrysopogon zizanioides* [59] and the niaouli oil of *Melaleuca quinquenervia* [60] further illustrate that chemically complex mixtures of sesquiterpenoids and monoterpenoids can recapitulate, in vitro, the multitarget engagement that is otherwise difficult to achieve with single small molecules, and that this multitarget pattern is captured by the network-pharmacology workflow.

Full details of the lung cancer studies analysed in this review, including plant source, representative compounds, hub genes, enriched pathways and experimental models, are provided in Table S1.

### 4.2. Breast Cancer

Breast cancer is the second most frequently diagnosed malignancy worldwide and the leading cancer in women [1]. The disease is biologically heterogeneous and is routinely subclassified by immunohistochemistry into four major intrinsic subtypes, namely Luminal A, Luminal B, HER2-enriched and triple-negative breast cancer, which are defined by the expression of the oestrogen receptor (encoded by ESR1), the progesterone receptor and the human epidermal growth factor receptor 2 encoded by ERBB2, together with the proliferation marker Ki-67 [61]. The molecular complexity extends well beyond this immunohistochemical scheme. Large-scale genomic profiling has identified recurrent mutations in PIK3CA, TP53, GATA3, MAP3K1 and CDH1, frequent copy-number gains involving CCND1, MYC and FGFR1, and a wide spectrum of fusion events and DNA-repair defects that include BRCA1 and BRCA2 inactivation. The clinical management has accordingly diversified, with endocrine therapy (tamoxifen, the aromatase inhibitors anastrozole, letrozole and exemestane, and the selective oestrogen-receptor degraders fulvestrant and elacestrant) as the cornerstone of hormone-receptor-positive disease, the HER2-directed agents trastuzumab, pertuzumab, ado-trastuzumab emtansine and trastuzumab deruxtecan in HER2-enriched tumours, the cyclin-dependent kinase 4 and 6 (CDK4/6) inhibitors palbociclib, ribociclib and abemaciclib in advanced hormone-receptor-positive disease, poly(ADP-ribose) polymerase (PARP) inhibitors in BRCA-mutated tumours, and immune-checkpoint blockade in PD-L1-positive triple-negative breast cancer. Nevertheless, intrinsic and acquired resistance remains a recurring clinical obstacle in every subtype, driven by ESR1 ligand-binding-domain mutations, activation of PI3K and AKT signalling, HER2 heterogeneity loss, immune escape, and epithelial-to-mesenchymal transition, the latter being particularly pronounced in triple-negative and multidrug-resistant disease [62]. These persistent gaps have made breast cancer one of the most extensively investigated tumour types in network-pharmacology studies of plant-derived agents, and the resulting literature is correspondingly broad both botanically and chemically.

The investigations analysed in this section evaluated a broad range of plant materials studied as crude extracts, solvent fractions or chemically characterised preparations. These included the milkvetch *Astragalus mongholicus* [63], the fritillary *Fritillaria cirrhosa* [64], the Solomon’s seal *Polygonatum sibiricum* [65], the self-heal *Prunella vulgaris* [66], the false bugbane *Actaea vaginata* [67], the spikenard *Aralia chinensis* [68], the tea plant *Camellia sinensis* [69], the heart-leaved moonseed *Tinospora cordifolia* [70], the foxtail *Sophora alopecuroides* [71], the dragon’s blood tree *Dracaena cochinchinensis* [72], the celandine poppy *Hylomecon japonica* [73], the bugleweed *Ajuga decumbens* [74], the spikemoss *Selaginella bryopteris* [75], the wild *Asparagus racemosus* [76], the peacock flower *Caesalpinia pulcherrima* [77], the dyer’s madder *Rubia tinctorum* [78], the Kacip Fatimah *Marantodes pumilum* [79], the Cuban oregano *Coleus amboinicus* [53,80,81], the Malabar spinach *Basella alba* [82], the field pea *Pisum sativum* [83], the *Inula* species *Inula aschersoniana* [84], the corn lily *Gladiolus italicus* [85], the air plant *Kalanchoe laciniata* [86], the loquat *Eriobotrya japonica* [87], the leafy climber *Cissus trifoliata* [51], the Indonesian yellow-root vine *Arcangelisia flava* [88], the *Gymnostachyum* species *Gymnostachyum febrifugum* [89], the African mistletoe *Loranthus micranthus* [90], the hill glory bower *Clerodendrum infortunatum* [91], the larkspur *Delphinium roylei* [92], the snakewood *Rauvolfia tetraphylla* [93], the common dandelion *Taraxacum officinale* [94], the prickly pear *Opuntia ficus-indica* [95], the black cumin *Nigella sativa* [96], the combination of garlic *Allium sativum* and ginger *Zingiber officinale* [97], the sweet wormwood *Artemisia annua* [98], the black pepper *Piper nigrum* [99], the pomegranate *Punica granatum* [100] used together with the beetroot *Beta vulgaris* [101], and the marine seagrass *Enhalus acoroides* [102]. The dominant chemical classes deployed against breast cancer across these studies were flavonoids, with quercetin, kaempferol, apigenin, luteolin, baicalein, hyperoside, isoquercitrin, naringin, myricetin, epigallocatechin-3-gallate, catechin and the bioflavones amentoflavone, lanaroflavone, sequoiaflavone, heveaflavone and the hinokiflavone derivatives appearing most frequently, together with triterpenoids and triterpene saponins such as ursolic, oleanolic and betulinic acids, lupeol, taraxerol and racemosol, phytosterols including β-sitosterol, stigmasterol, γ-sitosterol and campesterol, isoquinoline and indole alkaloids such as berberine, dihydroberberine, ajmaline, reserpine, sanguinarine derivatives, peiminine and sophocarpine, anthraquinones including alizarin, anthragallol and xanthopurpurin, lignans such as sesamin, stilbenoids including resveratrol and pterostilbene, curcuminoids, anthocyanins, betalains, the abietane diterpenoid 16-hydroxy-7α-acetoxyroyleanone and the polysaccharide fraction known as Astragalus polysaccharides.

Across this body of work, the network-pharmacology analyses identified a tightly conserved set of breast cancer hub genes. The serine and threonine protein kinase AKT1 was the single most recurrent target, followed in approximate order of frequency by the epidermal growth factor receptor EGFR, the tumour suppressor TP53, the oestrogen receptor ESR1, the signal transducer and activator of transcription STAT3, the non-receptor tyrosine-kinase SRC, the molecular chaperone HSP90AA1, the mitogen-activated protein kinases MAPK1 and MAPK3, the hypoxia-inducible factor HIF1A, the cyclin-dependent kinases CDK2, CDK4 and CDK6, the catalytic subunit of PI3K (PIK3CA), the matrix metalloproteinase MMP9, the effector caspase CASP3, the antiapoptotic regulator BCL2 and the breast-cancer-specific receptor tyrosine-kinase HER2 (ERBB2). Pathway-enrichment analyses returned the PI3K-Akt signalling pathway as by far the most consistently enriched KEGG term, accompanied by the broader “Pathways in cancer” module, the MAPK signalling pathway, the EGFR tyrosine-kinase inhibitor resistance pathway, the apoptosis pathway, the p53 signalling pathway and a set of breast-cancer-specific axes. These breast-cancer-specific axes comprise the oestrogen signalling pathway, the endocrine-resistance pathway, the steroid hormone biosynthesis pathway, the dedicated “Breast cancer pathway” KEGG entry, the ErbB signalling pathway and the “PD-L1 expression and PD-1 checkpoint pathway in cancer”. Less common but biologically informative pathways, including the Wnt signalling pathway and the Hippo signalling pathway centred on YAP1, also recur in this corpus, indicating that plant compounds active against breast cancer can engage non-canonical oncogenic networks beyond PI3K and MAPK.

Experimental validation in the breast cancer corpus relied predominantly on two human cancer cell lines: the oestrogen-receptor-positive luminal line MCF-7, used in approximately half of the studies discussed here, and the triple-negative line MDA-MB-231, used in a substantial additional proportion. The luminal line T47D and the more aggressive triple-negative line MDA-MB-468 were used in a smaller subset of investigations, while the murine 4T1 line was used in syngeneic immunocompetent transplantation experiments. The non-tumourigenic mammary epithelial line MCF-10A served as the most frequent selectivity control, complemented by the human umbilical vein endothelial cell line (HUVEC), the murine fibroblast line L929, the simian kidney line Vero, the rat pancreatic line RIN-5F, the human embryonic kidney line HEK-293 and the simian kidney line CV-1. The standard in vitro toolkit included MTT or CCK-8 cell-viability assays, scratch and transwell migration or invasion assays, colony-formation assays, Annexin V and propidium iodide or TUNEL-based apoptosis quantification, cell-cycle distribution analysis by flow cytometry, JC-1 mitochondrial-membrane-potential measurements, intracellular reactive-oxygen-species quantification, and Western blot or quantitative PCR validation of the predicted hub genes. In vivo validation was relatively frequent in this corpus. Several investigations advanced to animal models that included: syngeneic 4T1 mammary tumours implanted in BALB/c mice, used to study *Ajuga decumbens* [74] and the *Allium sativum* and *Zingiber officinale* combination [97]; 7,12-dimethylbenz[a]anthracene (DMBA)-induced mammary carcinogenesis in rats and mice, used to study *Nigella sativa* [96], *Caesalpinia pulcherrima* [77], *Rauvolfia tetraphylla* [93] and *Loranthus micranthus* [90]; and the N-methyl-N-nitrosourea (MNU)-induced rat mammary carcinoma model. The relatively substantial in vivo evidence base in this corpus reflects the long-standing availability of well-established mammary carcinogenesis models and provides a stronger translational foundation for the predicted multitarget mechanisms.

Several studies in this corpus introduced innovative methodological angles. The chloroform fraction of *Sophora alopecuroides* [71] reversed multidrug resistance in MCF-7/ADR cells with low micromolar potency and a reversal index of nearly tenfold relative to adriamycin alone, and the prenylated flavonoid kurarinone identified in this fraction exhibited exceptionally favourable docking energies against TNF, AKT1 and CDK2, suggesting that single phytochemicals can simultaneously engage three pharmacologically validated targets. Extracts of *Loranthus micranthus* [90] represent the first phytochemical-based inhibition of the immune-checkpoint enzymes IDO1, IDO2 and TDO2 in the breast cancer setting, restored T-cell viability in co-culture with cancer cells and reduced CTLA-4 expression with concomitant CD4+ T-cell infiltration in DMBA-induced rats, thus connecting plant chemistry to the immune-evasion programmes that limit current immunotherapies. The exosome-like nanoparticles derived from *Polygonatum sibiricum* [65] provided the first nanovesicular delivery proof-of-concept in this corpus; their lipid-bilayered structure of approximately one hundred and forty nanometres in diameter carried eighteen distinct proteins and several hundred small-molecule metabolites, and selectively engaged the oestrogen receptor and PPARG through baicalein, sedanolide and 6-gingerol, as confirmed by 100-nanosecond molecular-dynamics simulations. The flavonoid glycoside fraction of *Kalanchoe laciniata* [86] yielded a quercetin 3-(2-glucosylrhamnoside) with one of the most favourable docking energies reported in this corpus, achieving a value of approximately minus sixteen kilocalories per mole against the breast cancer resistance protein (BCRP) and a strong synergistic interaction with doxorubicin at sub-half-maximal inhibitory concentration (sub-IC50) concentrations. The abietane diterpenoid 16-hydroxy-7α-acetoxyroyleanone, isolated from the leaves of *Coleus amboinicus* [81], achieved a selective IC50 of approximately four micrograms per millilitre against MCF-7 cells with minimal toxicity towards the simian kidney control line CV-1, and exhibited comparable potency against the prostate cancer DU-145 line, providing a single phytochemical with dual breast and prostate cancer activity. Methyl cis-p-coumarate isolated from the leaves and stems of *Pisum sativum* [83] exhibited the highest selectivity index in this corpus, with an IC50 close to one microgram per millilitre against MCF-7 cells and only minimal cytotoxicity to non-tumourigenic mammary epithelial cells. The cyanidin- and betalain-rich extract of *Punica granatum* [100] combined with *Beta vulgaris* [101] acted as a natural radiosensitiser that enhanced the cytotoxicity of low-dose X-rays against triple-negative MDA-MB-231 cells, providing a rationale for integrating phytochemicals with radiotherapy. The prodrug 8-O-acetylharpagide isolated from *Ajuga decumbens* [74] generated two characterised active metabolites in vivo and suppressed AKT, NF-κB and MMP9 signalling in 4T1-bearing BALB/c mice, illustrating how pharmacokinetic transformation can shape the spectrum of network-pharmacology hits. Finally, the *Caesalpinia pulcherrima* [77] ethyl acetate fraction was advanced through an MNU-induced rat mammary carcinoma model with a quantitative histological readout and a marked reduction in oestrogen-receptor-α expression in mammary tissue, providing one of the most thoroughly validated in vivo demonstrations in this corpus.

A comprehensive overview of all breast cancer network-pharmacology studies analysed in this review, with detailed species and plant parts, principal phytoconstituents, predicted hub genes, enriched signalling pathways, in vitro and in vivo experimental models and the principal findings reported by the original investigators, is presented in Table S2 of the Supplementary Materials.

### 4.3. Colorectal Cancer

Colorectal cancer is the third most frequently diagnosed and the second most lethal malignancy worldwide [1]. Most cases arise sporadically through the conventional adenoma to carcinoma sequence, while the hereditary syndromes, including Lynch syndrome and familial adenomatous polyposis, collectively account for approximately five to ten per cent of diagnoses [103]. The molecular taxonomy of colorectal cancer has been refined progressively over the past two decades. Tumours are now stratified into four consensus molecular subtypes, CMS1 to CMS4, that differ in immune infiltration, metabolic reprogramming, mesenchymal phenotype and Wnt-pathway activation, and they are further characterised by recurrent activating mutations in KRAS, BRAF, PIK3CA and SMAD4, by biallelic loss of APC with consequent deregulation of Wnt and β-catenin signalling, and by microsatellite instability of either high (MSI-H) or microsatellite-stable (MSS) status, with the former being observed in approximately fifteen per cent of tumours [104]. Standard systemic management combines fluoropyrimidine-based chemotherapy with oxaliplatin (the FOLFOX regimen) or with irinotecan (the FOLFIRI regimen), antibody-based targeting of EGFR with cetuximab or panitumumab restricted to RAS wild-type tumours, antiangiogenic blockade of vascular endothelial growth factor (VEGF) with bevacizumab or aflibercept, and immune-checkpoint blockade with pembrolizumab or nivolumab approved for MSI-H or mismatch-repair-deficient disease. Despite this expanding armamentarium, five-year survival of metastatic colorectal cancer remains around fourteen per cent, with chemoresistance, KRAS-driven escape from anti-EGFR therapy and the immunologically cold tumour microenvironment of MSS tumours being the principal causes of treatment failure [105]. These limitations have motivated extensive use of in vivo validation in the colorectal cancer network-pharmacology literature, presumably because the field has access to well-characterised chemically induced rodent models that closely recapitulate the human disease.

The investigations analysed in this section evaluated a broad range of plant materials studied as crude extracts, solvent fractions or chemically characterised preparations. These included the kudzu *Pueraria montana* var. *lobata* [106], the skullcap *Scutellaria barbata* [107], the patrinia *Patrinia heterophylla* [108], the peony *Paeonia lactiflora* [109], the garden burnet *Sanguisorba officinalis* [110], the mahonia *Berberis fortunei* [111], the goji *Lycium barbarum* [112], the evodia *Tetradium ruticarpum* [113], the liquorice *Glycyrrhiza uralensis* [114], the combination of *Astragalus mongholicus* [39,63] with the turmeric *Curcuma* [115], the bartsia *Bellardia trixago* [116], the carob *Ceratonia siliqua* [117], the comfrey relative *Paracaryum hedgei* [118], the *Inula* species *Inula aschersoniana* [84], the elmleaf bramble *Rubus ulmifolius* [119], the corn lily *Gladiolus italicus* [85], the bitter leaf *Gymnanthemum amygdalinum* [120], the screw tree *Helicteres isora* [121], the sappanwood *Biancaea sappan* [122], the prickly poppy *Argemone mexicana* [123], the shrub *Homalanthus giganteus* [124], the mangrove *Avicennia alba* [125], the kombucha fermented from the leaves of *Saurauia vulcani* [126], the bark of the gutta-percha tree *Eucommia ulmoides* [127], the triterpenoid-rich roots and stems of *Rhus chinensis* [128,129], the spurge *Euphorbia dentata* [130], the corm-bearing *Gladiolus italicus* [131], the silverberry *Elaeagnus caudata* [54], the beetroot *Beta vulgaris* [101] and the essential oil of the vetiver grass *Chrysopogon zizanioides* [59]. The chemical classes investigated were correspondingly diverse and included flavonoids (notably quercetin, kaempferol, catechin, naringin, rutin, hyperoside, isoquercitrin, myricetin, luteolin, baicalein, isorhamnetin, galangin and hesperidin) and their O-glycosides, triterpenoids and triterpenoid acids (betulinic, betulonic, ursolic, oleanolic, pomolic, maslinic and medicagenic acids), isoquinoline alkaloids (berberine, palmatine, jatrorrhizine, berbamine, chelerythrine, rutaecarpine and evodiamine), homoisoflavonoids, lignans, phenolic acids (rosmarinic acid, salvianolic acid B, caffeic acid, ferulic acid and gallic acid), anthocyanins and betalains (delphinidin 3,5-diglucoside and betanin), plant polysaccharides such as *Lycium barbarum* polysaccharide [112] and Astragalus polysaccharides, and the sesquiterpenoid-dominated essential oil mentioned above.

Across this body of work, the network-pharmacology analyses identified AKT1, EGFR and TP53 as the three most frequently recurring hub genes, with each of these three nodes appearing in the same approximate order of magnitude across the colorectal cancer studies discussed here. Additional broadly recurring hubs included the signal transducer and activator of transcription STAT3, the catalytic subunit of phosphoinositide 3-kinase PIK3CA, the mitogen-activated protein kinases MAPK1 and MAPK3, the apoptosis effector caspase-3, the molecular chaperone HSP90AA1, the inflammatory cytokine TNF, the non-receptor tyrosine-kinase SRC, the antiapoptotic regulator BCL2, the matrix metalloproteinase MMP9, the hypoxia-inducible factor HIF1A and the colorectal-cancer-specific transcription factor β-catenin, encoded by CTNNB1. A distinctive feature of this corpus is the recurrent identification of glycolytic and metabolic enzymes as hub targets. Studies of triterpenoids isolated from *Rhus chinensis* [128,129] and of the *Astragalus mongholicus* and *Curcuma* [115] combination repeatedly highlighted enolase 1 (ENO1), fructose-bisphosphate aldolase A (ALDOA), 6-phosphofructo-2-kinase/fructose-2,6-biphosphatase 3 (PFKFB3), pyruvate kinase M2 (PKM2) and lactate dehydrogenase A (LDHA), suggesting that plant compounds in this corpus also engage the Warburg-type metabolic reprogramming that characterises gastrointestinal tumours. Pathway-enrichment analyses returned the PI3K-Akt signalling pathway as the most consistently enriched KEGG term across the colorectal cancer studies, followed by the broader “Pathways in cancer” module, the MAPK signalling pathway, the apoptosis pathway, the TNF signalling pathway, the EGFR tyrosine-kinase inhibitor resistance pathway, the HIF-1 signalling pathway, the p53 signalling pathway, the cell-cycle module, and two colorectal-cancer-specific axes, namely the dedicated “Colorectal cancer pathway” KEGG entry and the Wnt signalling pathway. The latter axis is particularly significant because it directly mirrors the central role of APC and β-catenin in the molecular pathogenesis of the disease.

Experimental validation in the colorectal cancer corpus relied on a broad panel of cell lines that reflects the molecular heterogeneity of the disease. The HCT-116 cell line, characterised by KRAS and PIK3CA mutations, microsatellite instability and an aggressive phenotype, was the most frequently used; HT-29, an aggressively BRAF-mutated and microsatellite-stable line, was used in a similar proportion of investigations. The KRAS-mutated SW480 and SW620 lines were used together by several research groups to compare matched primary and metastatic tumour cells, while the EGFR-driven LoVo line, the BRAF-mutated WiDr line, the COLO 320DM colorectal cell line, the doxorubicin-sensitive COLO 205 line and the doxorubicin-resistant COLO 320 line (used together as a sensitive and resistant pair in the *Homalanthus giganteus* [124] study), the KM12SM and MC38 lines (the latter used in syngeneic immunocompetent experiments) and the CT26 line (used in orthotopic models) were also represented. Non-tumourigenic FHC and NCM460 colonocytes were the principal selectivity controls, together with HEK-293, WI-38, L929 and HDFn cells used in additional cytocompatibility assays. In vivo validation was particularly frequent in this corpus. The animal models employed comprised chemically induced colorectal carcinogenesis, including: azoxymethane/dextran sulfate sodium (AOM/DSS)-induced colitis-associated carcinoma in Kunming and C57BL/6 mice (*Paeonia lactiflora* [109], *Sanguisorba officinalis* [110], *Gymnanthemum amygdalinum* [120] and the liquorice-extract study of *Glycyrrhiza* species [132]) and benzo[a]pyrene-induced damage in rats (*Saurauia vulcani* [126] fermented as kombucha); subcutaneous xenografts in BALB/c nude mice using HCT-116, the oxaliplatin-resistant HCT-116-OXR, SW480 and SW620 cells; orthotopic CT26-luciferase implantation in BALB/c mice (*Astragalus mongholicus* and *Curcuma* [115] combination); syngeneic MC38 implantation in C57BL/6 mice (*Biancaea sappan* [122]); and an embryonic zebrafish xenograft model used in parallel with rodent experiments in the liquorice-extract study [132]. This breadth of in vivo experimentation, together with the metabolic and inflammatory mechanisms uncovered in those models, supports the translational maturity of this corpus.

Several studies in this corpus warrant individual discussion because of the breadth or originality of their experimental validation. The liquorice-extract study [132] built upon serum pharmacochemistry by ultra-high-performance liquid chromatography with high-resolution mass spectrometry, identifying more than one hundred plant-derived compounds in the extracts and several dozen absorbed bloodstream prototypes or metabolites; the authors then validated the predicted activity simultaneously in an AOM/DSS C57BL/6J mouse model and in an HCT-116 zebrafish xenograft model, and confirmed mechanistic specificity through a pharmacological rescue experiment in which the RAS activator ML-099 reversed the inhibitory effect of the liquorice serum on the RAS, RAF, MEK and extracellular signal-regulated kinase (ERK) signalling cascade. The *Lycium barbarum* [112] polysaccharide study reversed acquired oxaliplatin resistance in HCT-116-OXR cells through coordinate downregulation of phosphomannose isomerase and the ATP-binding cassette transporter ABCG2, with concomitant tumour shrinkage in an HCT-116-OXR xenograft model in immunodeficient mice. The combination of *Astragalus mongholicus* with *Curcuma* [115] normalised tumour-associated endothelial junctions and inhibited tumour glycolysis through direct binding of HIF-1α and modulation of PFKFB3 in an orthotopic CT26-luciferase model in BALB/c mice. The heartwood extract of *Biancaea sappan* [122] suppressed STAT3 and AKT phosphorylation, induced caspase-3 and caspase-7-dependent apoptosis across four colorectal cancer cell lines and reduced MC38 syngeneic tumour weight in C57BL/6 mice at an oral dose of one hundred milligrams per kilogram, without measurable organ toxicity. The processed triterpenoid fraction of *Sanguisorba officinalis* [110] suppressed TNF-α and NF-κB signalling in AOM/DSS mice and uniquely induced hypermethylation of the TNF-α and p65 gene promoters, adding an epigenetic dimension that was absent in most other studies. Phytochemical investigation of *Euphorbia dentata* [130] yielded a structurally novel tigliane diterpene named Dentatacid A, characterised by an unprecedented 2,3-seco-arbor-2,3-dioic skeleton and showing low-micromolar cytotoxic activity against HT-29 cells. *Berberis fortunei* [111] revealed a structurally driven differential targeting in which protoberberine alkaloids preferentially engaged cyclin D1 (CCND1) and HSP90AA1, whereas bisbenzylisoquinoline alkaloids bound AKT1 and the small GTPase CDC42, illustrating how chemical scaffolds shape target-selection patterns. The tigliane diterpene 4 isolated from *Homalanthus giganteus* [124] was equipotent against the doxorubicin-sensitive COLO 205 line and the doxorubicin-resistant COLO 320 line, supported by density-functional-theory calculations and a clear preference for protein kinase C-α and glycogen-synthase kinase 3-β as molecular targets. The rosmarinic acid identified in *Helicteres isora* [121] extracts achieved a docking energy against MAPK1 that exceeded that of the reference BRAF inhibitor encorafenib, and a Cancer Genome Atlas (TCGA)-based survival analysis associated higher MAPK1 expression with improved overall survival in colorectal cancer patients. Finally, the kombucha prepared by fermenting *Saurauia vulcani* [126] leaves examined a fermented plant product, with the authors reporting potential immune cross-reactivity between *Lactiplantibacillus plantarum* and *Saccharomyces cerevisiae* epitopes and colorectal-cancer-associated antigens, opening a chemopreventive avenue.

A comprehensive overview of all colorectal cancer network-pharmacology studies analysed in this review, with detailed species and plant parts, principal phytoconstituents, predicted hub genes, enriched signalling pathways, in vitro and in vivo experimental models and the principal findings reported by the original investigators, is presented in Table S3 of the Supplementary Materials.

### 4.4. Prostate Cancer

Prostate cancer is the fourth most frequently diagnosed and the eighth most lethal malignancy worldwide [1]. Histologically, more than ninety-five per cent of tumours are acinar adenocarcinomas, graded on the Gleason scoring system and clinically managed according to risk stratification that integrates serum prostate-specific antigen, clinical T stage and the Gleason score [133]. The androgen receptor signalling axis, encoded by the AR gene, is the central oncogenic driver throughout the entire disease continuum. As a result, androgen-deprivation therapy using luteinizing hormone-releasing hormone (LHRH) analogues, AR antagonists and inhibitors of the steroidogenic enzyme CYP17A1 (enzalutamide, apalutamide, darolutamide and abiraterone) remains the cornerstone of systemic treatment, complemented by docetaxel and cabazitaxel taxane chemotherapy, the bone-seeking radioisotope radium-223, PARP inhibitors (olaparib and rucaparib) for BRCA-mutated disease, and 177Lu-PSMA-617 (prostate-specific membrane antigen, PSMA) radioligand therapy for PSMA-positive castration-resistant disease [134]. Despite this expanding armamentarium, almost all metastatic prostate cancers eventually progress to castration-resistant disease and, ultimately, to lethal, androgen-receptor-independent neuroendocrine variants. The principal resistance mechanisms include AR amplification, the expression of AR splice variants such as AR-V7 that lack the ligand-binding domain, the loss of PTEN and TP53, alterations in BRCA1, BRCA2 and the mismatch-repair machinery, and lineage plasticity that drives epithelial cells towards neuroendocrine differentiation. These resistance mechanisms motivate the search for chemically diverse, multitarget plant agents that can act either on the canonical androgen-receptor axis or on AR-independent oncogenic nodes, ideally engaging both in a single pharmacological mixture.

The investigations analysed in this section evaluated a chemically and botanically heterogeneous selection of plants studied as crude extracts, solvent fractions, individual phytochemicals or, in a single case, a complex volatile essential oil. These included the steam-distilled essential oil of the timber tree *Phoebe zhennan* [135], a clinically used herb pair [136] of *Scleromitrion diffusum* [46] and *Scutellaria barbata* [107], the sweet osmanthus *Osmanthus fragrans* [137], the simpleleaf chaste tree *Vitex trifolia* [138], the *impatiens Impatiens balsamina* [139], the daisy-like *Aspilia pluriseta* [140], the legume *Tetrapleura tetraptera* [141], the candelabra spurge *Euphorbia ingens* [142], the rosemary *Salvia rosmarinus* [143], the corn lily *Gladiolus italicus* [85], the perilla *Perilla frutescens* [144], the aromatic herb *Coleus amboinicus* [81], the sweet wormwood *Artemisia annua* [98] and the leafy climber *Cissus trifoliata* [51]. The dominant chemical classes investigated were: triterpenoids and triterpenoid acids, including ursolic acid, lanosterol, oleanolic acid, squalene and the abietane diterpene 16-hydroxy-7α-acetoxyroyleanone; flavonoids such as luteolin, apigenin, baicalein, quercetin, scutellarin, hyperoside, isoquercitrin, kaempferol-3-glucoside, myricetin and rutin; sesquiterpene-rich essential oils enriched in α-eudesmol, γ-eudesmol, humulene, elemol and α-copaen-11-ol; the diterpene andrographolide; phenolic acids including rosmarinic acid and the rosemary-specific diterpenes carnosic acid and carnosol; iridoid glycosides such as agnuside; steroidal alkaloids including solasodine and peimine; anthocyanins; and an artemisinin-related sesquiterpene lactone fraction.

Across this body of work, network-pharmacology analyses identified AKT1 as the single most recurrent hub gene, followed in approximate order of recurrence by the epidermal growth factor receptor EGFR, the tumour suppressor TP53, the oestrogen receptor ESR1, and the prostate-cancer-specific androgen receptor AR. Additional broadly recurring nodes included the mitogen-activated protein kinases MAPK1 and MAPK3, the antiapoptotic regulator BCL2, the regulatory subunit of phosphoinositide 3-kinase PIK3R1, the matrix metalloproteinases MMP9 and MMP2, the apoptosis effector caspase-3, the cell-cycle regulators CCND1 and CDK2, and a set of receptor-tyrosine kinases of the platelet-derived growth factor family (PDGFRA and PDGFRB), the non-receptor tyrosine-kinase FYN, the HER2 receptor encoded by ERBB2, the proto-oncogene MYC, the transcription factor NF-κB1, the activator-protein-1 component JUN and the nuclear receptor PPARG. The recurrent identification of AR, PDGFR isoforms, FYN and the activating transcription factor ATF3 (the latter identified by the *Impatiens balsamina* [139] study as a critical mediator of androgen-receptor downregulation) is a characteristic feature of this corpus, and it indicates that plant compounds in this set engage both the androgen-receptor axis and selected receptor-tyrosine-kinase and integrin-signalling modules. Pathway-enrichment analyses returned the PI3K-Akt signalling pathway as one of the most consistently enriched KEGG terms, followed by the prostate-cancer-specific “Prostate cancer pathway” module, the MAPK and p53 signalling pathways, the EGFR tyrosine-kinase inhibitor resistance pathway, the apoptosis pathway, the TNF and NF-κB signalling pathways, the endocrine-resistance pathway, the AMP-activated protein kinase (AMPK) signalling pathway, the steroid biosynthesis pathway and the “PD-L1 expression and PD-1 checkpoint pathway in cancer”.

Experimental validation in the prostate cancer corpus relied on a relatively narrow panel of cell lines. The androgen-receptor-negative, castration-resistant line DU-145 was by far the most frequently used; the equally androgen-receptor-negative and bone-metastatic line PC-3 was used in a substantial additional proportion of studies; the androgen-receptor-positive, androgen-responsive line LNCaP was used in a smaller number of investigations; and the castration-resistant 22Rv1 line, which expresses AR-V7 splice variants, was used in a single comprehensive study. Non-tumourigenic controls included the simian kidney epithelial line Vero E6, the human prostatic epithelial line PNT2, the simian kidney line CV-1 and the murine macrophage line RAW 264.7, with the latter used additionally in anti-inflammatory experiments. The standard in vitro readouts included MTT or CCK-8 viability assays, scratch and transwell migration assays, Annexin V and propidium iodide or TUNEL apoptosis assays, flow-cytometric cell-cycle analysis and Western blot or quantitative reverse-transcription PCR (qRT-PCR) validation of the predicted hub genes, including the androgen receptor AR, the antiapoptotic regulator BCL2, the tumour suppressor p53, the apoptosis effector caspase-3 and the cell-cycle regulator CDK1. In vivo validation was particularly limited in this network-pharmacology corpus. Only one investigation reported an established xenograft tumour model, namely the PC-3 xenograft in BALB/c nude mice for the *Impatiens balsamina* [139] semen extract. A second study used male Sprague–Dawley rats for pharmacokinetic profiling of absorbed *Vitex trifolia* [138] metabolites without including a tumour model. This limited in vivo evidence base represents a notable gap in this network-pharmacology corpus.

Several investigations within this corpus warrant individual discussion because of the breadth of their experimental support. The ethyl acetate extract of *Impatiens balsamina* [139] semen is the only investigation in this corpus that combined four representative prostate cancer cell lines (LNCaP, 22Rv1, PC-3 and DU-145), demonstrated induction of G0/G1 arrest and apoptosis across both androgen-receptor-positive and androgen-receptor-negative contexts, identified the activating transcription factor 3 (ATF3) as a critical mediator of androgen-receptor downregulation, and confirmed in vivo efficacy in a PC-3 xenograft mouse model. The steam-distilled essential oil of *Phoebe zhennan* [135], enriched in sesquiterpenes at approximately seventy-eight per cent of total composition, achieved exceptionally low IC50 values on the LNCaP and PC-3 cell lines (in the sub-microlitre per millilitre range); the oil also induced G2/M arrest in LNCaP cells and G0/G1 arrest in PC-3 cells, and humulene, α-copaen-11-ol and elemol were identified as the top docking partners of AKT1. The clinically used herb pair [136] of *Scleromitrion diffusum* [46] and *Scutellaria barbata* [107] identified more than one hundred shared targets with AKT1, BCL2 and NF-κB1 as the core axis and confirmed in 100-nanosecond molecular-dynamics simulations the AKT1-apigenin complex as the most stable, while ursolic acid emerged as the strongest binder of AKT1 by docking; the herb pair inhibited proliferation and migration and induced apoptosis in PC-3 cells at sub-millimolar potency. The single abietane diterpenoid 16-hydroxy-7α-acetoxyroyleanone, isolated from *Coleus amboinicus* [81], achieved a low-microgram-per-millilitre IC50 on DU-145 cells with a selectivity index exceeding that of cisplatin and bound the matrix metalloproteinase MMP2, the nuclear receptor PPARG and the antiapoptotic regulator BCL2 through key hydrogen-bond interactions. The *Salvia rosmarinus* [143] extracts and the constituents carnosol, carnosic acid and rosmarinic acid were specifically positioned as candidates for the treatment of androgen-receptor-negative prostate cancer through binding to EGFR, ERBB2 and TP53, providing a rationale for AR-independent therapeutic strategies. Finally, the *Tetrapleura tetraptera* [141] fruit extract and the constituents luteolin and scopoletin showed selective cytotoxicity against PC-3 and LNCaP cells with sparing of the normal prostatic epithelial line PNT2 and identified MMP9 and the androgen receptor as the principal docking targets.

A comprehensive overview of all prostate cancer network-pharmacology studies analysed in this review, with detailed species and plant parts, principal phytoconstituents, predicted hub genes, enriched signalling pathways, in vitro and in vivo experimental models and the principal findings reported by the original investigators, is presented in Table S4 of the Supplementary Materials.

A consolidated cross-cancer comparison of the principal hub-gene families and signalling pathways for each cancer type is presented in Table 1.

## 5. Key Molecular Targets and Signalling Pathways

**Figure 4:**
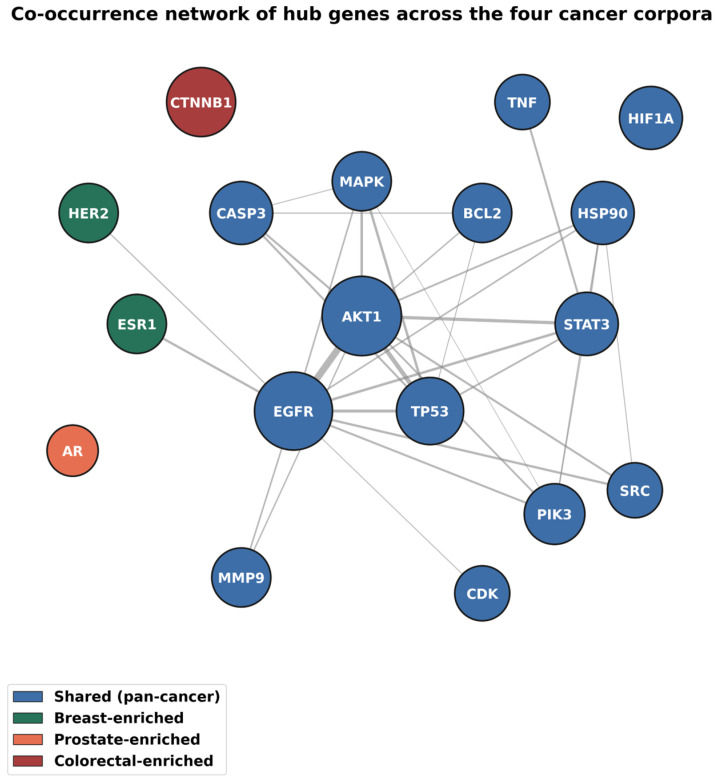
Co-occurrence network of the most frequently identified hub genes across the 101 reviewed plant-derived network-pharmacology studies. Nodes are hub genes (node size proportional to the total number of studies that identified the gene across all four cancer corpora) and edges connect genes that co-occur as hub targets in at least three studies (edge width proportional to the co-occurrence count). Node colour reflects the dominant cancer-type association: shared (blue), breast-enriched (green), prostate-enriched (orange) or colorectal-enriched (red). Selected gene labels are abbreviated for readability (MAPK = MAPK1 and MAPK3; HSP90 = HSP90AA1; PIK3 = PIK3CA and PIK3R1; HER2 = HER2 and ERBB2; CDK = CDK2, CDK4 and CDK6).

Comparative analysis of the hub-gene rosters reported across the studies reviewed here reveals a striking convergence on a small set of master regulators that recur in all four cancer types, while also identifying tumour-specific signatures that distinguish the four corpora. These convergent and cancer-specific hub-gene relationships are visualised as a co-occurrence network in Figure 4.

The single most frequently identified hub gene across the entire review is the serine and threonine protein kinase AKT1, which appeared as a central node in lung cancer studies of *Anemarrhena asphodeloides* [38], *Astragalus mongholicus* [39], *Panax ginseng* [42] and *Pinellia ternata* [44], in breast cancer investigations of *Eriobotrya japonica* [87], *Fritillaria cirrhosa* [64], *Asparagus racemosus* [76] and *Sophora alopecuroides* [71], in colorectal cancer studies of *Lycium barbarum* [112], *Inula aschersoniana* [84] and *Patrinia heterophylla* [108], and in prostate cancer studies of *Phoebe zhennan* [135] and the *Scleromitrion diffusum* and *Scutellaria barbata* herb pair [136]. The epidermal growth factor receptor EGFR is the second most recurrent target, with prominent identification in lung cancer studies of *Anemarrhena asphodeloides* [38], *Glycyrrhiza glabra* [40] and the Amaryllidaceae-type alkaloid-yielding plants *Crinum bulbispermum*, *Pancratium maritimum* and *Hippeastrum vittatum* [57], in breast cancer studies of *Marantodes pumilum* [79] and *Inula aschersoniana* [84], in colorectal cancer studies of *Rubus ulmifolius* [119], *Patrinia heterophylla* [108] and *Avicennia alba* [125], and in prostate cancer studies of *Tetrapleura tetraptera* [141] and *Salvia rosmarinus* [143]. The tumour suppressor TP53 emerges as the third most pervasive hub gene, identified in lung cancer studies of *Cinnamomum tamala* [58] and *Taraxacum officinale* [56], in breast cancer studies of *Arcangelisia flava* [88], *Clerodendrum infortunatum* [91] and *Gladiolus italicus* [85], in colorectal cancer studies of *Tetradium ruticarpum* [113] and *Bellardia trixago* [116], and in prostate cancer studies of *Vitex trifolia* [138] and *Impatiens balsamina* [139]. Additional broadly shared hubs include: the signal transducer and activator of transcription STAT3, identified in *Anemarrhena asphodeloides* [38], *Cinnamomum tamala* [58], *Sophora alopecuroides* [71] and the *Glycyrrhiza* species liquorice extract [132]; the mitogen-activated protein kinases MAPK1 and MAPK3; the apoptosis effector caspase-3; the molecular chaperone HSP90AA1; the regulatory and catalytic subunits of phosphoinositide 3-kinase PIK3R1 and PIK3CA; the antiapoptotic regulator BCL2; the non-receptor tyrosine-kinase SRC; and the cytokine TNF, all of which appear across multiple studies in each of the four cancer types. In contrast, hormone-receptor nodes are clearly enriched in cancers that depend on steroid signalling: the oestrogen receptor ESR1 dominates the breast cancer corpus, with prominent identification in *Camellia sinensis* [69], *Asparagus racemosus* [76] and *Caesalpinia pulcherrima* [77], and recurs in prostate cancer in *Phoebe zhennan* [135], *Vitex trifolia* [138] and *Osmanthus fragrans* [137], while the HER2 receptor encoded by ERBB2 emerges in breast cancer studies of *Enhalus acoroides* [102] and in prostate cancer studies of *Salvia rosmarinus* [143]. The androgen receptor AR is identified almost exclusively in prostate cancer, where it appears as a critical node in *Impatiens balsamina* [139], *Osmanthus fragrans* [137], *Tetrapleura tetraptera* [141] and *Vitex trifolia* [138]. Finally, the Wnt-pathway transcription factor CTNNB1 (β-catenin) is identified as a hub gene almost exclusively in colorectal cancer, notably in the *Saurauia vulcani* kombucha [126], *Bellardia trixago* [116] and *Glycyrrhiza* species liquorice extract [132] studies, in line with the central role of APC and β-catenin dysregulation in the adenoma-to-carcinoma sequence [103].

At the pathway level, an even stronger convergence emerges. The PI3K-Akt signalling pathway is the single most enriched KEGG term [32] across the entire review and recurs in nearly every cancer-specific section. It is identified, for example, in the lung cancer studies of *Astragalus mongholicus* [39], *Coreopsis tinctoria* [52] and *Dioscorea zingiberensis* [45], in breast cancer studies of *Arcangelisia flava* [88], *Aralia chinensis* [68] and *Hylomecon japonica* [73], in colorectal cancer studies of *Beta vulgaris* [101], *Sanguisorba officinalis* [110] and *Biancaea sappan* [122], and in prostate cancer studies of *Aspilia pluriseta* [140] and *Euphorbia ingens* [142]. The MAPK signalling pathway is the second most pervasive, identified in the *Cinnamomum tamala* study [58], the *Helicteres isora* rosmarinic acid study [121] and the *Salvia rosmarinus* rosemary investigation [143], among many others. The apoptosis pathway and the p53 signalling pathway co-occur with PI3K/AKT and MAPK in most studies and frequently involve the downstream regulators BCL2, BAX, caspase-3 and PARP, exemplified by *Coreopsis tinctoria* [52], *Caesalpinia pulcherrima* [77] and *Astragalus mongholicus* polysaccharides [63], in line with the long-established role of intrinsic apoptosis as a tractable cancer cell vulnerability [11,12]. The NF-κB signalling pathway and the inflammation-linked TNF and IL-17 signalling pathways are particularly prominent in colorectal and breast cancer studies that used chemically induced in vivo models such as the AOM/DSS-induced colitis-associated carcinoma model employed for *Paeonia lactiflora* [109], *Sanguisorba officinalis* [110], *Gymnanthemum amygdalinum* [120] and the *Glycyrrhiza* species liquorice extract [132], or the DMBA-induced mammary carcinogenesis model used for *Nigella sativa* [96], *Caesalpinia pulcherrima* [77], *Rauvolfia tetraphylla* [93] and *Loranthus micranthus* [90]. The HIF-1 signalling pathway recurs in studies that demonstrated angiogenic, metabolic or hypoxia-driven effects, notably in the *Astragalus mongholicus* and Curcuma combination that normalised tumour-associated endothelial junctions through direct HIF-1α binding and PFKFB3 modulation [115], and in the *Polygonatum sibiricum* exosome-like nanoparticle study [65]. The Wnt and β-catenin signalling pathway is the only one of these core pathways that is unevenly distributed across the four corpora, being primarily reported in colorectal cancer studies such as *Saurauia vulcani* kombucha [126] and *Bellardia trixago* [116], and only sporadically in breast or lung cancer studies, again consistent with the central role of APC mutations in colorectal carcinogenesis [103].

Beyond this shared core, each cancer type displayed a distinct molecular signature in the network-pharmacology literature. Lung cancer studies highlighted the EGFR/RAS/MAPK and EGFR/PI3K/AKT axes that are also targeted by clinically approved tyrosine-kinase inhibitors [36], together with the KEGG entries “Non-small cell lung cancer” and “EGFR tyrosine-kinase inhibitor resistance”, as illustrated by *Anemarrhena asphodeloides* [38] and *Glycyrrhiza glabra* [40], and frequently included epithelial-to-mesenchymal transition effectors such as MMP9 and the SNAI family transcription factors, exemplified by *Dioscorea zingiberensis* [45] and *Scleromitrion diffusum* [46]. Breast cancer studies were uniquely enriched in hormone- and steroid-dependent signalling, with recurring identification of ESR1, ESR2 and ERBB2, and of the KEGG entries “Estrogen signalling pathway”, “Endocrine resistance”, “Steroid hormone biosynthesis”, “Breast cancer pathway” and the “PD-L1 expression and PD-1 checkpoint pathway in cancer” [62,63]; these axes are exemplified by *Camellia sinensis* [69], *Inula aschersoniana* [84], *Prunella vulgaris* [66] and *Caesalpinia pulcherrima* [77]. The breast cancer corpus also engaged the non-canonical Hippo and YAP1 signalling axis through *Rauvolfia tetraphylla* [93] and the Wnt signalling pathway through *Tinospora cordifolia* [70], illustrating the methodological power of network pharmacology to capture the engagement of less canonical oncogenic networks. Colorectal cancer studies were distinguished by recurrent identification of CTNNB1, by the KEGG entries “Colorectal cancer pathway” and “Wnt signalling pathway”, by the engagement of glycolytic and metabolic enzymes (ENO1, ALDOA, PFKFB3, PKM2 and LDHA), and by inflammation-linked TNF, NF-κB and IL-17 signalling pathways captured by AOM/DSS-induced or benzo[a]pyrene-induced models. The metabolic axis is particularly well exemplified by the *Rhus chinensis* triterpenoid studies [128,129] and the *Astragalus mongholicus* and *Curcuma* combination [115]. Prostate cancer studies were characterised by the AR axis, including AR amplification, AR-V7 splice variants and ATF3-mediated AR downregulation, with the latter identified in *Impatiens balsamina* [139]; by receptor-tyrosine kinases of the platelet-derived growth factor family identified in *Perilla frutescens* [144]; by integrins such as ITGB3; and by the prostate-cancer-specific KEGG entry “Prostate cancer pathway” together with the “Steroid biosynthesis” pathway, which is directly relevant to androgen biosynthesis and metabolism [133].

**Figure 5:**
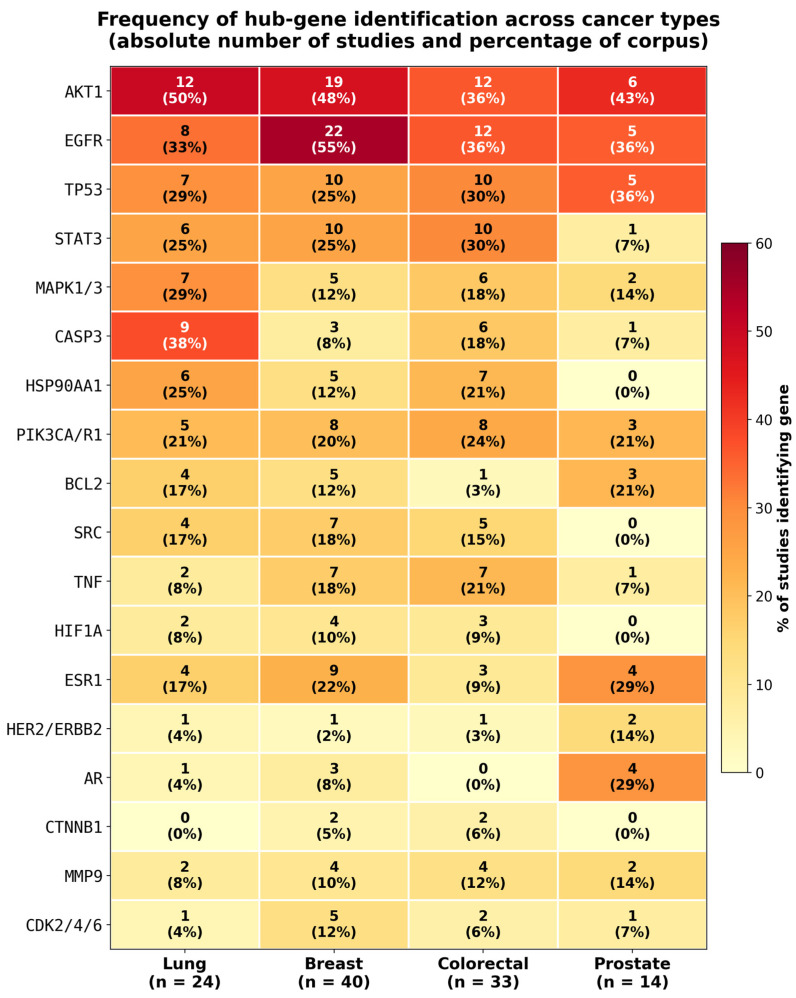
Cross-cancer frequency of hub-gene identification across the 101 plant-derived network-pharmacology studies reviewed. Each cell reports the absolute number of studies (top) and the percentage of the cancer-specific corpus (in parentheses) that identified the corresponding gene as a hub target. Colour intensity is proportional to the percentage of studies in each cancer-specific corpus.

The molecular targets identified across the studies reviewed here do not act in isolation but are organised in a tightly interconnected oncogenic network, the topology of which is increasingly understood from systems-level analyses of cancer cell signalling [6]. The PI3K/AKT/mTOR and MAPK/ERK axes communicate at multiple nodes, including RAS as an upstream activator of both branches, GSK3β as a downstream substrate of AKT and a regulator of MAPK-dependent transcription, and the feedback loop in which mTORC1 represses upstream PI3K signalling. PI3K/AKT and the p53 axis intersect through MDM2, whose stability is regulated by AKT-dependent phosphorylation, while p53 in turn induces PTEN expression and thus dampens PI3K activity [11]. The NF-κB and STAT3 transcription factors are activated downstream of TNF and interleukin-6 (IL-6) and converge on inflammation-driven proliferation, survival and immune evasion, providing a mechanistic link between chronic inflammation and the AOM/DSS or DMBA models used in the reviewed in vivo studies. The *Sanguisorba officinalis* triterpenoid study [110] is a particularly compelling example of how plant compounds can suppress both NF-κB activity and the upstream TNF cytokine in vivo. Wnt and β-catenin signalling is itself regulated by GSK3β, placing this colorectal-cancer-specific axis under the indirect control of PI3K/AKT. HIF-1α stability is regulated by PI3K/AKT and feeds into both angiogenesis through VEGFA [19,20] and glycolytic metabolism through PFKFB3 and LDHA, a connection directly demonstrated in the *Astragalus mongholicus* and *Curcuma* combination study [115]. Finally, the androgen-receptor axis in prostate cancer is reciprocally regulated by PI3K/AKT through PTEN loss, providing the rationale for combining androgen-deprivation therapy with PI3K or AKT inhibitors in resistant disease [133]. Plant-derived compounds, by virtue of their inherent polypharmacology [5,7], can engage several of these interconnected hubs simultaneously, which is the mechanistic justification for using network pharmacology to study them [8]. The frequency with which each hub gene was identified in every cancer-specific corpus is summarised in Figure 5.

## 6. Major Classes of Plant-Derived Anticancer Compounds

**Figure 6:**
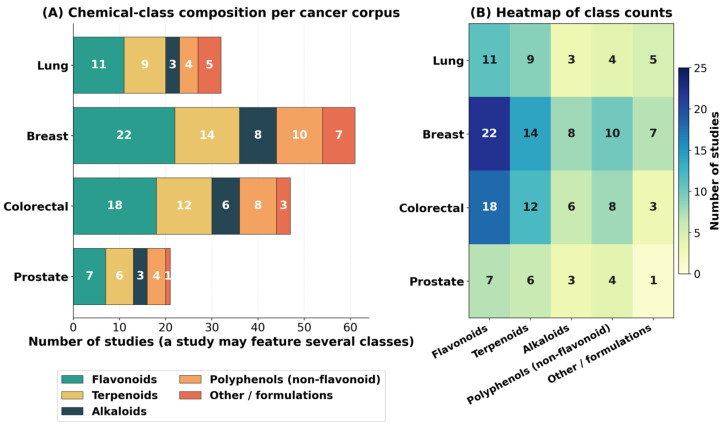
Distribution of the principal phytochemical classes across the four reviewed cancer types. (**A**) Stacked horizontal bar chart showing the number of studies in each cancer corpus that featured each major chemical class (flavonoids, terpenoids, alkaloids, polyphenols other than flavonoids, and other bioactive compounds). (**B**) Heatmap of the same data with absolute counts annotated in each cell. A study that featured several classes was counted once in each class.

The studies analysed in this review cumulatively investigated several hundred individual phytochemicals from more than seventy plant species [5,7]. Although the chemical landscape is diverse, the active constituents and mechanisms of action repeatedly belong to a small number of structural classes, and the same compounds frequently emerge as active principles in studies of different plants. The five most frequently represented classes in this review are alkaloids, flavonoids, terpenoids, polyphenols other than flavonoids, and a heterogeneous group of additional bioactive compounds and formulations. Their distribution across cancer types and the principal mechanisms identified by network pharmacology are summarised in the following subsections. The distribution of these principal chemical classes across the four reviewed cancer types is shown in Figure 6.

### 6.1. Alkaloids

Alkaloids are nitrogen-containing heterocyclic secondary metabolites with a long-established role in clinical oncology, most notably through the vinca alkaloids vinblastine and vincristine and the camptothecin analogues topotecan and irinotecan [4,5]. In the corpus reviewed here, several alkaloid classes were investigated. The *Berberis fortunei* study [111] demonstrated a structurally driven differential targeting in which protoberberine alkaloids (berberine, palmatine, and jatrorrhizine) preferentially bound CCND1 and HSP90AA1, while bisbenzylisoquinoline alkaloids (berbamine, fangchinoline, and tetrandrine) bound AKT1 and the small GTPase CDC42 in colorectal cancer. The Amaryllidaceae-type alkaloids crinamine, ismine, lycorine and hemanthidine isolated from *Crinum bulbispermum*, *Pancratium maritimum*, *Hippeastrum vittatum* and *Centaurea scoparia* [57] displayed multitarget activity against AR, EGFR and ESR1 in non-small-cell lung cancer. The quinolizidine alkaloid sophocarpine isolated from *Sophora alopecuroides* [71] reversed multidrug resistance in adriamycin-resistant MCF-7/ADR cells, while peiminine from *Fritillaria cirrhosa* [64], 6-methoxydihydrosanguinarine from *Hylomecon japonica* [73], reserpine and ajmaline from *Rauvolfia tetraphylla* [93] and chelerythrine from *Argemone mexicana* [123] were associated with PI3K/AKT, p53 and NF-κB modulation in breast and colorectal cancer. The pyridoindole alkaloids rutaecarpine and evodiamine isolated from *Tetradium ruticarpum* [113] bound TNF-α and AKT1 in colorectal cancer. These alkaloid studies collectively illustrate how scaffold diversity within a single chemical class can generate distinct target-engagement profiles, a property that lends itself naturally to the multitarget analysis performed by network pharmacology [8,17].

### 6.2. Flavonoids

Flavonoids constitute the most abundantly represented chemical class in this review, with quercetin, kaempferol, luteolin and apigenin reported as bioactive constituents across all four cancer types. Quercetin is identified in studies of *Coreopsis tinctoria* [52], *Achyrocline satureioides* [49], *Basella alba* [82], *Nandina domestica* [50] and *Beta vulgaris* [101], and kaempferol in studies of *Eucommia ulmoides* [127], *Anemarrhena asphodeloides* [38] and *Asparagus racemosus* [76]. Additional flavonoids and glycosides repeatedly identified by network pharmacology include epigallocatechin-3-gallate from *Camellia sinensis* [69], catechin from *Rubus ulmifolius* [119], baicalein and scutellarin from *Scutellaria barbata* [107], naringin from *Inula aschersoniana* [84], rutin from *Bellardia trixago* [116], hyperoside, isoquercitrin and myricetin from *Gladiolus italicus* [85], the flavonol glycoside kaempferol-3-O-glucopyranoside that displayed an exceptionally favourable docking energy against PI3K in the *Gladiolus italicus* colorectal cancer study [131], and the dietary flavonoids galangin and hesperidin from *Beta vulgaris* [101]. The bioflavones amentoflavone, lanaroflavone, sequoiaflavone, heveaflavone and the hinokiflavone derivatives isolated from *Selaginella bryopteris* [75] bound EGFR, TP53, STAT3 and VEGFA in breast cancer. Across studies, flavonoids preferentially engaged the PI3K/AKT, MAPK and apoptosis axes, modulated p53 and BCL2 family proteins, and frequently displayed antioxidant and anti-inflammatory effects through suppression of NF-κB and STAT3 [15,22]. Several flavonoid glycosides showed unusually high docking affinities, exemplified by quercetin 3-(2-glucosylrhamnoside) from *Kalanchoe laciniata* [86] binding the BCRP transporter and AKT1 with markedly favourable energies, and by isoquercitrin from *Gladiolus italicus* [85] engaging FGFR2 with stable molecular-dynamics complexes.

### 6.3. Terpenoids

Terpenoids were the second most represented class and encompassed several pharmacologically distinct subclasses. Triterpenoids and triterpenoid acids, including ursolic acid, oleanolic acid, betulinic acid, betulonic acid, taraxerol, racemosol, pomolic acid, maslinic acid, medicagenic acid and lupeol, appeared in most studies as multitarget agents engaging AKT1, BCL2, MAPK1, caspase-3 and the apoptosis axis [18]. Examples include ursolic acid in the *Cissus trifoliata* investigation [51] and in the *Scleromitrion diffusum* and *Scutellaria barbata* herb pair against prostate cancer [136], oleanolic acid and ursolic acid in *Prunella vulgaris* [66], lupeol in *Gymnostachyum febrifugum* [89], the *Rhus chinensis*-derived betulinic and betulonic acids that engaged glycolytic enzymes in colorectal cancer [128,129], and taraxerol from *Taraxacum officinale* that suppressed migration and invasion in triple-negative breast cancer through the ERK and Slug axis [94]. The triterpene saponin dioscin from *Dioscorea zingiberensis* reversed epithelial-to-mesenchymal transition in lung adenocarcinoma through inactivation of the AKT/GSK3β/mTOR axis [45], while the ginsenosides Rb3, Rc, Rh2 and F2 from *Panax ginseng* [42] and *Panax quinquefolius* [43] bound HSP90AA1, SRC and AKT1 in lung cancer. Sesquiterpene-rich essential oils represent a distinct subclass that recurred across all four cancer types: the vetiver oil of *Chrysopogon zizanioides*, enriched in isovalencenol, α-vetivol, khusimol, vetiselinenol and α-vetivone [59]; the oils of *Phoebe zhennan*, rich in humulene, eudesmols and elemol [135]; *Cinnamomum tamala* dominated by cinnamaldehyde and δ-cadinene [58]; and *Melaleuca quinquenervia*, rich in 1,8-cineole, α-pinene and viridiflorol [60] consistently engaged AKT1, STAT3 and caspase-3. Diterpenes such as andrographolide identified in *Aspilia pluriseta* [140] and *Euphorbia ingens* [142], the abietane diterpene 16-hydroxy-7α-acetoxyroyleanone isolated from *Coleus amboinicus* active against DU-145 prostate cancer cells [81], and the tigliane diterpene 4 from *Homalanthus giganteus* that was equipotent against doxorubicin-sensitive and doxorubicin-resistant colorectal cancer cells [124] extended the chemical diversity of this class, while the structurally novel tigliane diterpene Dentatacid A from *Euphorbia dentata* [130] further enriched the chemical repertoire.

### 6.4. Polyphenols

Beyond flavonoids, additional polyphenolic classes were broadly represented. Stilbenoids, including resveratrol and pterostilbene identified in *Dracaena cochinchinensis* [72], modulate the PI3K/AKT and oestrogen signalling axes in breast cancer through HDAC inhibition and oestrogen-receptor stabilisation [25]. Curcumin derived from *Curcuma* sp. and used in combination with *Astragalus mongholicus* in a colorectal cancer investigation [115] contributed to HIF-1α inhibition and to tumour-vessel normalisation in vivo. Phenolic acids included rosmarinic acid identified in *Perilla frutescens* [144], *Salvia rosmarinus* [143], *Helicteres isora* [121] and *Paracaryum hedgei* [118]; the rosmarinic acid in the *Helicteres isora* study achieved a docking energy against MAPK1 that exceeded that of the reference BRAF inhibitor encorafenib [121]. Salvianolic acid B identified in *Paracaryum hedgei* [118], and the dietary phenolic acids caffeic acid, ferulic acid, gallic acid and ellagic acid, recurred across multiple studies. The diterpenoid carnosol together with the closely related carnosic acid identified in *Salvia rosmarinus* [143] targeted EGFR, ERBB2 and TP53 in prostate cancer. Anthraquinones, including alizarin, anthragallol and xanthopurpurin from *Rubia tinctorum* [78], bound PLCG1 and BCL2 in breast cancer, while anthocyanins and betalains, including cyanidin 3-glucoside, delphinidin 3,5-diglucoside, betanin, betalains and betaxanthin from *Punica granatum* [100] and *Beta vulgaris* [101], showed both intrinsic cytotoxic activity in breast and colorectal cancer and the additional capacity to act as natural radiosensitisers that enhanced the cytotoxicity of low-dose X-irradiation [100]. Lignans such as sesamin identified in *Piper nigrum* [99] and medioresinol in *Eucommia ulmoides* [127] engaged STAT3, NF-κB and HIF-1α, completing the chemically broad spectrum of polyphenolic constituents documented in this corpus.

### 6.5. Other Bioactive Compounds

A fifth, heterogeneous group of bioactive compounds and formulations also emerged from the review. Plant polysaccharides, including *Astragalus* polysaccharides identified in *Astragalus mongholicus* [63] and *Lycium barbarum* polysaccharide, were investigated as multitarget agents; *Lycium barbarum* polysaccharide, in particular, reversed acquired oxaliplatin resistance in HCT-116-OXR colorectal cancer cells by downregulating phosphomannose isomerase and the ABCG2 transporter [112]. Plant-derived exosome-like nanoparticles isolated from *Polygonatum sibiricum* (approximately one hundred and forty nanometres in diameter and containing eighteen distinct proteins together with several hundred small-molecule metabolites) [65] and phytosome formulations of *Camellia sinensis* polyphenols [69] represent emerging nanovesicular delivery strategies that intersect naturally with network pharmacology by accommodating the multicomponent nature of plant extracts. Iridoid glycosides such as agnuside from *Vitex trifolia* [138] and the prodrug 8-O-acetylharpagide from *Ajuga decumbens* [74], which generated two characterised active metabolites in vivo, provide a unique mechanistic angle that emphasises the role of pharmacokinetics and bioactivation in shaping the spectrum of detectable network-pharmacology hits. Steroidal alkaloids including solasodine and peimine identified in *Impatiens balsamina* [139] and *Fritillaria cirrhosa* [64], the carotenoid β-carotene identified in *Basella alba* [82], the tigliane diterpene Dentatacid A with an unprecedented 2,3-seco-arbor-2,3-dioic skeleton from *Euphorbia dentata* [130], and complex multicomponent essential-oil mixtures in which the bioactivity cannot be attributed to a single dominant constituent, further illustrate the chemical breadth of the corpus and the methodological flexibility of the network-pharmacology workflow.

## 7. Integration of In Silico Predictions with Experimental Validation

### 7.1. Concordance Between Network-Pharmacology (NP) Predictions and In Vitro Findings

Across the studies reviewed here, the overall concordance between in silico predictions and subsequent in vitro experimental findings was high. In studies that screened plant extracts by network pharmacology and then tested the most enriched hub-gene predictions, dose-dependent reductions in cell viability, induction of apoptosis, suppression of migration and modulation of the predicted hub-gene transcripts or proteins were observed in the predicted direction in most cases. Western-blot or quantitative reverse-transcription PCR validation typically confirmed downregulation of the catalytic and regulatory subunits of PI3K, of AKT phosphorylation, of BCL2 and of ERK1/2 phosphorylation, and upregulation of TP53, BAX, cleaved caspase-3 and PARP, which is in line with PI3K/AKT/mTOR and apoptosis-pathway enrichment. This pattern was exemplified by the *Anemarrhena asphodeloides* study [38], in which the predicted AKT1, SRC and HSP90AA1 modulation was confirmed at the transcript level by qRT-PCR; by the *Sophora alopecuroides* chloroform fraction [71], in which the AKT1, TNF and CDK2 targets predicted by docking were validated by their corresponding cellular effects; by the *Lycium barbarum* polysaccharide study [112], in which the predicted PI3K, ABCG2 and phosphomannose isomerase modulation was confirmed at the protein level; and by the *Impatiens balsamina* semen extract [139], in which the predicted androgen-receptor downregulation through ATF3 was confirmed by Western blot. Selectivity indices comparing tumour cells with non-tumourigenic controls were reported in most studies, with values ranging from modest (selectivity indices in the range of two to four) to outstanding, such as the value above twenty-seven reported for methyl cis-p-coumarate from *Pisum sativum* on MCF-7 cells [83] and the selectivity index of approximately four for the abietane diterpene 16-hydroxy-7α-acetoxyroyleanone from *Coleus amboinicus* on DU-145 prostate cancer cells [81]. Several investigations directly benchmarked their lead compounds against clinically used reference inhibitors, including the rosmarinic acid versus encorafenib MAPK1 comparison performed in the *Helicteres isora* study [121], the catechin versus Stattic STAT3 comparison performed in the *Rubus ulmifolius* study [119], the amorphigenin versus geldanamycin HSP90AA1 comparison in the *Elaeagnus caudata* study [54] and the kaempferol-3-O-glucopyranoside versus reference PI3K inhibitor comparison in the *Gladiolus italicus* study [131]. Discrepancies between predicted and observed mechanisms were rare and most often reflected pathway redundancy rather than an outright failure of the network-pharmacology approach, although a small number of studies reported targets that were predicted in silico but not detectably modulated in vitro, underscoring the value of independent experimental confirmation [8].

### 7.2. In Vivo Validation Studies

**Figure 7:**
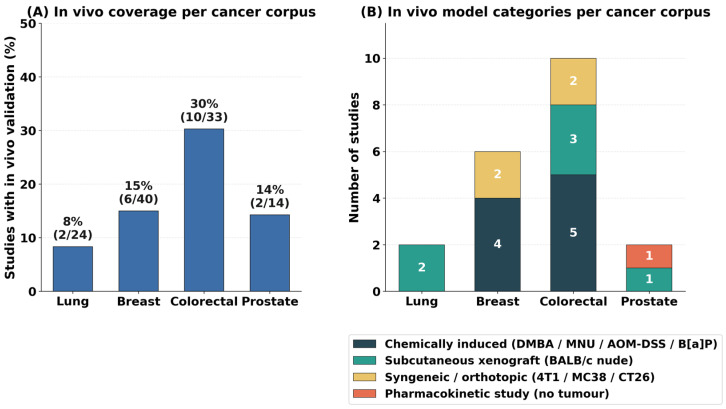
Distribution of in vivo validation studies across the four reviewed cancer types. (**A**) Percentage of studies per cancer corpus that reported any in vivo experiment, expressed as a fraction of the cancer-specific corpus. (**B**) Stacked bar chart showing the number of studies per cancer corpus that used each principal in vivo model category, including chemically induced carcinogenesis (DMBA, MNU, AOM/DSS, benzo[a]pyrene), subcutaneous xenografts in immunodeficient mice, syngeneic or orthotopic transplantation models, embryonic zebrafish xenografts and pharmacokinetic studies without an established tumour model.

Despite the high concordance between in silico predictions and in vitro readouts, in vivo validation remained an inconsistent feature of the reviewed network-pharmacology literature. A minority of the studies reported animal experiments, with marked between-cancer differences: the colorectal cancer corpus had by far the most extensive in vivo coverage, the breast cancer corpus had an intermediate proportion, the lung cancer corpus had a limited number of animal studies, and the prostate cancer corpus only had one true xenograft validation. These proportions and the underlying distribution of in vivo model categories are summarised in Figure 7. The animal models employed included chemically induced colorectal carcinogenesis using the AOM/DSS-induced colitis-associated carcinoma model in Kunming and C57BL/6 mice (*Paeonia lactiflora* [109], *Sanguisorba officinalis* [110], *Gymnanthemum amygdalinum* [120] and the *Glycyrrhiza* species liquorice extract [132]) and the benzo[a]pyrene-induced colon damage model in rats (*Saurauia vulcani* fermented as kombucha [126]), as well as chemically induced mammary carcinogenesis using the DMBA-induced model in rats and mice (*Nigella sativa* [96], *Caesalpinia pulcherrima* [77], *Rauvolfia tetraphylla* [93] and *Loranthus micranthus* [90]) and the MNU-induced rat mammary carcinoma model. A second large group of investigations used transplanted tumour models, including: syngeneic 4T1 mammary tumours in BALB/c mice (*Ajuga decumbens* [74] and the *Allium sativum* and *Zingiber officinale* combination [97]); subcutaneous xenografts in BALB/c nude mice using A549 cells (*Coreopsis tinctoria* [52]; *Achyrocline satureioides* [49]), HCT-116 or the oxaliplatin-resistant HCT-116-OXR (*Lycium barbarum* polysaccharide [112]), SW480 and SW620 cells (*Rhus chinensis* [129]; *Glycyrrhiza uralensis* [114]) and PC-3 cells (*Impatiens balsamina* [139]); orthotopic CT26-luciferase implantation in BALB/c mice (*Astragalus mongholicus* and Curcuma combination [115]); syngeneic MC38 implantation in C57BL/6 mice (*Biancaea sappan* [122]); and an embryonic zebrafish HCT-116 xenograft model used in parallel with rodent experiments by the *Glycyrrhiza* species liquorice extract group [132]. This last investigation, which simultaneously validated efficacy in two complementary in vivo systems and confirmed RAS-pathway dependence through the rescue experiment with the RAS activator ML-099 [132], represents one of the most methodologically sophisticated in vivo validations in the present review and illustrates the increasingly multi-system approach to translational confirmation of network-pharmacology predictions.

### 7.3. Clinical Relevance and Translational Potential

Direct clinical evidence from prospective trials was absent from the reviewed network-pharmacology corpus, which reflects the design of these studies as integrated computational and experimental analyses rather than clinical investigations. Indirect clinical relevance, however, was supported through several complementary strategies. Several studies used publicly available patient cohorts, including The Cancer Genome Atlas (TCGA), UALCAN and the Gene Expression Omnibus (GEO), to confirm that predicted hub genes are dysregulated in patient tumours and to relate their expression to overall survival. This strategy was exemplified by the survival analyses of ERBB2 and MAPK1 in *Helicteres isora*-treated colorectal cancer [121], of HSP90AA1 in *Elaeagnus caudata*-treated colorectal cancer [54], and of CAV1, FGF2 and PPARG in the *Polygonatum sibiricum* exosome-like nanoparticle study [65]. Other investigations linked their network-pharmacology findings to clinical practice through the use of clinically used polyherbal formulations, including the eighteen-herb Chinese Herbal Medicine formula associated with a reported survival benefit in stage IV lung adenocarcinoma [47]. A second translational angle was provided by combination strategies aimed at reversing acquired resistance to standard chemotherapy: the *Sophora alopecuroides* chloroform fraction combined with adriamycin in MCF-7/ADR cells [71], *Lycium barbarum* polysaccharide combined with oxaliplatin in HCT-116-OXR cells [112], *Kalanchoe laciniata* flavonoid glycosides combined with doxorubicin in triple-negative breast cancer [86], and the *Punica granatum* and *Beta vulgaris* cyanidin- and betalain-rich combination as a natural radiosensitiser that enhanced the cytotoxicity of low-dose X-irradiation against MDA-MB-231 cells [100,101]. Drug-likeness assessment using Lipinski’s rule of five, pkCSM absorption–distribution–metabolism–excretion–toxicity (ADMET) prediction and oral bioavailability calculations were routinely performed and generally supported the developability of the lead phytochemicals; however, well-documented bioavailability and pharmacokinetic limitations of curcumin [115], epigallocatechin-3-gallate [69] and resveratrol [72], which involve low aqueous solubility, extensive first-pass metabolism and rapid glucuronidation, remain a recognised translational obstacle that the reviewed publications acknowledge but rarely resolve experimentally [7,29]. It should be emphasised that this absence refers to the reviewed studies themselves; several of the leading compounds have nonetheless entered registered clinical trials conducted independently of this network-pharmacology literature.

### 7.4. Registered Clinical Trials of the Leading Plant-Derived Compounds

To complement the review-internal appraisal above and to identify more precisely which agents are closest to the clinic, we surveyed the ClinicalTrials.gov registry for interventional oncology trials of the phytochemicals that recurred most frequently as active constituents across the studies reviewed here. It should be emphasised that these registered trials lie outside the network-pharmacology corpus that forms the basis of this review and were not retrieved through the systematic literature search of this review; they are presented solely to contextualise the translational maturity of the leading compounds. The survey shows that the dominant chemical classes of this review, namely curcuminoids (curcumin), stilbenoids (resveratrol), catechins (epigallocatechin-3-gallate, as green tea extract), isoflavones and flavonols (genistein, quercetin) isothiocyanates (sulforaphane) and alkaloids (berberine), together with the flavonolignan silibinin, have already entered predominantly phases I and II clinical evaluation in the four cancers considered here, most often as chemopreventive agents, as pre-surgical (window-of-opportunity) interventions, or as adjuncts to approved chemotherapy. Representative registered trials are summarised in Table 2.

Curcumin is the most extensively registered of these agents, with phase II trials spanning colorectal chemoprevention (NCT00365209), combination with FOLFOX in metastatic colorectal cancer (CUFOX; NCT01490996), adjunctive use during radiotherapy in breast cancer (NCT01740323) and combination with docetaxel in metastatic castration-resistant prostate cancer (NCT02095717). Green tea catechins and the isoflavone genistein have progressed furthest in prostate and breast cancer, including active-surveillance and pre-prostatectomy prostate cancer trials (NCT04300855, NCT01340599, and NCT00596895) and breast cancer prevention and stage IV combination trials (NCT00290758, NCT00244933). Sulforaphane has been evaluated in biochemically recurrent prostate cancer (NCT01228084) and in lung cancer chemoprevention in former smokers (NCT03232138), whereas silibinin is being tested peri-operatively in colorectal cancer (NCT01239095) and, most recently, for the prevention of intracranial recurrence of non-small-cell lung and breast cancer brain metastases (SILMET; NCT05689619). The alkaloid berberine has additionally been evaluated in a large randomised trial for the prevention of colorectal adenoma recurrence after polypectomy (NCT02226185), and green tea catechins have entered a phase I trial in hormone-receptor-negative breast cancer (NCT00516243). Two themes recur across these trials and mirror the conclusions of the present review: the compounds are consistently reported to be safe and well tolerated, and their principal limitation remains pharmacokinetic rather than pharmacodynamic, which is why several of the more recent trials employ improved-bioavailability formulations, such as the phospholipid-complexed curcumin used in NCT01740323 and the defined green tea catechin extracts used in NCT04300855. Importantly, apart from a completed phase II/III berberine trial for the prevention of colorectal adenoma recurrence (NCT02226185), which is a chemoprevention rather than a treatment study, no phase III efficacy trial has yet been completed for these agents in patients with established lung, breast, colorectal or prostate cancer, underscoring that, although the leading plant-derived compounds have clearly entered clinical development, definitive evidence of clinical benefit is still awaited.

## 8. Challenges and Limitations

The reviewed literature, while collectively demonstrating the value of network pharmacology for plant-derived anticancer drug discovery, also exposes several methodological and translational limitations that constrain the field. First, the underlying databases are themselves biased: TCMSP is heavily focused on traditional Chinese medicine and underrepresents non-Asian medicinal flora, the default oral bioavailability and drug-likeness thresholds (OB ≥ 30%, DL ≥ 0.18) may exclude bioactive compounds that act locally or after intestinal metabolism, and target-prediction tools such as SwissTargetPrediction or SEA are limited by the chemical space of their training sets and produce a non-negligible rate of false positives. Second, the experimental validation in this corpus is uneven. Although every included study fulfilled the inclusion requirement of in vitro or in vivo confirmation, the proportion of studies with in vivo evidence varies dramatically across the four cancer types, with colorectal cancer being notably better represented than the other three, and the most frequent cell line for each cancer (A549 for lung, MCF-7 for breast, HCT-116 for colorectal, and DU-145 for prostate) covers only a fraction of the molecular heterogeneity of the disease, leaving subtypes such as SCLC, HER2-enriched breast cancer, MSI-high colorectal cancer or neuroendocrine prostate cancer poorly represented. Third, most studies employed crude extracts or partially purified fractions of variable, often unstandardised, phytochemical composition, which complicates the attribution of activity to specific compounds, the reproducibility across laboratories and the comparison with the network-pharmacology predictions. Fourth, only a minority of investigations benchmarked their lead compounds against clinically used reference inhibitors (notable exceptions include the rosmarinic acid versus encorafenib MAPK1 comparison in *Helicteres isora* [121] and the catechin versus Stattic STAT3 comparison in *Rubus ulmifolius* [119]), so the comparative therapeutic value of plant compounds against approved drugs is rarely established. Fifth, bioavailability, pharmacokinetic and ADMET considerations remain insufficiently addressed: well-documented issues with curcumin, epigallocatechin-3-gallate (EGCG), resveratrol and many alkaloid glycosides (low aqueous solubility, first-pass metabolism, and rapid glucuronidation) are acknowledged but rarely overcome experimentally. Sixth, prospective clinical evidence is absent from the included network-pharmacology corpus, and although several studies use TCGA-based survival analysis to suggest translational relevance, such associative analyses cannot replace controlled clinical trials; several of the leading compounds have, however, already entered registered clinical trials conducted independently of this literature. Finally, the reviewed studies varied considerably in the depth of their network-pharmacology analysis, ranging from complete pipelines encompassing compound-target network construction, topological identification of hub nodes and module detection to more limited approaches that combined target prediction, pathway-enrichment analysis and molecular docking; both categories were retained, provided that experimental validation was reported, but this heterogeneity, together with differences in the transparency of methodological reporting (compound libraries, screening thresholds and validation depth), complicates direct comparison of their network-level conclusions and is reflected in the study-by-study appraisal provided for each cancer type.

## 9. Future Perspectives

Several methodological and strategic directions are likely to shape the next generation of network-pharmacology studies on plant-derived anticancer agents. Integration of multi-omics datasets, including bulk and single-cell transcriptomics, proteomics, lipidomics and metabolomics, into the network-pharmacology pipeline is expected to refine target prediction by linking compounds not only to genes but also to context-specific cellular states, exemplified in this review by the MALDI mass-spectrometry imaging analysis of *Achyrocline satureioides*-treated A549 xenografts [49] and the cell metabolomics analysis of the same extract. The deployment of artificial intelligence and graph neural networks for compound and target prediction, deep-learning-based virtual screening and large language models for literature mining is expected to accelerate the curation of plant chemical databases and to extend coverage beyond the heavily TCM-focused TCMSP, with several recent reviews already proposing AI-enabled multi-omics network-pharmacology workflows. Patient-derived organoids and microfluidic tumour-on-chip platforms offer an opportunity to bridge the gap between cell-line monocultures and animal xenografts and to test plant compounds in genetically defined and microenvironmentally controlled systems. Nanocarrier-based and exosome-based delivery, of which the *Polygonatum sibiricum*-derived exosome-like nanoparticles [65] and the *Camellia sinensis* phytosome formulation [69] are early proofs of concept in the present corpus, can directly address the ADMET limitations of curcumin, resveratrol, EGCG and other low-bioavailability phytochemicals. Combination strategies with approved cytotoxic chemotherapy (doxorubicin, oxaliplatin, and 5-fluorouracil), targeted therapy (EGFR-TKIs in lung, CDK4/6 and PI3K inhibitors in breast, and anti-androgens in prostate) and immune-checkpoint blockade (anti-PD-1/PD-L1 antibodies and inhibitors of the indoleamine 2,3-dioxygenase (IDO) and tryptophan 2,3-dioxygenase (TDO) enzymes as exemplified by *Loranthus micranthus* [90]) represent the most plausible near-term translational path. Finally, standardisation of the network-pharmacology workflow itself, including transparent reporting of compound libraries, screening thresholds, target prediction parameters, network construction settings and the level of experimental validation, will be essential to enable reproducible meta-analyses and to bring the field closer to the rigour required for regulatory acceptance.

## 10. Conclusions

The present review of 101 network-pharmacology studies of plant-derived compounds in the four globally leading cancers, namely lung, breast, colorectal and prostate, demonstrates that this integrative computational and experimental framework provides a reproducible and biologically meaningful link between phytochemistry and oncology. Across the four malignancies, the analyses consistently converge on a relatively conserved set of master regulators dominated by AKT1, EGFR, TP53, STAT3, MAPK1 or MAPK3, CASP3, HSP90AA1, PIK3CA and BCL2, and on the canonical PI3K/AKT/mTOR, MAPK, apoptosis and p53 axes. Each cancer type, however, retains a distinct molecular signature, captured by the AR axis in prostate cancer, the ESR1, HER2 and oestrogen-signalling pathways in breast cancer, the CTNNB1 and Wnt-pathway nodes together with glycolytic enzymes in colorectal cancer, and the EGFR/RAS/MAPK and epithelial-to-mesenchymal transition effectors in lung cancer. Flavonoids, terpenoids, alkaloids and polyphenols emerged as the dominant chemical classes, but the corpus also includes notable innovations such as plant-derived exosome-like nanoparticles, phytosome formulations, the reversal of multidrug resistance, immune-checkpoint modulation by phytochemicals and the use of fermented plant products. The single most important limitation that emerged from this review is the uneven and often insufficient breadth of in vivo validation, particularly in lung and prostate cancer, and the near-complete absence of prospective clinical validation of the network-pharmacology predictions. Closing these gaps through systematic in vivo validation, patient-derived organoids, multi-omics integration, AI-enabled refinement of in silico predictions and combination strategies with approved targeted and immunotherapy agents will be essential for translating the chemical and mechanistic promise of plant-derived anticancer compounds into rigorous clinical benefit.

## Figures and Tables

**Table 1:** Cross-cancer comparison of network-pharmacology findings across the four reviewed cancer types. The Table summarises the most frequently identified hub genes and enriched signalling pathways, the principal in vitro and in vivo experimental models used and the key validation findings, together with the full set of literature references analysed for each cancer in the present review.

Cancer Type	Top Recurrent Hub Genes	Most Enriched Signalling Pathways	In Vitro Cell Lines Tested	Key In Vitro Findings	In Vivo Organisms/Models	Key In Vivo Findings	References
Lung cancer (NSCLC, LUAD)	AKT1, EGFR, TP53, MAPK1/3, CASP3, STAT3, HSP90AA1, PIK3R1/PIK3CA, BCL2, SRC, mTOR, MMP9	PI3K-Akt, MAPK, Non-small-cell lung cancer, EGFR tyrosine-kinase inhibitor resistance, Apoptosis, TNF and IL-17 signalling	A549 (dominant); H1299, NCI-H460, NCI-H1395, HCC827 in a smaller subset of studies; BEAS-2B, L929, HEK-293, CV-1 and HDFn as selectivity controls	Dose-dependent reduction in cell viability; G0/G1 or G2/M arrest; annexin V/propidium iodide or TUNEL-based apoptosis quantification; transwell-confirmed inhibition of migration; Western blot and qRT-PCR confirmation of AKT1, SRC, HSP90AA1 and EGFR downregulation	A549 xenograft in BALB/c nude mice (*Coreopsis tinctoria*; *Achyrocline satureioides*)	Suppression of tumour growth; MALDI mass-spectrometry imaging revealed disruption of the tricarboxylic acid cycle in vivo (*Achyrocline satureioides*)	[37,38,39,40,41,42,43,44,45,46,47,48,49,50,51,52,53,54,55,56,57,58,59,60]
Breast cancer (Luminal A/B, HER2-enriched, TNBC)	AKT1, EGFR, TP53, ESR1, STAT3, SRC, HSP90AA1, MAPK1/3, HIF1A, CDK2/4/6, PIK3CA, MMP9, CASP3, BCL2, HER2/ERBB2	PI3K-Akt, MAPK, Apoptosis, p53, oestrogen signalling, endocrine resistance, steroid hormone biosynthesis, breast cancer pathway, ErbB, PD-L1/PD-1 checkpoint, Wnt, Hippo (YAP1)	MCF-7 (luminal, dominant); MDA-MB-231 (TNBC, dominant); T47D, MDA-MB-468, 4T1 in additional studies; MCF-10A, HUVEC, L929, Vero, RIN-5F, HEK-293 and CV-1 as selectivity controls	Selective cytotoxicity (IC50 from approximately one to five hundred micrograms per millilitre); G0/G1 or S-phase arrest; reversal of multidrug resistance in MCF-7/ADR; mitochondrial-membrane depolarisation (JC-1); reduction in MMP9, ESR1 and AKT phosphorylation by Western blot	Syngeneic 4T1 mammary tumours in BALB/c mice (*Ajuga decumbens*; *Allium sativum* and *Zingiber officinale*); DMBA-induced mammary carcinogenesis in rats and mice (*Nigella sativa*; *Caesalpinia pulcherrima*; *Rauvolfia tetraphylla*; *Loranthus micranthus*); MNU-induced rat mammary carcinoma	Reduction in tumour volume and weight; restoration of normal mammary histology; reduction in serum tumour markers (AFP, CA-125); downregulation of oestrogen-receptor-α expression in mammary tissue; increase in CD4+ T-cell infiltration with concomitant CTLA-4 reduction (*Loranthus micranthus*)	[51,53,63,64,65,66,67,68,69,70,71,72,73,74,75,76,77,78,79,80,82,83,84,85,86,87,88,89,90,91,92,93,94,95,96,97,98,99,100,102]
Colorectal cancer (sporadic and hereditary, CAC, MSI-H/MSS, CMS1–CMS4)	AKT1, EGFR, TP53, STAT3, PIK3CA, MAPK1/3, CASP3, HSP90AA1, TNF, SRC, BCL2, MMP9, HIF1A, CTNNB1, glycolytic enzymes (ENO1, ALDOA, PFKFB3, PKM2, LDHA)	PI3K-Akt, MAPK, apoptosis, TNF, EGFR tyrosine-kinase inhibitor resistance, HIF-1, p53, cell-cycle, colorectal cancer pathway, Wnt and β-catenin signalling, IL-17, NF-κB	HCT-116 (dominant); HT-29 (dominant); SW480 and SW620 in matched primary–metastatic comparisons; LoVo, WiDr, COLO 320DM, COLO 205, COLO 320, KM12SM, MC38 and CT26 (the latter two in syngeneic and orthotopic settings); FHC, NCM460, HEK-293, WI-38, L929 and HDFn as selectivity controls	Selective cytotoxicity (IC50 from approximately two to two hundred micrograms per millilitre); reversal of acquired oxaliplatin resistance in HCT-116-OXR; suppression of STAT3 and AKT phosphorylation; caspase-3 and caspase-7-dependent apoptosis; downregulation of MMP2, MMP9 and ABCG2; identification of structurally novel tigliane diterpene (e.g., Dentatacid A)	AOM/DSS-induced colitis-associated carcinoma in Kunming and C57BL/6 mice; benzo[a]pyrene-induced model in rats; subcutaneous xenografts of HCT-116, HCT-116-OXR, SW480 and SW620 in BALB/c nude mice; orthotopic CT26-luciferase implantation in BALB/c mice; syngeneic MC38 in C57BL/6 mice; embryonic zebrafish HCT-116 xenografts (liquorice extract study)	Reduction in tumour number, size and metastatic burden; restoration of mucosal integrity and goblet-cell density; downregulation of EGFR, HIF-1α and the RAS-RAF-MEK-ERK cascade (rescued by the RAS activator ML-099); tumour-vessel normalisation through direct HIF-1α binding; epigenetic hypermethylation of the TNF-α and p65 gene promoters	[50,54,59,84,85,101,106,107,108,109,110,111,112,113,114,115,116,117,118,119,120,121,122,123,124,125,126,127,128,129,130,131,132]
Prostate cancer (acinar adenocarcinoma, CRPC, AR-V7-driven, neuroendocrine variants)	AKT1, EGFR, TP53, ESR1, AR, MAPK1/3, BCL2, PIK3R1, MMP9, MMP2, CASP3, CCND1, CDK2, PDGFRA, PDGFRB, FYN, ATF3, ERBB2	PI3K-Akt, prostate cancer pathway, MAPK, p53, EGFR tyrosine-kinase inhibitor resistance, apoptosis, TNF, NF-κB, endocrine resistance, AMPK, steroid biosynthesis, PD-L1/PD-1 checkpoint	DU-145 (AR-negative castration-resistant, dominant); PC-3 (AR-negative bone-metastatic, dominant); LNCaP (AR-positive); 22Rv1 (CRPC, AR-V7-expressing); Vero E6, PNT2, CV-1 and RAW 264.7 as selectivity controls	IC50 values in the sub-microlitre per millilitre range for sesquiterpene-rich essential oils (*Phoebe zhennan*); G0/G1 or G2/M arrest; downregulation of AR transcriptional activity through ATF3 (*Impatiens balsamina*); selective cytotoxicity towards tumour cells with sparing of the normal prostatic epithelial line PNT2	PC-3 xenograft in BALB/c nude mice (*Impatiens balsamina*); pharmacokinetic profiling in male Sprague–Dawley rats (*Vitex trifolia*)	Reduction in PC-3 tumour growth and androgen-receptor expression in vivo (*Impatiens balsamina*); characterisation of absorbed bioactive metabolites by pharmacokinetic profiling (*Vitex trifolia*)	[51,81,85,98,135,136,137,138,139,140,141,142,143,144]

**Table 2:** Representative registered interventional clinical trials of the leading plant-derived compounds of this review in the four cancers considered, retrieved from ClinicalTrials.gov (accessed on 10 April 2026). These trials are external to the network-pharmacology corpus reviewed here and are provided only to indicate translational maturity. CRPC, castration-resistant prostate cancer; HR, hormone receptor; PSA, prostate-specific antigen.

Compound	Cancer Type	Setting/Intervention	Phase	ClinicalTrials.gov ID
Curcumin	Colorectal	Chemoprevention (aberrant crypt foci)	II	NCT00365209
Curcumin	Colorectal	With FOLFOX, metastatic (CUFOX)	II	NCT01490996
Curcumin	Breast	Adjunct to radiotherapy, post-chemotherapy	II	NCT01740323
Curcumin	Prostate	With docetaxel, metastatic CRPC	II	NCT02095717
Resveratrol	Colorectal	Pre-surgical, resectable disease	I	NCT00433576
Resveratrol	Breast	Prevention (postmenopausal, biomarker)	I	NCT01370889
Green tea catechins (EGCG)	Prostate	Pre-prostatectomy (Polyphenon E)	II	NCT01340599
Green tea catechins (EGCG)	Prostate	Active surveillance	II	NCT04300855
Green tea catechins (EGCG)	Breast	Post-treatment, HR-negative disease	I	NCT00516243
Quercetin (with green tea)	Prostate	With docetaxel, CRPC	I/II	NCT06615752
Genistein	Prostate	PSA-recurrent disease	II	NCT00596895
Genistein	Breast	Prevention, high-risk women	II	NCT00290758
Genistein	Breast	With gemcitabine, stage IV	II	NCT00244933
Sulforaphane	Prostate	Biochemically recurrent disease	II	NCT01228084
Sulforaphane	Lung	Chemoprevention, former smokers	II	NCT03232138
Silibinin (with green tea)	Colorectal	Peri-operative (resection)	I	NCT01239095
Silibinin	Lung/Breast	Brain-metastasis prevention (SILMET)	II	NCT05689619
Berberine	Colorectal	Prevention of adenoma recurrence	II/III	NCT02226185

## Data Availability

All data supporting the findings of this study are available within the article and its Supplementary Materials.

## Supplementary Materials

The following supporting information can be downloaded at: https://www.mdpi.com/article/10.3390/ijms27146177/s1.
