# Supplementary material for: A Network Pharmacology Review of Plant-Derived Anticancer Compounds in Lung, Breast, Colorectal and Prostate Cancer
Source: Int J Mol Sci. 2026 Jul 10;27(14):6177. doi: 10.3390/ijms27146177 (PMC13411852; doi:10.3390/ijms27146177)
Supplement: Supplementary file 1 [file ijms-27-06177-s001.zip › ijms-4412259-supplementary.pdf]

Supplementary Materials

This file contains the four full data tables (Tables S1–S4) summarising the 101 network-pharmacology studies of plant-derived compounds in lung, breast, colorectal and prostate cancer that are analysed in the main manuscript "Network Pharmacology in the Study of Plant-Derived Anticancer Compounds in the Four Most Frequent Cancers Worldwide: Lung, Breast, Colorectal and Prostate". Each table reports, for each study, the cancer type, plant species and part, principal compounds, top hub genes predicted by network pharmacology, enriched signalling pathways, in vitro and in vivo experimental models and the principal findings reported by the original authors.

Table S1. Network Pharmacology-Based Studies on Plant-Derived Compounds in Lung Cancer.

Representative Plant Species/Formula/ Compound / /Plant part	Major Compound Classes	Top 5 Hub Genes	Enriched Pathways	In vitro Models	In vivo Models	Clinical Relevance	Ref.
Stemona tuberosa Lour. (Roots)	4-Azatricyclo undecan-5-one (ADE), Dihydro-normorphine, 3-desoxy- (DNY), N-(2,3-Epoxypropyl)-1,2,3,4,9,10-Hexahydroacridin-9-One (EHE)	SRC, EGFR, JUN, PPARG, MTOR	SRC Inhibition, Apoptosis Induction	Human lung adenocarcinoma cell line (A549)	None	• Methanolic extract of *S. tuberosa* showed significant cytotoxicity, suppressed colony formation, and induced apoptosis in A549 lung cancer cells<br>• The extract's activity is linked to increased DNA damage, elevated lipid peroxidation (ROS), and upregulation of pro-apoptotic genes<br>• Network pharmacology identified SRC, a crucial kinase in lung cancer proliferation and metastasis, as the primary molecular target<br>• Compounds ADE and DNY showed notable binding affinity to SRC, and were identified as effective SRC inhibitors, suggesting S. tuberosa is a promising candidate for cancer therapy	[37]
Anemarrhena asphodeloides Bunge (Rhizome)	Kaempferol, Asperglaucide, Coumaroyltyramine, Mangiferolic acid, Diosgenin	AKT1, SRC, HSP90AA1, STAT3, EGFR	PI3K-Akt signaling pathway, Pathways in cancer, MAPK signaling pathway, Ras signaling pathway, Rap1 signaling pathway	Human NSCLC cells (A549); BEAS-2B normal lung cells (selectivity)	None	• TCMSP screening identified 15 bioactive compounds (OB≥30%, DL≥0.18) targeting 309 NSCLC-related genes<br>• Kaempferol, asperglaucide, and coumaroyltyramine showed strongest binding to AKT1, SRC and HSP90AA1; 100 ns MD simulations confirmed stable complexes<br>• All three compounds dose-dependently reduced A549 viability without BEAS-2B toxicity, inhibited migration/invasion (transwell) and induced apoptosis (TUNEL)	[38]

						• qRT-PCR confirmed downregulation of AKT1, SRC, and HSP90AA1 mRNA in treated A549 cells	
Astragalus mongholicus Bunge (Root)	Astragaloside A	STAT3, AKT1, HSP90AA1, MTOR, NFKB1, ESR1	PI3K-Akt signaling pathway, HIF-1 signaling pathway, Proteoglycans in cancer, PD-L1 expression and PD-1 checkpoint pathway in cancer	Human non-small lung cancer cell line (A549)	None	• Astragaloside A (AS-A) regulates key signaling pathways in lung adenocarcinoma<br>• The mechanism of AS-A involves targeting hub genes such as STAT3 and AKT1 and modulating the PI3K-Akt and HIF-1 signaling pathways<br>• Molecular docking and dynamics simulations confirmed the stable and strong binding of AS-A to key protein targets, particularly PIK3R1<br>• In vitro experiments showed that AS-A treatment could reverse the dysregulation of key signaling molecules in a lung adenocarcinoma model	[39]
Glycyrrhiza glabra L. (Roots)	18α-glycyrrhetinic acid (18α-GA)	EGFR, AKT1, PIK3R1, MAPK1, IGF1, SRC	PI3K-Akt signaling pathway, ErbB signaling pathway, Pathways in cancer, Ras signaling pathway, Proteoglycans in cancer	Human non-small cell lung cancer cell lines (H1299 and A549)	None	• 18α-GA inhibits proliferation, arrests the cell cycle at the G1 phase, and induces apoptosis in NSCLC cells<br>• It decreases the migratory potential of cancer cells<br>• The anti-cancer mechanism involves the inhibition of the EGFR-PI3K/AKT signaling pathway	[40]
Lonicera japonica Thunb. (Flower)	Quercetin, Apigenin, Luteolin, Ursolic acid, Kaempferol	PECAM1, SPP1	IL-17 signaling pathway, TNF signaling pathway, mTOR signaling pathway, B cell receptor	Human non-small cell lung cancer (A549) cells	None	• Lonicera japonica flos (LJF) inhibits the proliferation, migration, and invasion of A549 lung cancer cells<br>• PECAM1 and SPP1 were identified as the key therapeutic targets using network pharmacology and machine learning algorithms	[41]

			signaling pathway, Cell cycle			• The mechanism is associated with the regulation of pathways involved in cell cycle, immune response (TNF, IL-17), and mTOR signaling<br>• LJF treatment significantly upregulated the protein expression of PECAM1 and SPP1 in A549 cells	
Panax ginseng C.A.Mey. (Leaves)	Sitosterol, Kaempferol, Quercetin, Ginsenoside rh2, Ginsenoside-F2	JUN, STAT3, AKT1, TNF, MAPK1, TP53	PI3K-Akt signaling pathway, MAPK signaling pathway, TNF signaling pathway	Human lung adenocarcinoma cell line (A549)	None	• Ginseng leaf extract inhibits the proliferation of lung adenocarcinoma cells<br>• The immunoregulatory and anti-cancer mechanism is linked to the PI3K-Akt, MAPK, and TNF signaling pathways<br>• Active compounds like Ginsenoside F2 and Kaempferol showed strong binding affinity to core targets such as STAT3 and AKT1	[42]
Panax quinquefolius L. (Root)	Ginsenoside Rb3, Notoginsenoside R1, Ginsenoside Rc	HSP90AA1, SRC, PI3K	PI3K-Akt signaling pathway, Ras signaling pathway, MAPK signaling pathway	Human lung adenocarcinoma cell line (A549)	None	• Ginsenoside Rb3, notoginsenoside R1, and ginsenoside Rc are the most abundant biomarkers in P. quinquefolius from the Wendeng region<br>• These compounds bind to Hsp90α, leading to the inactivation of SRC and PI3K kinase<br>• This interaction ultimately results in the inactivation of the Akt and ERK pathways, leading to the suppression of lung cancer cells	[43]
Pinellia ternata (Thunb.) Makino (Tuber)	β-sitosterol, Stigmasterol, Baicalein, Cavidine, Poriferast-5-en-3beta-ol	AKT1, PI3K, MMP9, HIF-1α, TGF-β, BCL2	Purine metabolism, Riboflavin metabolism, Pentose phosphate pathway	Human non-small cell lung cancer lines (A549, NCI-H460), Human normal lung cell line (BEAS-2B)	None	• *Pinellia ternata* extract inhibits the proliferation, migration, and invasion of non-small cell lung cancer cells<br>• The anti-cancer mechanism is linked to the modulation of purine metabolism, riboflavin metabolism, and the pentose phosphate pathway<br>• The extract was found to significantly	[44]

						inhibit the expression of proteins in the PI3K/AKT signaling pathway	
Dioscorea zingiberensis C.H.Wright (Rhizome)	Dioscin	AKT1, mTOR, TP53, CASP3, TNF	AKT/GSK3b/mTOR signaling, PI3K-Akt signaling	Human lung adenocarcinoma cell lines (A549 and H1299)	None	• Dioscin suppresses proliferation, migration, and invasion of lung adenocarcinoma cells<br>• It reverses epithelial-mesenchymal transition (EMT)<br>• The anti-tumor mechanism occurs via the inactivation of the AKT/GSK3b/mTOR signaling pathway, likely by binding to AKT and mTOR and inhibiting their phosphorylation	[45]
Scleromitrion diffusum (Willd.) R.J.Wang (Roots)	2-Methoxy-3-methyl-9,10-anthraquinone, Stigmasterol, β-Sitosterol, Quercetin, Poriferasterol	EGFR, VEGFR, CASP3, CASP9, AKT1	PI3K-Akt signaling pathway, MAPK signaling pathway, Proteoglycans in cancer, MicroRNAs in cancer	Human lung cancer cell lines (A549, HCC827, NCI-H1395)	None	• Network pharmacology identified 88 common targets and 5 active compounds linking SDW to lung cancer • Ethanolic root extract dose-dependently suppressed proliferation of all three NSCLC lines (cell viability inhibition <50% at <2000 μg/mL) • Western blot confirmed strong downregulation of p-Akt and p-Erk1/2, with mild reduction in caspase-9, p-PI3K and EGFR; total Akt, Erk1/2, PI3K and VEGFA unchanged • Molecular docking confirmed good binding affinity of all 5 compounds to core lung cancer proteins	[46]
Chinese Herbal Medicine (18-herb clinical formula) / (Various parts)	Stigmasterol, β-sitosterol, Spinasterol, Quercetin, Baicalein	DRD2, HTR2 (5HTR2), CDK2, FNTA, FNTB	Pathways in cancer, Signal transduction, ErbB signaling pathway, Ras signaling pathway, PI3K-Akt signaling pathway	Human lung carcinoma cell line (A549)	None	• Treatment with a Chinese Herbal Medicine (CHM) formula was associated with a significant survival benefit for patients with stage IV lung adenocarcinoma<br>• 18 herbs were identified as being beneficial to survival<br>• The anti-cancer mechanism is linked to inhibiting growth factor receptors (e.g., EGFR), angiogenesis (e.g., VEGFR, PDGFR), and the Ras signaling pathway	[47]

						• Major sterol compounds (β-sitosterol, stigmasterol, etc.) inhibited lung cancer cell proliferation and key signaling proteins (p-EGFR, p-Akt, p-Erk)	
Melissa officinalis L. (Leaves)	Linoleic acid, Palmitoleic acid, Oleic acid, Palmitic acid, Linolenic acid	ALOX5, PTGES, CYP2C19	Pathways in cancer, Non-small cell lung cancer, Small cell lung cancer, Arachidonic acid metabolism, PPAR signaling pathway	Human lung cancer (A549) and human bronchial epithelial (BEAS-2B) cells	None	• Aqueous extract of M. officinalis inhibits proliferation of lung cancer (A549) and bronchial epithelial (BEAS-2B) cells<br>• The anticancer mechanism is linked to the modulation of cancer-associated pathways, particularly those for lung cancer<br>• Molecular docking showed high binding affinities of linolenic acid, oleic acid, and stearic acid to key protein targets (ALOX5, CYP2C19, and PTGES), indicating a promising strategy for treating lung cancer	[48]
Achyrocline satureioides (Lam.) DC. (Inflorescences, stems, leaves; methanol extract – ASM)	Quercetin, Isorhamnetin, Luteolin, Skrofulein, Hispidulin	HSP90AA1, AKT1, EGFR, STAT3, BCL2	TCA cycle, Histidine metabolism, Pantothenate and CoA biosynthesis, Alanine/aspartate/glutamate metabolism, Glycerophospholipid metabolism	Human NSCLC cells (A549)	A549 xenograft in mice (quercetin treatment)	• HR-UHPLC-MS/MS identified 69 compounds; network pharmacology screened 6 core components and 10 hub targets • ASM inhibited A549 viability (IC_{50} = 133.6 μg/mL) and migration; cell metabolomics revealed 32 altered metabolites • Quercetin suppressed tumor growth in xenograft mice; MALDI-MSI showed 51 altered tumor metabolites with TCA cycle disruption (↓malate, aspartate, serotonin; ↑UDP-glucose, lipids)	[49]
Nandina domestica Thunb. (Fruits)	Isoquercitrin, Quercitrin, Berberine, Chlorogenic acid, Caffeic acid	AKT1, CASP3, MAPK1, TP53	Colorectal cancer, Endometrial cancer, Small-cell lung cancer, Apoptosis, PI3K-Akt signaling	Human colorectal carcinoma (HCT-116)	None	• Identified compounds show synergistic interactions with cancer-related targets, primarily affecting apoptosis and Akt-signaling pathways<br>• The ethyl acetate extract, rich in these compounds, showed the highest cytotoxic activity against HCT-116 colorectal cancer cells	[50]

			pathway			• The mechanism of action is proposed to vary with cancer stages, involving CASP3 and MAPK1 in early stages, and p53 and ErbB signaling in later stages	
Cissus trifoliata (L.) L. (Leaves)	Presqualene diphosphate, Phytol, Stearic acid, Ursolic acid, γ-Linolenic acid	CASP3, PARP1, BAX, BCL2, STAT3	Sesquiterpenoid and triterpenoid biosynthesis, Diterpenoid biosynthesis, Glycerophospho lipid metabolism, Steroid biosynthesis	Human cancer cell lines: Hepatocellular carcinoma (HepG2, Hep3B), Cervical (HeLa), Prostate (PC3), Lung (A549), Breast (MCF7)	None	• Hexane and chloroform/methanol extracts showed cytotoxic activity against various cancer cell lines, especially hepatocellular carcinoma (Hep3B and HepG2)<br>• The sesquiterpenoid and triterpenoid biosynthesis pathway was identified as the most significant<br>• Network pharmacology analysis revealed that compounds like phytol, ursolic acid, and stearic acid target genes involved in apoptosis (e.g., CASP3, BAX, BCL2)	[51]
Coreopsis tinctoria Nutt. (Flower)	(-)-Epigallocatechin gallate, Nobiletin, Quercetin, Luteolin, Cianidanol (Catechin)	Akt1, MAPK1, TP53, Bcl-2, Caspase -3	PI3K-Akt signaling pathway, Pathways in cancer, Apoptosis, Non-small cell lung cancer, Small cell lung cancer	Human lung cancer cell lines (A549, H292)	Nude mice xenograft model (A549 cells)	• Total flavonoids from C. tinctoria inhibit proliferation and induce apoptosis in lung cancer cells in vitro and suppress tumor growth in vivo<br>• The antitumor mechanism is primarily linked to the inhibition of the PI3K-Akt signaling pathway<br>• The flavonoids trigger the mitochondrial-mediated apoptosis pathway, regulating the expression of Bax, Bcl-2, and Caspase-3	[52]
Coleus amboinicus Lour. (Leaves)	2,3-Pyrazinediamine N3-cyclopropyl-N2,N2-dimethyl-5,6-diphenyl, Dehydroabietic acid, 1,4,10,13-Tetraazacyclooctadeca-4,9,13,18-tetraene-7,16-dione 5,9,14,18-tetramethyl, 4-[3-(4-	MMP2, MDM2, MAPK3, TP53, EGFR	Pathways in cancer, PI3K-Akt signaling pathway, EGFR tyrosine kinase inhibitor resistance	Lung cancer (A549), Normal kidney cells (CV-1)	None	• n-Hexane isolate (InH-2) showed moderate cytotoxic activity against A549 (IC50 31.74 μg/mL) with selectivity over CV-1 cells<br>• Network pharmacology identified MMP-2 as the key protein target in the cancer pathway<br>• Molecular docking revealed dehydroabietic acid binds MMP-2 more stably (–9.5 kcal/mol) than the native ligand	[53]

	Amino-1,2,5-oxadiazol-3-yl)-6-fluoroquinoxalin-2-yl]-1,2,5-oxadiazol-3-amine, Dibenz[b,d]cycloheptanone 1,2,9-trimethoxy					and doxorubicin, suggesting potent inhibition of proliferation, apoptosis induction and anti-migration in lung cancer	
Elaeagnus caudata Schltdl. (Leaves)	Amorphigenin, Pinocembrin, Bergapten, Harman, 4'-Hydroxyflavone	HSP90AA1, CASP3, PIK3CA, MTOR, GRB2	PI3K/AKT/mTOR signaling pathway, RAF/ERK signaling pathway, MAPK signaling pathway, Apoptosis	Human NSCLC cells (A549); normal murine fibroblasts (L929) for safety	None	• B2 fraction of methanolic leaf extract is cytotoxic to A549 (IC_{50} = 26.78 µg/mL) but non-toxic to L929 normal cells<br>• Induces apoptosis via caspase-3/6 activation, DNA damage, and oxidative stress (↑MDA, ↓GSH/GST/SOD)<br>• Amorphigenin identified as top candidate with superior HSP90AA1 binding (−11.11 kcal/mol) vs reference geldanamycin; 100 ns MD confirmed stable complex	[54]
Houttuynia cordata Thunb. (Whole herb)	Quercetin, kaempferol, isoramanone, 2-undecanone, dodecanal (flavonoids and volatile constituents)	MMP9, IL6, PPARG, ICAM1, MMP3	Lipid and atherosclerosis, TNF signaling pathway, IL-17 signaling pathway	None (in silico multi-omics and molecular docking)	None	• Eight key targets (IL6, MMP9, PPARG, ICAM1, SELE, MMP3, MMP1, ALOX5) linked to NSCLC prognosis and immune infiltration in TCGA/GEPIA<br>• Stable MMP9-quercetin docking<br>• First report of anti-NSCLC activity of isoramanone	[55]
Taraxacum officinale F.H.Wigg. (Whole plant)	Quercetin, Apigenin, Kaempferol, Luteolin, Taraxasterol	TP53, CASP3, EGFR, AKT1, ESR1, NQO1	Pathways in cancer, PI3K-Akt signaling pathway, Proteoglycans in cancer, Non-small cell lung cancer	Human lung adenocarcinoma cells (A549); mouse fibroblasts (L929) for selectivity	None	• TCMSP screening identified 58 active compounds and 49 overlapping LC targets; PPI refined to 16 hub nodes<br>• Strongest docking: taraxasterol–AKT1 (−9.7), kaempferol–ESR1 (−9.0 kcal/mol); 100 ns MD confirmed stability<br>• Taraxasterol (25 µmol/L, 48 h) reduced A549 viability to 56.26% with selectivity vs L929 (>80%)<br>• SEM confirmed selective surface	[56]

						disruption in A549 only	
Crinum bulbispermum (Burm.f.) Milne-Redh. & Schweick. (Bulbs); *Pancratium maritimum* L. (Bulbs, Flowers); *Hippeastrum vittatum* (L'Hér.) Herb.; *Centaurea scoparia* Sieber ex Spreng. (Flowering aerial parts)	Crinamine, Ismine, Lycorine, Hemanthidine, Acetyllycoramine	AR, EGFR, ESR1, ERBB2, MAP2K1	Pathways in cancer, Central carbon metabolism in cancer, EGFR tyrosine kinase inhibitor resistance, Endocrine resistance, Non-small cell lung cancer	Human non-small cell lung cancer (H-125), human leukemia (CCRF-CEM)	None	• Phytoconstituents, particularly crinamine, lycorine, and hemanthidine, showed potent cytotoxic activity against NSCLC cells<br>• Network pharmacology and molecular docking revealed a multi-target mechanism for compounds like ismine, acetyllycoramine, and pluviine<br>• The key protein targets identified were Androgen Receptor (AR), Epidermal Growth Factor Receptor (EGFR), and Estrogen-Sensitive Receptor alpha (ESR1)<br>• Ismine showed the most stable binding interactions with AR, EGFR, and ESR1, positioning it as a promising multi-target candidate for NSCLC treatment	[57]
Cinnamomum tamala (Buch.-Ham.) T.Nees & C.H.Eberm. (Bark)	Cinnamaldehyde, δ-Cadinene, γ-Gurjunene, α-Muurolol, Ethyl cinnamate	JUN, TP53, IL6, MAPK3, HIF1A, CASP3	TNF signaling pathway, IL-17 signaling pathway, Apoptosis, Pathways in cancer, MAPK signaling pathway	Human non-small cell lung cancer (A549) cells, Human embryonic kidney (HEK293) cells	None	• Essential oil of C. tamala (CTEO) shows selective cytotoxicity against A549 NSCLC cells while sparing normal cells<br>• Cinnamaldehyde, ethyl cinnamate, and acetophenone are identified as the potential key bioactive compounds<br>• CTEO induces apoptosis through cell cycle arrest, ROS accumulation, mitochondrial depolarization, and activation of caspases<br>• The anti-cancer mechanism is linked to hub genes (JUN, TP53, IL6, etc.) and involves modulation of TNF, IL-17, and MAPK signaling pathways	[58]
Chrysopogon zizanioides (L.) Roberty (Roots, essential oil – CZEO)	Isovalencenol, α-Vetivol, Khusimol, Vetiselinenol, α-Vetivone	AKT1, PIK3R1, STAT3, PIK3CA, MAPK1	PI3K-Akt signaling pathway, Non-small cell lung cancer pathway,	Human lung adenocarcinoma cells (A549); HDFn (normal neonatal	None	• CZEO reduced A549 viability (IC_{50} = 167.82 ± 6.51 μg/mL) with selectivity over normal HDFn cells<br>• Sesquiterpenoid-rich profile (93%) with isovalencenol, α-vetivol and khusimol	[59]

			EGFR tyrosine kinase inhibitor resistance, Prolactin signaling pathway, Endocrine resistance	fibroblasts)		dominating<br>• Network pharmacology identified AKT1 and STAT3 as common hub targets across both cancers<br>• Docking + 200 ns MD + MM/GBSA: rosifoliol and α-vetivone exceeded reference inhibitor binding affinities; α-vetivone preferentially stabilised AKT1, rosifoliol preferred STAT3	
Melaleuca quinquenervia (Cav.) S.T. Blake (Leaves, essential oil – MQLEO)	1,8-Cineole, 1R-α-Pinene, Viridiflorol, (+)-3-Carene, trans-Ocimenol	ESR1, CASP3, PPARG, PTGS2, HSP90AA1	Pathways in cancer, TNF signaling pathway, IL-17 signaling pathway, Estrogen signaling pathway, Chemical carcinogenesis – receptor activation	Human lung adenocarcinoma cells (A549); MCF-7, HepG-2; Vero (normal control)	None	• MQLEO selectively cytotoxic to A549 (IC_{50} = 18.09 μg/mL, SI = 4.30, meeting NCI criteria) and lower toxicity to Vero (CC_{50} = 77.76 μg/mL)<br>• Inhibits migration (62–68% wound closure reduction), induces apoptosis (24.32% vs 0.7% in controls) and G0/G1 cell cycle arrest<br>• Network pharmacology identified ESR1, CASP3, PPARG, PTGS2 as core hub targets<br>• Docking: strongest binding to PTGS2 by fenchol (−6.90 kcal/mol) and trans-verbenol (−6.70 kcal/mol)	[60]

Table S2. Network Pharmacology-Based Studies on Plant-Derived Compounds in Breast Cancer.

**Representative Plant Species/Formula/ Compound / /Plant part**	**Major Compound Classes**	**Top 5 Hub Genes**	**Enriched Pathways**	**In vitro Models**	**In vivo Models**	**Clinical Relevance**	Ref
Piper nigrum L. (Leaves)	Sesamin, Piperettine, Curcumin, Pipernonaline, Lupenone	NF-κB1, TLR-4, STAT-3, HIF-1α, CXCR-4	NF-κB signaling pathway, JAK-STAT signaling pathway, HIF-1 signaling pathway, Toll-	Human breast cancer cells (MDA-MB-231); rat mammary tumor cells (LA7); HEK-293	None	• PNLE (17.06% yield) selectively cytotoxic to MDA-MB-231 (IC_{50} = 42 ± 15 μg/mL at 72 h) without affecting HEK-293 cells<br>• Inhibited cell migration (scratch assay) and colony formation; induced apoptosis confirmed by DAPI-PI, Annexin V-FITC/PI	[99]

			like receptor signaling pathway, Pathways in cancer	(selectivity control); LPS-induced RAW 264.7 (inflammation model)		and AO/EtBr staining, with mitochondrial membrane depolarization (JC-1)<br>• Anti-inflammatory: reduced LPS-induced IL-6 and TNF-α and intracellular ROS in RAW 264.7 cells<br>• Docking: sesamin showed the strongest binding affinities to STAT-3 (−8.8), HIF-1α (−8.2), NF-κB1 (−8.4) and TLR-4 (−6.6 kcal/mol), identifying it as the lead bioactive scaffold	
Punica granatum L. (Seeds); *Beta vulgaris* L. (Root)	Cyanidin 3-glucoside, Betalains, Betaxanthin	TP53, IL6, TNF, AKT1, EGFR	Antifolate resistance, PI3K-Akt signaling pathway	Human triple-negative breast cancer cells (MDA-MB-231)	None	• Anthocyanin/betalain-rich extracts show dose-dependent cytotoxicity against MDA-MB-231 cells<br>• Cyanidin 3-glucoside, betalains and betaxanthin bind strongly to TP53 (−7.1, −7.1, −6.9 kcal/mol), TNF and IL6<br>• Combination with low-dose X-rays (1.35 mGy) enhances radiosensitivity vs. extract alone<br>• 100 μg extract + radiation gave the strongest cytotoxic effect, suggesting use as natural radiosensitizers	[100]
Enhalus acoroides (L.f.) Royle (Whole Plant)	Luteolin, Luteolin-O-sulphate, Thalassiolin C, Myricetin	EGFR, HER2 (ERBB2), HIF-1α, ESR2, AKT1	ErbB signaling pathway, EGFR tyrosine kinase inhibitor resistance, PI3K-Akt signaling pathway, Ras signaling pathway	Human breast cancer cell lines (MCF-7, MDA-MB-231), Normal breast epithelial cell line (MCF-10A)	None	• Ethanol extract of E. acoroides shows potent antioxidant and antiproliferative activity against breast cancer cells.<br>• The anticancer mechanism involves the inhibition of the HER2/EGFR/HIF-1α pathway<br>• Compounds like Luteolin and Thalassiolin C demonstrate high binding affinity to EGFR, HER2, and HIF-1α receptors<br>• The extract downregulates HIF-1α, EGFR, and HER2 expression in MCF-7 cells	[102]
Cissus trifoliata (L.)	Presqualene	CASP3,	Sesquiterpenoid	Hepatocellular	None	• Hexane and chloroform/methanol	[51]

L. (Leaves)	diphosphate, Phytol, Ursolic acid, γ-Linolenic acid, α-Tocopherolquinone	PARP1, BAX, BCL2, STAT3	and triterpenoid biosynthesis, Biosynthesis of unsaturated fatty acids, Steroid biosynthesis	carcinoma (HepG2, Hep3B), cervical (HeLa), prostate (PC3), lung (A549), and breast (MCF7) cancer cell lines; IHH (normal hepatocyte control)		extracts showed significant cytotoxic activity, particularly against hepatocellular carcinoma cell lines (HepG2 and Hep3B)<br>• Metabolomic analysis identified sesquiterpenoid and triterpenoid biosynthesis as the most significant pathway<br>• Network pharmacology revealed that compounds like ursolic acid, phytol, and γ-linolenic acid target key apoptotic genes, including CASP3, BAX, and BCL2	
Astragalus mongholicus Bunge (Root)	Astragalus Polysaccharides (APS)	CCNB1, CDC6, P53, TOP2A, AURKB	p53 signaling pathway, Cell cycle, Neuroactive ligand-receptor interaction, Cytokine-cytokine receptor interaction	Human breast cancer cells (MCF-7, MDA-MB-231)	None	• APS inhibits proliferation, migration, and invasion of breast cancer cells<br>• The anti-cancer mechanism involves the downregulation of CCNB1 and CDC6 and the upregulation of the tumor suppressor P53<br>• The therapeutic effects of APS are linked to the p53 signaling pathway	[63]
Fritillaria cirrhosa D.Don (Bulb)	Peiminine, Imperialine-3-β-glucoside, Imperialine, Peimine, Peonidin	CDK2, AKT1, TNF, SRC, EGFR	PI3K-Akt signaling pathway, Pathways in cancer, Neuroactive ligand-receptor interaction, Metabolic pathways	Human breast cancer cell lines (MCF-7, MDA-MB-231, MDA-MB-468), Mouse mammary carcinoma cell line (4T1)	None	• Ethyl acetate extract showed the highest anticancer activity against various breast cancer cell lines<br>• Peiminine showed the highest binding energy to CDK2, a key regulator of the cell cycle<br>• Imperialine-3-β-glucoside was identified as a major active compound<br>• The anticancer mechanism is suggested to be through the deactivation of Akt1 in the PI3K-Akt signaling pathway	[64]
Polygonatum sibiricum Redouté (Roots)	Sedanolide, Baicalein, 6-Gingerol, Stearidonic acid, α-	ESR1, PPARG, FGF2,	MAPK signaling pathway, PI3K-AKT signaling	Human triple-negative breast cancer cells	None	• First isolation of P. sibiricum-derived exosome-like nanoparticles (PSELNs, ~142 nm) containing 18 proteins and 357	[65]

	Linolenic acid	CAV1, AKT1	pathway, AMPK signaling pathway	(MDA-MB-231)		metabolites<br>• Network pharmacology identified 23 anti-BC hub genes; GEO data confirmed ESR1 upregulation and CAV1/FGF2/PPARG downregulation, all correlated with survival<br>• 100 ns MD confirmed stable binding of sedanolide/6-gingerol–ESR1 and baicalein–PPARG<br>• PSELNs significantly inhibited MDA-MB-231 viability ($p < 0.001$)	
Prunella vulgaris L. (Spica)	Oleanolic acid, Ursolic acid, Betulinic acid, Salviaflaside, Rosmarinic acid	PTGS2, CYP19A1, ESR2, CYP17A1, MAPK3	Ovarian steroidogenesi, Prolactin signaling pathway, TNF signaling pathway, NOD-like receptor signaling pathway	Human breast cancer cell line (MDA-MB-231), Murine macrophage cell line (RAW 264.7)	None	• The anti-breast cancer activity of P. vulgaris is strongest during its early development (heading stage) and decreases as the plant matures<br>• Triterpenoids, specifically oleanolic acid and ursolic acid, are the primary components responsible for the anti-breast cancer effects<br>• The anticancer mechanism is linked to the regulation of the Ovarian steroidogenesis and Prolactin signaling pathways, targeting genes like PTGS2, CYP19A1, and MAPK3	[66]
Actaea vaginata (Maxim.) Franch. (Whole plant)	Beesioside O	STAT3, HIF1A, HSP90AA1, HSP90AB1, PIK3CA	PI3K-Akt signaling pathway, HIF-1 signaling pathway, Pathways in cancer, Chemical carcinogenesis receptor activation	Human breast cancer cells (MCF-7, MDA-MB-231)	None	• Beesioside O (BO) inhibits the proliferation, colony formation, and invasion of breast cancer cells<br>• STAT3 is identified as the pivotal target of BO<br>• BO downregulates STAT3 protein expression, indicating it may act as a STAT3-degrading agent<br>• The anticancer effect is linked to the PI3K-Akt and HIF-1 signaling pathways	[67]

Aralia chinensis L. (Roots, Stems, Leaves – essential oils)	Heneicosane, Docosane, Tetracosane, Octadecane, Pentacosane	AKT1, SRC, EGFR, STAT3, MAPK3	PI3K-Akt signaling pathway, MAPK signaling pathway, EGFR signaling pathway, STAT3 signaling pathway	Human breast cancer cells (MCF-7)	None	• Essential oils from roots, stems, and leaves identified by GC-MS (35, 35, and 24 constituents, respectively); alkanes dominate stems/leaves, while roots contain alkanes, alcohols, and acids<br>• CCK-8 assay confirmed dose-dependent inhibition of MCF-7 proliferation (root 77%, stem 64%, leaf 62%)<br>• Network pharmacology + docking identified AKT1, SRC, EGFR, STAT3, MAPK3 as core targets; common alkane components (octadecane, heneicosane, docosane) showed good binding affinity	[68]
Camellia sinensis (L.) Kuntze (Leaves)	Theacitrin C, Plathymenin, Catechin 7-O-gallate, Epigallocatechin 3,5-di-O-gallate, Caffeine	ESR1, CTNNB1, AR, MDM2, HIF1A	Steroid hormone biosynthesis, Endocrine resistance, Breast cancer pathway, Pathways in cancer, Wnt signaling pathway	Human ER+ breast cancer cells (MCF-7); L929 normal fibroblasts (selectivity)	None	• HRLC-MS/MS identified 11 phytocompounds; 36 mutual targets and 8 hub genes identified<br>• Theacitrin C–CTNNB1 (−6.8 kcal/mol) and Plathymenin–ESR1 (−9.4 kcal/mol) showed strongest binding, 200 ns MD outperformed Tamoxifen<br>• Phytosome formulation (519 nm, $\zeta = -23$ mV) selectively cytotoxic to MCF-7 (IC_{50} = 445.55 μg/mL) with high L929 biocompatibility	[69]
Tinospora cordifolia (Willd.) Miers (Stem) & *Zingiber officinale* Roscoe (Rhizome)	Berberine, Palmitine, Beta-sitosterol, Curcumin	MMP9, ER-α, MMP2, ALOX5	Apoptosis, Cell Cycle Arrest, Inhibition of DNA Synthesis and Migration	Human breast cancer cell line (MCF-7)	None	• Combination treatment is more synergistic and potent than individual plant extracts in inhibiting cancer cell growth<br>• The combinatorial treatment inhibits DNA synthesis, migration, and induces apoptosis in MCF-7 cells<br>• The treatment arrests the cell cycle significantly at the G_0/G_1 phase<br>• The anticancer effect is mediated through significant downregulation of MMP9	[70]

						mRNA and upregulation of its downstream target, ER-α	
Sophora alopecuroides L. (plant part not specified)	Kurarinone, Kaempferide, DOF (2-(3,4-dihydroxyphenyl)-5,7-dihydroxy-6,8-dimethoxychromen-4-one), Sophocarpine, Formononetin	AKT1, TNF, EGFR, CDK2, HSP90AA1	PI3K-AKT, FoxO, MAPK, Ras, Rap1	MCF-7/ADR (drug-resistant), MCF-7 (parental)	None	• SaL-30 reverses multidrug resistance in MCF-7/ADR (IC_{50} = 8.94 ± 0.33 μg/mL); strong synergy with adriamycin (CI = 0.3258); reversal index up to 9.80-fold<br>• Sophocarpine is the most active single alkaloid (IC50 7.92 μM with ADR)<br>• Mechanism: PI3K-AKT pathway inhibition with dose-dependent apoptosis induction (Hoechst-33342, Calcein-AM/PI staining)<br>• SACG comprises 13 compounds (5 flavonoids + 8 quinolizidine alkaloids); docking confirms Kurarinone binding to TNF (−10.3), AKT1 (−10.0), CDK2 (−9.9 kcal/mol)	[71]
Dracaena cochinchinensis (Lour.) S.C.Chen (Resin)	Quercetin, Liquiritigenin, Loureirin A, Resveratrol, Pterostilbene	AKT1, ESR1, EGFR, SRC, CASP3	PI3K-Akt signaling pathway, Pathways in cancer, MAPK signaling pathway, Ras signaling pathway, Estrogen signaling pathway	Human breast cancer cell line (MCF-7)	None	• Sanguis Draconis Flavones (SDF) from Resina Draconis significantly inhibit the proliferation of MCF-7 breast cancer cells<br>• The anticancer effect is mediated by inducing S-phase cell cycle arrest and apoptosis<br>• The primary molecular mechanism involves the regulation of the PI3K/AKT signaling pathway and its key hub targets (e.g., AKT1, ESR1, EGFR)<br>• Flavonoid compounds were identified as the most important active components with good binding activity to the hub targets	[72]
Hylomecon japonica (Thunb.) Prantl (Whole Plant)	6-Methoxydihydrosanguinarine (6-MDS)	PIK3CA, AKT1, MTOR, EGFR,	PI3K/AKT/mTOR signaling pathway, MAPK signaling	Human breast cancer cell line (MCF-7)	None	• 6-MDS, a natural alkaloid, inhibits proliferation and induces both apoptosis and autophagy in MCF-7 breast cancer cells	[73]

		HSP90AA1	pathway, ErbB signaling pathway, Pathways in cancer			• The anticancer mechanism is driven by the accumulation of reactive oxygen species (ROS)<br>• ROS accumulation suppresses the PI3K/AKT/mTOR signaling pathway, leading to cell death<br>• Network pharmacology and molecular docking confirmed the interaction between 6-MDS and the PI3K/AKT/mTOR pathway	
Ajuga decumbens Thunb. (Whole Plant)	8-O-acetylharpagide (prodrug), dihydrogen-harpagenin and a related active metabolite	AKT1, MMP9, STAT3, EGFR, CASP3	PI3K/AKT signaling pathway, AKT/NF-κB/MMP9 signaling axis, HIF-1 signaling pathway, Ras signaling pathway	None	4T1 murine breast cancer model in female Balb-C mice	• 8-O-acetylharpagide is a prodrug that is metabolized into active compounds (notably M5) in vivo<br>• The anticancer effect is mediated by its metabolites, which inhibit breast cancer progression by suppressing the AKT/NF-κB/MMP9 signaling axis<br>• Metabolites M3 and M5 show strong binding affinity to key cancer-related targets, including AKT1 and MMP9	[74]
Selaginella bryopteris (L.) Baker (Whole Plant)	Lanaroflavone, Sequoiaflavone, Amentoflavone, Heveaflavone, Hinokiflavone derivatives	EGFR, TP53, TNF, STAT3, VEGFA	EGFR tyrosine kinase inhibitor resistance, Prolactin signaling pathway, HIF-1 signaling pathway, Rap1 signaling pathway, Pathways in cancer	MCF-7	None	• *S. bryopteris* crude extract inhibits the growth (IC_{50} = 78.34 μg/mL), migration, and invasion of MCF-7 breast cancer cells<br>• Network pharmacology identified EGFR, TP53, TNF, STAT3, and VEGFA as top hub genes involved in the anticancer mechanism<br>• Lanaroflavone and sequoiaflavone were identified as key compounds, showing stable and high-affinity binding to EGFR and CTNNB1, respectively, in molecular docking and dynamics simulations	[75]
Asparagus racemosus Willd. (Aerial Parts)	Beta-sitosterol, Quercetin, Kaempferol, Stigmasterol,	STAT3, EGFR, AKT1, ESR1,	PI3K-Akt, ERBB/ERBB2, EGFR tyrosine kinase inhibitor	MDA-MB-231 (TNBC), HEK-293 (normal control)	None	• Aqueous extract selectively cytotoxic to MDA-MB-231 (IC_{50} = 90.44 μg/mL) vs. HEK-293<br>• Induces G1 phase arrest and early	[76]

	Racemosol	SRC	resistance, estrogen signaling, FoxO			apoptosis (21.70%)<br>• Strongest docking: beta-sitosterol–AKT1 (−10.9) and quercetin–ERBB2 (−9.5 kcal/mol), confirmed by 100 ns MD	
Caesalpinia pulcherrima (L.) Sw. (Whole Plant)	Quercetin, Ellagic acid, Cyanidin, Catechin	ESR1, ESR2, ESRRA, MET, PIK3R1	Estrogen signaling pathway, ErbB signaling pathway, PI3K-Akt signaling pathway, MAPK signaling pathway, Ras signaling pathway	Human breast cancer cell line (MCF-7)	N-Methyl-N-nitrosourea (MNU)-induced mammary carcinoma in female Sprague-Dawley rats	• Four active compounds (quercetin, ellagic acid, cyanidin, catechin) were identified as having therapeutic potential against breast cancer based on ADME criteria<br>• The anticancer mechanism is linked to key targets in the estrogen signaling pathway, such as ESR1, ESR2, and ESRRA<br>• In vitro, the ethyl acetate fraction of the plant (EAFCP) was shown to regulate and decrease the expression of ER and EGFR proteins in MCF-7 cells<br>• In vivo, EAFCP significantly improved the histopathological status of mammary tissue and reduced ER-α expression in MNU-induced tumors in rats	[77]
Rubia tinctorum L. (Roots)	Acacetin, Alizarin, Anthragallol, 2-Hydroxyanthraquinone, Xanthopurpurin	PLCG1, BCL2, CYP1B1, NSD2, ESR2	Metabolic pathways, Pathways in cancer, Apoptosis	Human breast cancer cells (MCF-7)	None	• Network pharmacology identified flavonoids and anthraquinones as the main active compounds targeting key cancer-related genes like PLCG1 and BCL2<br>• The chloroform root extract showed the strongest cytotoxic activity against the MCF-7 breast cancer cell line<br>• Molecular docking revealed that anthragallol and alizarin had the most stable interactions with key protein targets	[78]
Marantodes pumilum (Blume) Kuntze (Herb)	Apigenin, Kaempferol, Gallic acid, Pyrogallol	EGFR, CDK2, ESR1, CCND1, HSPA8	Pathways in cancer, Breast cancer pathway, Estrogen signaling	Human breast cancer cell lines (MCF-7 and MDA-MB-231)	None	• Computational analysis identified that compounds like apigenin, kaempferol, gallic acid, and pyrogallol target key proteins involved in cancer<br>• The primary hub genes identified were	[79]

			pathway			EGFR, CDK2, and ESR1, which are strongly associated with breast cancer pathways<br>• Molecular docking showed that apigenin has the highest binding affinity to the EGFR, CDK2, and ESR1 protein targets<br>• The plant extract demonstrated cytotoxic activity against breast cancer cells, with IC50 values of 88.69 μg/ml on MCF-7 and 66.51 μg/ml on MDA-MB-231 cells	
Coleus amboinicus (Lour.) Spreng. (Leaves)	Hexadecanoic acid, ethyl ester, 1,4-Benzenedicarboxylic acid, bis(2-ethylhexyl) ester, (Z,Z,Z)-9,12,15-Octadecatrienoic acid, ethyl ester, 2,4-Di-tert-butylphenol, 3,7,11,15-Tetramethylhexadec-2-en-1-yl acetate	MMP2, MDM2, STAT3, EGFR, KIT	Pathways in cancer, PI3K-Akt signaling pathway, Apoptosis	Breast cancer (MCF-7), Normal kidney cells (CV-1)	None	• Ethyl acetate extract showed moderate cytotoxic activity against breast cancer cells (MCF-7)<br>• The extract exhibited high selectivity, showing no toxicity to normal cells (CV-1)<br>• Network pharmacology identified Matrix Metalloproteinase-2 (MMP-2) as a key protein target for the extract's bioactive compounds<br>• Molecular docking showed that compounds from the extract have a more stable binding to MMP-2 compared to the cancer drug doxorubicin, suggesting a potent ability to inhibit breast cancer proliferation	[53]
Coleus amboinicus Lour. (Leaves)	Resolvin D1, Gibberellin A24, Hexyl 2-furoate, 13(S)-HOTrE, Arachidonic acid	STAT3, IL6, TNF, ACE, AGTR1	Pathways in cancer, PI3K-Akt signaling pathway, Metabolic pathways	Human breast cancer cells (MCF-7), Normal kidney cells (CV-1)	None	• Ethyl acetate extract demonstrates cytotoxic activity against MCF-7 breast cancer cells with a high selectivity index (4.23), indicating it is safer for normal cells<br>• Network pharmacology and bioinformatics analyses identified Signal Transducer and Activator of Transcription 3 (STAT3) as the main therapeutic protein target<br>• Ten potential bioactive compounds were	[80]

						identified in the extract, with literature suggesting their roles in apoptosis, anti-inflammation, and inhibiting proliferation<br>• The study provides a foundational framework for targeting the STAT3 pathway in breast cancer using compounds from C. amboinicus.	
Basella alba L. (Leaves)	Quercetin, Kaempferol, Beta-carotene, Linoleic acid	AKT1, MMP9, EGFR, CDK6, IGF1R	PI3K-Akt signaling pathway, MAPK signaling pathway, EGFR tyrosine kinase inhibitor resistance pathway, Pathways in cancer	Human breast cancer cell line (MCF-7), Normal breast cell line (MCF-10A)	None	• Basella alba methanolic extract exhibits significant dose-dependent antioxidant, anti-inflammatory (COX2 inhibition), and anticancer activities<br>The extract shows selective cytotoxic effects against the MCF-7 breast cancer cell line (IC_{50} = 26.842 µg/mL)<br>• Network pharmacology identified quercetin and beta-carotene as key therapeutic compounds<br>• Molecular docking and simulation studies confirmed the strong and stable binding of quercetin to MMP9 and beta-carotene to AKT1	[82]
Pisum sativum L. (Peels, Leaves, and Stems)	Methyl cis p-coumarate, trans p-Coumaric acid, Liquiritigenin, Kaempferol-O-neohesperidoside	TP53, AKT1, EGFR, ESR2, AKR1B1	EGFR tyrosine kinase inhibitor resistance, HIF-1 signaling pathway, PI3K-Akt signaling pathway, MAPK signaling pathway, Pathways in cancer	Human breast cancer cell line (MCF-7), Human mammary epithelial cell line (MCF-10a)	None	• The leaves and stems extract (PSLS) showed potent and selective cytotoxic activity against MCF-7 breast cancer cells (IC_{50} = 17.67 µg/mL)<br>• Methyl cis p-coumarate, an isolated compound, was the most potent cytotoxic agent with the highest selectivity (IC_{50} = 1.18 µg/mL, SI = 27.42)<br>• Network pharmacology revealed that flavonoids and phenolic acids from the extracts interact with key breast cancer targets, including carbonic anhydrases, AKR1B1, ESR2, and EGFR<br>• PPI analysis identified TP53, AKT1, and	[83]

						EGFR as the main hub protein targets	
Inula aschersoniana Janka (Aerial parts)	Naringin, Rutin, Vanillic acid, Neohesperidin, o-Coumaric acid	AKT1, PPARG, EGFR, MMP9, SERPINE 1	Pathways in cancer, Estrogen signaling pathway, Endocrine resistance, AGE-RAGE signaling pathway, Relaxin signaling pathway	Human breast cancer cells (MCF-7); HUVEC normal cells (selectivity control)	None	• Methanolic extract reduced MCF-7 viability dose-dependently (~77% at 5000 μg/mL, 72 h) with no HUVEC cytotoxicity • Network pharmacology identified 45 overlapping targets between major phytochemicals and breast/colon cancer • Naringin showed strong PPARG binding (−9.4 kcal/mol), exceeding reference rosiglitazone (−8.2 kcal/mol) • Significant α-glucosidase (IC_{50} = 3.74 μg/mL) and GST (IC_{50} = 1.67 μg/mL) inhibition; estrogen signaling and endocrine resistance highlighted as relevant pathways for hormone-dependent breast cancer	[84]
Gladiolus italicus Mill. (Aerial parts)	Hyperoside, Isoquercitrin, Delphinidin-3,5-diglucoside, Myricetin, Kaempferol-3-glucoside	AKT1, TP53, STAT3, TNF, EGFR	PI3K-Akt signaling pathway, VEGF signaling pathway, ErbB signaling pathway, EGFR tyrosine kinase inhibitor resistance, PD-L1/PD-1 checkpoint pathway in cancer	Human breast adenocarcinoma cells (MDA-MB-231)	None	• Ethyl acetate extract showed strongest cytotoxicity against MDA-MB-231 (IC_{50} = 19.85 μg/mL) • Network pharmacology identified 657 unique targets across PD-1 (52), VEGFR2 (85) and FGFR2 (130); hub genes included AKT1, TP53, STAT3, EGFR and TNF • Molecular docking and 100 ns MD validated stable interactions of kaempferol with VEGFR2 and isoquercitrin with FGFR2	[85]
Kalanchoe laciniata (L.) DC. (Whole plant)	Quercetin 3-(2-glucosylrhamnoside), Kaempferol-3-O-glucoside-3-rhamnoside, Rutin, Quercetin 3-O-β-D-	AKT1, TP53, TNF, ALB, EGFR	PI3K-AKT signaling pathway, Estrogen signaling pathway, PD-1	Human triple-negative breast cancer cells (MDA-MB-231); RIN-5F normal pancreatic β-cells	None	• Soxhlet methanolic extract selectively cytotoxic to MDA-MB-231 (IC_{50} = 60.55 μg/mL) vs RIN-5F (IC_{50} = 242.17 μg/mL) • Strongest docking: quercetin 3-(2-glucosylrhamnoside)–BCRP (−16.23) and AKT1 (−13.11 kcal/mol); 100 ns MD	[86]

	xylopyranosyl-(1→2)-β-D-galactopyranoside, Kalanchoside C		checkpoint pathway in cancer, IL-17 signaling pathway, Pathways in cancer	(selectivity)		confirmed stable BCRP-rutin, MRP1- and AKT1-flavonoid complexes • Synergy with doxorubicin at IC30–IC50 (CI < 1) shifting to antagonism at IC70 • qPCR confirmed significant downregulation of AKT1, BCRP, MRP1; P-gp unchanged	
Eriobotrya japonica (Thunb.) Lindl. (Fruit)	Meglutol, 5-Hydroxymethyl-2-furaldehyde, Hyperoside, Afzelin, 2-Methoxy-phloroglucinol	AKT1, PIK3CA, GSK3B, SRC, BCL2	Regulation of cell population proliferation, Apoptosis, Protein phosphorylation, Regulation of intracellular signal transduction	MCF-7 (human breast cancer cells), MCF-10A (normal breast epithelial cells)	None	• Ethanolic extract of E. japonica fruit (EEEJ) demonstrates antioxidant and anti-proliferative properties<br>• Hyperoside and Afzelin are key bioactive compounds with strong binding affinity to cancer-related protein targets and high antioxidant activity<br>• EEEJ and its key compounds (Hyperoside, Afzelin) show selective cytotoxicity against MCF-7 cancer cells with significantly lower toxicity to normal MCF-10A cells<br>• The anticancer mechanism is associated with the modulation of key cancer signaling pathways, with AKT1 identified as a central hub protein	[87]
Arcangelisia flava (L.) Merr. (Root)	Oxo berberine, Dihydroberberine, Mangostanin, a-Mangostin, g-mangostin	PI3KCA, TP53, BCL2, CDK1, EGFR	PI3K-Akt signaling pathway, p53 signaling pathway, Apoptosis, Cell cycle	Human breast cancer cells (T47D)	None	• Oxo berberine is the most abundant compound in the root extract, targeting 84 genes related to breast cancer<br>• In silico analysis shows oxo berberine has a strong binding affinity to key cancer-related receptors like PI3KCA, TP53, BCL2, CDK1, and EGFR<br>• The root extract induces a higher rate of cell death (apoptosis and necrosis) in T47D cells compared to doxorubicin<br>• The extract causes an accumulation of cancer cells in the G1 phase, inhibiting cell	[88]

						cycle progression	
Gymnostachyum febrifugum Benth. (Roots)	Lupeol, Stigmasterol, γ-Sitosterol, Campesterol, Diethyl phthalate	MAPK1, AKT1, TP53, BCL2, EGFR	MAPK signaling pathway, PI3K-AKT signaling pathway, Apoptosis pathway, p53 signaling pathway, Pathways in cancer	Human breast cancer cells (MCF-7); preliminary screening on A549 (lung) and HCT-116 (colon)	None	• GC-MS/MS identified 18 compounds with lupeol dominant (77.69%) • Ethyl acetate extract cytotoxic to MCF-7 (IC_{50} = 68.14 μg/mL, 72 h) with anti-migratory and anti-clonogenic effects • Flow cytometry confirmed 45.30% apoptotic cells • Lupeol showed strongest docking to MAPK1 (−9.7) and AKT1 (−9.2 kcal/mol); 100 ns MD confirmed stable complexes	[89]
Loranthus micranthus Linn. (Leaves and Stems)	Epicatechin, Rutin, Quercetin	IDO1, IDO2, TDO2, CTLA-4	Tryptophan metabolism, nitrogen metabolism, immune checkpoint regulation	Human breast cancer cells (MDA-MB-231, MCF-7); IDO1-transfected HEK293A; co-culture with mouse T cells	DMBA-induced breast cancer in female rats	• LML/LMS extracts dose-dependently reduced IFN-γ-induced kynurenine production without cytotoxicity • Downregulated IDO1/IDO2/TDO2 expression and directly inhibited IDO1 catalytic activity • Restored T-cell viability in co-culture; in vivo decreased CTLA-4 and increased CD4+ T cells, delaying tumor growth	[90]
Clerodendrum infortunatum L. (Leaves)	Clerodin	TP53, CDK2, HSP90AA1, AR, PIK3CG	PI3K-Akt signaling pathway, Pathways in cancer, p53 signaling pathway, Prostate cancer	Human breast cancer cell line (MCF-7), Normal human lymphocyte cells (HLCs)	None	• Clerodin shows selective cytotoxicity against MCF-7 breast cancer cells (IC_{50} = 30.88 μg/mL) while being non-toxic to normal lymphocytes<br>• The anticancer mechanism is mediated by an increase in intracellular reactive oxygen species (ROS) and a decrease in reduced glutathione (GSH)<br>• In silico docking showed high binding affinity of clerodin to farnesyltransferase and phosphoinositide 3-kinase	[91]
Delphinium roylei Munz (Root)	8-hydroxycoumarin, Piperine, Aloesin, Delsemine B, Narwedine	Akt1, SRC, EGFR, IL-6,	PI3K-Akt signaling pathway, Pathways in	Human breast cancer cell lines (MDA-MB-231, MCF-7, MDA-	None	• Ethyl acetate extract of D. roylei showed the highest anticancer activity<br>• 8-hydroxycoumarin was identified as the key bioactive compound with the highest	[92]

		HSP90AA1	cancer, MAPK signaling pathway, MicroRNAs in cancer	MB-468), Mouse mammary carcinoma cell line (4T1)		binding affinity to the hub target protein, Akt1<br>• The anticancer mechanism is primarily associated with the PI3K-Akt signaling pathway<br>• The extract induces apoptosis in breast cancer cells.	
Rauvolfia tetraphylla L. (Leaves)	Ajmaline, Reserpine, Serpentine	YAP1, ESR1, MTOR, EGFR, PIK3CA	Hippo signaling pathway, PI3K/Akt signaling pathway, MAPK signaling pathway	Human breast cancer cell line (MCF-7), Vero cells (normal cell control)	DMBA-induced breast cancer in female Swiss albino mice	• R. tetraphylla extract selectively inhibits proliferation and migration of MCF-7 breast cancer cells<br>• The anticancer mechanism is mediated through the inhibition of the Yes-Associated Protein (YAP), a key regulator of the Hippo pathway<br>• The extract induces apoptosis by upregulating pro-apoptotic BAX and downregulating anti-apoptotic Bcl-2<br>• In vivo, the extract significantly inhibits tumor growth in a DMBA-induced mouse model	[93]
Taraxacum officinale F.H.Wigg. (Extract)	Taraxerol (TRX)	MAPK3 (ERK1), Slug	Adherens junction, ERK/Slug axis	Human triple-negative breast cancer cells (MDA-MB-231)	None	• Taraxerol inhibits migration and invasion of triple-negative breast cancer cells<br>• MAPK3 (ERK1) is the critical target of Taraxerol<br>• The anticancer mechanism is mediated through the ERK/Slug axis	[94]
Opuntia ficus-indica (L.) Mill. (Flowers)	1,2,3-tris-(trimethylsilanyloxy)-propane, D-Fructose, 1,3,4,5,6-pentakis-O-(trimethylsilyl)-, Hexadecanoic acid methyl ester	SRC, PIK3CA, PIK3CB, PIK3CD, JAK2	PI3K-Akt signaling pathway, Pathways in cancer, Coronavirus disease-COVID-19	Human breast cancer cells (MCF7, MDA-MB-231), Human liver cancer cells (HepG2)	None	• Flower extract shows proapoptotic activities, inducing early apoptosis and cell cycle arrest at the sub-G1 phase<br>• The extract increases the expression of p53, cyclin D1, and caspase 3<br>• Network analysis revealed that the anticancer components target the PI3K-Akt pathway	[95]

Nigella sativa L. (Seeds)	Betulinic acid, Stigmasterol, Folic acid	EGFR, MAPK1, MAPK3, ESR1, PTGS2	Estrogen signaling pathway, Endocrine resistance, EGFR tyrosine kinase inhibitor resistance, Pathways in cancer, TNF signaling pathway	Human breast cancer cell line (MDA-MB-231)	DMBA-induced breast cancer in female albino rats	• Betulinic acid and stigmasterol show strong binding affinities to key breast cancer protein targets (EGFR, MAPK1, MAPK3, etc.)<br>• In vitro, betulinic acid and stigmasterol significantly reduce the viability of MDA-MB-231 cells<br>• In vivo, both compounds contribute to the recovery of serum cancer markers (AFP, CA125) and improve breast tissue histology in a DMBA-induced rat model	[96]
Zingiber officinale Roscoe (Rhizome) and *Allium sativum* L. (Bulb)	Not specified	PTEN, CTNNB1, AKT1, TP53, MYC	PTEN signaling, Wnt signaling pathway, MAPK signaling pathway	None	Murine breast cancer model (4T1 cells in BALB/c mice)	• A combined aqueous extract of garlic and ginger significantly reduces tumor size<br>• The combined treatment uniquely increases the expression of the PTEN tumor suppressor gene<br>• The extracts decrease the expression of the oncogene HER2 and exhibit antioxidant effects by increasing glutathione reductase and peroxidase levels<br>• The anti-cancer mechanism is linked to antioxidant activity and the regulation of key cancer-related genes, particularly PTEN	[97]
Artemisia annua L. (Aerial parts)	3-(2-methylpropanoyl)-4-cadinene-3,11-diol, Artemisinin G, O-(2-propenal) coniferaldehyde, (2-glyceryl)-O-coniferaldehyde, Arteamisinin III	NFKB1, MAP2K1, AR, MDM2, PTGS2	Human breast cancer cell line (MDA-MB-231), Prostate (PC-3), Pancreatic (PANC-1), Melanoma (A375) cancer cell lines; Healthy kidney	PC-3, MDA-MB-231, PANC-1, A375	None	• *A. annua* extract demonstrates significant cytotoxic activity against several cancer cell lines, including the breast cancer line MDA-MB-231 (IC_{50} = 86.21 μg/ml)<br>• Key compounds like 3-(2-methylpropanoyl)-4-cadinene-3,11-diol and O-(2-propenal) coniferaldehyde show strong, stable interactions with major cancer-related protein targets<br>• The primary anticancer mechanism	[98]

			cells (Vero E6)			involves the regulation of key targets such as NFKB1 and MAP2K1, and enrichment in pathways critical to cancer, including the breast cancer pathway	

Table S3. Network Pharmacology-Based Studies on Plant-Derived Compounds in Colorectal Cancer.

Plant Species/Plant part	Top 5 Major Compounds	Top 5 Hub Genes	Enriched Pathways	In vitro Models	In vivo Models	Key Findings	Ref.
Beta vulgaris L. (Root)	Quercetin, Galangin, Hesperidin, Farrerol, Betanin	CDK4, TP53, CCNE1, MCM7, RBL1	Cell cycle, p53 signaling pathway, PI3K-Akt signaling pathway, MAPK signaling pathway, Pathways in cancer	Human colorectal cancer cell line (HT-29)	None	• Ethanolic beetroot extract inhibits HT-29 proliferation with IC50 = 39.03 ± 1.4 μg/mL<br>• Strongest docking: Betanin–CDK4 (−8.11 kcal/mol), Hesperidin–CDK4 (−7.96), Galangin–CDK4 (−7.13), Quercetin–CDK4 (−7.04)<br>• Mechanism: G1/S cell-cycle arrest via CDK4 inhibition; quercetin downregulates 9/11 cell-cycle genes<br>• Suggests beetroot as natural chemopreventive agent in UC-related CRC	[101]
Pueraria montana var. lobata (Willd.) Maesen & S.M.Almeida ex Sanjappa & Predeep (Dried root)	Sandwicensin, Genistein, Medicarpin, Haginin A, Isopterofuran	MAPK3, MTOR, HSP90AA1, CCND1, MAPK1	Pathways in cancer, Cell cycle, Neuroactive ligand–receptor interaction	Human colorectal cancer cell lines (SW480, SW620)	None	• Fourteen compounds were isolated from the dichloromethane extract of the root, seven for the first time from this plant<br>• Sandwicensin exhibited the most potent inhibitory activity against colon cancer cells<br>•Network pharmacology suggests Sandwicensin exerts anti-tumor effects by regulating cell cycle-related (CCND1, CDK4) and proliferation-related (MAPK3, MTOR) proteins	[106]
Scutellaria barbata D. Don (Aerial parts)	Barbatin A (BZL-5), Scutebata O, Scutebata G,	STAT3, HSP90AA1,	Pathways in cancer, PI3K-Akt signaling	Human colorectal cancer cells (HCT-116); FHC normal	None	• Seven neo-clerodane diterpenes isolated and structurally elucidated by NMR/CD; BZL-5 (barbatin A) emerged as most active	[107]

	Scutebata D, Scutebata F	HIF1A, NFKB1, TLR4	pathway, HIF-1 signaling pathway, Cellular senescence, Ras signaling pathway	colon cells (selectivity)		• Strongest docking: BZL-5–HIF1A (−9.989 kcal/mol); 250 ns MD confirmed BZL-5/HIF1A as most stable complex<br>• BZL-5 selectively inhibited HCT-116 (IC_{50} = 4.35 μM) without FHC cytotoxicity, suppressed migration and induced apoptosis dose-dependently<br>• qRT-PCR confirmed downregulation of STAT3, HSP90AA1, HIF1A, NFKB1, TLR4, PIK3CA mRNA	
Patrinia heterophylla Bunge (Whole aerial part)	Asperglaucide, Orotinin, Bolusanthol B, Morusin, Sitosterol	EGFR, PIK3CA, AKT1, GSK3B, PTGS2	ErbB signaling pathway, FoxO signaling pathway	Human colon cancer cells (HCT116, HT29, LoVo)	None	• Water extract of P. heterophylla (PHW) exhibits selective cytotoxic activity against colon cancer cells<br>• The anticancer mechanism is mediated through the inhibition of the ErbB signaling pathway<br>PHW downregulates the mRNA and protein expression of key genes in the pathway, including EGFR, PI3K, and AKT<br>• Asperglaucide was identified as a key active component of PHW<br>Asperglaucide inhibits colon cancer cell migration and suppresses the EGFR/PI3K/AKT signaling pathway	[108]
Paeonia lactiflora Pall. (Root)	Paeoniflorin, Kaempferol, β-Sitosterol, (+)-Catechin	IL-6, JUN, AKT1, MAPK8, TNF, STAT3	IL-6/STAT3 signaling pathway, IL-17 signaling pathway, TNF signaling pathway	None	AOM/DSS-induced colitis-associated colorectal cancer (CAC) in Kunming mice	• Network pharmacology and molecular docking identified Interleukin-6 (IL-6) as a key target of paeoniflorin (PF) in CAC<br>• In the mouse model, PF increased survival rates, and decreased the number and size of colon tumors<br>• PF treatment reduced the histological score of colitis and suppressed the expression of proliferation markers Ki-67 and PCNA<br>• The chemopreventive mechanism of PF	[109]

						is associated with the suppression of the IL-6/STAT3 signaling pathway and a reduction in IL-17A levels in the colon	
Sanguisorba officinalis L. (Root)	2α,3α-dihydroxyurs-12,19(29)-dien-28-oic acid β-D-glucopyranosyl ester, 1β-hydroxyrosic acid, 3β,19α-dihydroxyurs-12-en-28-oic acid, Pomeranic acid, Ziyuglycoside II	TNF, TP53, EGFR, CASP3, IL-6, STAT3	TNF-α/NF-κB signaling pathway, PI3K-AKT signaling pathway	None	AOM/DSS-induced colon cancer in C57BL/6 mice	• Processed S. officinalis triterpenoids (TP) have a more potent preventive effect on colon cancer than raw triterpenoids (TR)<br>• The mechanism involves inhibiting the TNF-α/NF-κB signaling pathway, which reduces inflammatory cytokines (p65, COX-2, iNOS)<br>• Triterpenoids decrease cancer cell proliferation and promote apoptosis.<br>• Compound 27 was identified as a key active molecule that directly binds to and forms a stable complex with TNF-α<br>• The treatment leads to hypermethylation of TNF-α and p65 genes, suggesting epigenetic regulation contributes to the anti-tumor effect	[110]
Berberis fortunei Lindl. (Roots, Stems, Leaves)	Berberine, Palmatine, Jatrorrhizine, Tetrandrine, Berbamine	CCND1, HSP90AA1, AKT1, CDC42	Pathways in cancer, Proteoglycans in cancer, Colorectal cancer pathway	Human colorectal cancer cells (HT-29)	None	• Comprehensive profiling: 77 volatile (GC-IMS) and 116 non-volatile (LC-QTOF-MS) compounds across roots, stems, leaves<br>• Anti-HT29 activity ranked roots > stems > leaves<br>• Protoberberine-type alkaloids (berberine, palmatine, jatrorrhizine) more potent at 24 h, while bisbenzylisoquinoline-type alkaloids (berbamine, fangchinoline, tetrandrine) more potent at 48 h<br>• Docking-based network analysis revealed distinct targets: protoberberines bind CCND1 and HSP90AA1, whereas bisbenzylisoquinolines bind AKT1 and CDC42	[111]
Lycium barbarum	*Lycium barbarum*	PMI,	PI3K/AKT	Human colon	BALB/c	• LBP, in combination with oxaliplatin,	[112]

L. (Fruit)	polysaccharide (LBP), Quercetin, Glycitein, Carotenoids, Phenylpropanoids	ABCG2, PI3K, AKT1, SRC, EGFR	signaling pathway, Cancer-related signaling pathways, IL-17 signaling pathway	cancer cells (HCT116), Oxaliplatin-resistant human colon cancer cells (HCT116-OXR)	nude mice with HCT116 and HCT116-OXR cell xenografts	reverses drug resistance in colon cancer cells<br>• The mechanism involves the downregulation of phosphomannose isomerase (PMI) and the drug transporter ABCG2<br>• Inhibition of PMI leads to the suppression of the PI3K/AKT survival pathway, which in turn decreases Bcl-2 and increases Bax expression, promoting apoptosis<br>• LBP combined with oxaliplatin inhibits cancer cell invasion and migration, and induces G1 cell cycle arrest<br>• The combination therapy effectively reduces tumor growth in vivo without causing significant organ toxicity	
Tetradium ruticarpum (A.Juss.) T.G.Hartley (Fruit)	Rutaecarpine, Isorhamnetin, Evodiamine, Quercetin	TNF, MAPK1, TP53, AKT1, RELA	TNF signaling pathway, p53 signaling pathway, Pathways in cancer	Human colorectal cancer cells (HT29, LS180)	None	• Network pharmacology identified rutaecarpine as a key bioactive ingredient against CRC<br>• Rutaecarpine exhibited the strongest inhibitory effect on the proliferation of CRC cells compared to other tested compounds (isorhamnetin, evodiamine, quercetin)<br>• The primary anticancer mechanism involves the suppression of Tumor Necrosis Factor-alpha (TNF-α)<br>• Molecular docking showed a strong binding affinity between rutaecarpine and TNF-α protein<br>• In vitro experiments confirmed that rutaecarpine reduces the release of TNF-α from CRC cells	[113]
Glycyrrhiza	Liquiritin,	TP53,	Pathways in	Human colorectal	SW480	• HPLC identified 7 active compounds in	[114]

uralensis Fisch. (Root)	Glycyrrhizic acid, Isoliquiritoside, Isoliquiritin apioside, Rutin	SRC, STAT3, PIK3CA	cancer, MAPK signaling pathway, PI3K-Akt signaling pathway, microRNAs in cancer, Apoptosis	cancer cells (SW480)	cell xenograft model in BALB/c nude mice	*G. uralensis* extract; network pharmacology indicated TP53, SRC, STAT3, and PIK3CA as core anti-CRC targets<br>• Liquiritin showed dose-dependent inhibition of SW480 proliferation, colony formation, migration, invasion, and induction of apoptosis (superior to glycyrrhizic acid, which lacked dose-dependence)<br>• MD simulation confirmed stable liquiritin–TP53 binding<br>• In vivo, liquiritin dose-dependently reduced xenograft tumor weight without affecting body weight<br>• Mechanism involves activation of p53 (↑ p-p53/p53) and inhibition of p38 MAPK (↓ p-p38/p38)	
Bellardia trixago (L.) All. (Aerial parts)	Rutin, 4-Hydroxybenzoic acid, Ferulic acid, Hyperoside, Delphinidin 3,5-diglucoside	TP53, GSK3B, TNF, ESR1, IL1B	Colorectal cancer pathway, PI3K-Akt signaling pathway, TNF signaling pathway, EGFR tyrosine kinase inhibitor resistance	Human colorectal cancer cells (HT-29, HCT-116); HEK-293 normal cells	None	• First report on phenolic profile and bioactivity of B. trixago; 70% EtOH extract most cytotoxic against HT-29 (IC_{50} = 38.42 μg/mL) and HCT-116 (IC_{50} = 45.65 μg/mL), with selectivity vs HEK-293<br>• Rutin dominated EtOH/70% EtOH extracts; 4-hydroxybenzoic acid major in aqueous/EtOAc<br>• Network pharmacology yielded 58 overlapping CRC targets and identified TP53, GSK3B, TNF, ESR1, IL1B as top hub genes<br>• 100 ns MD confirmed delphinidin 3,5-diglucoside forms most stable complex with PTGS2, with moderate stability on KRas and higher flexibility on EGFR	[116]
Ceratonia siliqua L. (Seeds)	Quercitrin (quercetin-3-O-rhamnoside), 4'-	EGFR, SRC,	VEGF signaling pathway, EGFR	Human colorectal carcinoma cells	None	• Two flavonoids isolated from carob seed methanolic extract; 4'-p-	[117]

	p-Hydroxybenzoylisorhamnetin-3,7-di-O-rhamnoside (2 compounds isolated and identified by $^{1}H/^{13}C$ NMR)	HSP90AA1, VEGFA, PRKCA	tyrosine kinase inhibitor resistance, peptidyl serine phosphorylation, protein/serine/tyrosine kinase activity	(Caco-2); WI-38 normal human fetal fibroblasts (selectivity control)		hydroxybenzoylisorhamnetin-3,7-di-O-rhamnoside shows potent Caco-2 cytotoxicity (IC50 = 9.94 μg/mL, SI = 3.15)<br>• Network pharmacology identified EGFR, SRC, and HSP90AA1 as most interconnected hub targets via PPI analysis<br>• HPLC standardization revealed 1.22 μg% of the active compound in the EtOAc fraction (vs. 0.72 μg% in crude methanolic extract)	
Paracaryum hedgei Aytaç & R.R. Mill (Roots)	Rosmarinic acid, Salvianolic acid B, Salvianolic acid H/I, Sagerinic acid, Caffeic acid	SRC, STAT3, CYP1A1	Retinol metabolism, Steroid hormone biosynthesis, TNF signaling pathway, IL-17 signaling pathway, Pathways in cancer	Human colorectal adenocarcinoma cells (HCT-116; IC_{50} = 96.82 μg/mL, root EtOAc); HEK-293 normal cells	None	• First phytochemical/bioactivity report on P. hedgei; HPLC-ESI-Q-TOF-MS identified 33 compounds, roots dominated by salvianolic acids and rosmarinic acid<br>• Root EtOAc extract induced apoptosis/necrosis in HCT-116 (AO/EB + Annexin V/PI)<br>• Network pharmacology predicted SRC/STAT3/CYP1A1 axis and retinoid/steroid metabolism as anti-cancer mechanism<br>• Docking + 300 ns MD: salvianolic acid B showed strongest multi-target binding	[118]
Rubus ulmifolius Schott (Leaves)	Catechin, Caffeic acid, Gallic acid, D-(-)-Fructofuranose	EGFR, ESR1, PTGS2, STAT3, MMP9	Pathways in cancer, Proteoglycans in cancer, Estrogen signaling pathway, PD-L1/PD-1 checkpoint pathway, HIF-1 signaling pathway	Human colorectal adenocarcinoma cells (HT-29)	None	• EtOAc leaf extract potently cytotoxic to HT-29 (IC_{50} = 2.41 μg/mL), classified as significant per NCI guidelines<br>• Network pharmacology identified 52 overlapping CRC targets shared by ≥2 compounds; PPI ranked EGFR and ESR1 as top hubs (degree = 20)<br>• Catechin showed strongest docking across all four hub targets, exceeding reference inhibitor Stattic for STAT3 (−8.12 vs −7.41 kcal/mol)	[119]
Gymnanthemum	Betaine, Rhamnetin,	COX-2	PI3K-Akt	None	Azoxyme	• The extract (VAEE) mitigates key	[120]

amygdalinum (Delile) Sch.Bip. (Leaves)	Echinenone, Luteolin, Latifoline	(PTGS2, NF-kB (NFKB1), AKT1, EGFR, TNF	signaling pathway, Pathways in cancer, HIF-1 signaling pathway		thane/dextran sulfate sodium (AOM/DSS)-induced colorectal cancer model in mice	markers of colorectal cancer, such as colon shortening and weight loss<br>• It demonstrates potent anti-inflammatory effects by decreasing pro-inflammatory cytokines (IL-6, IL-1β, TNF-α) and increasing the anti-inflammatory cytokine IL-10<br>• VAEE has strong antioxidant properties<br>• It showed a favorable safety profile with no signs of acute toxicity<br>• Molecular docking supports its role as a potential inhibitor of inflammatory targets like COX-2 and NF-kB	
Helicteres isora L. (Fruit)	Rosmarinic acid, Isorhamnetin, Isoscutellarein, 5,8-Dihydroxy-7,4'-dimethoxy flavone, Kaempferol 3-p-coumarate	MAPK1, ERBB2, EGFR, MET, KRAS	PI3K-Akt signaling pathway, MAPK signaling pathway, p53 signaling pathway, EGFR tyrosine kinase inhibitor resistance, FOXO signaling pathway	Human colorectal cancer cells (HCT-116)	None	• Methanolic fruit extract shows strong DPPH antioxidant activity (77.73% RSA at 500 μg/mL) and moderate cytotoxicity against HCT-116 cells (IC_{50} = 13.58 ± 1.12 μM)<br>• Network pharmacology + PPI identified CTNNB1 as central hub; MCODE clustering revealed 5 functional modules linked to cell cycle, apoptosis, inflammation, and ECM remodeling<br>• Rosmarinic acid showed strongest MAPK1 binding (−8.754 kcal/mol), outperforming reference drug Encorafenib (−6.655 kcal/mol); 100 ns MD confirmed complex stability<br>• Survival analysis: high ERBB2 (HR=0.58, p=0.03) and MAPK1 (HR=0.56, p=0.022) expression correlated with improved CRC outcomes	[121]
Biancaea sappan (L.) Tod. (Heartwood)	Brazilin, Brazilein, Sappanchalcone, Protosappanin B,	STAT3, AKT1, MAPK1,	Pathways in cancer, PI3K-AKT signaling	Human CRC cells (HCT 116, KM12SM, HT-29,	MC38 colorectal tumor	• UHPLC-QTOF MS/MS identified 18 compounds; network pharmacology revealed 87 overlapping CSE–CRC targets,	[122]

	Sappanone A	EGFR, TP53	pathway, MAPK signaling pathway, VEGF signaling pathway, JAK-STAT signaling pathway	COLO 205)	xenograft in C57BL/6 mice	refined to 33 core hubs<br>• CSE inhibited proliferation in 4 CRC lines and induced caspase-3/7-dependent apoptosis with G1/S arrest (↑p21, ↓cyclin D1, CDK2)<br>• Mechanism: suppression of STAT3 (Tyr/Ser) and AKT phosphorylation, ↑HO-1 stress response; MAPK pathway unaffected<br>• In vivo: 100 mg/kg oral CSE significantly reduced MC38 tumor weight without body weight loss or liver/kidney toxicity	
Argemone mexicana L. (Leaves)	Berberine, Allocryptopine, Protomexicine, Chelerythrine, Oleandrigenin-β-D-digi (neritaloside)	MAPK1, CDK4, MDM2, PDGFR, PIK3CA	PI3K-Akt signaling pathway, NF-κB signaling pathway	Human skin cancer (A431), Human colon cancer (COLO 320DM)	None	• Ethanolic extract of *A. mexicana* leaves shows the highest dose-dependent cytotoxic activity against skin and colon cancer cell lines<br>• The anticancer effect is mediated through the inhibition of the NF-κB signaling pathway<br>• The extract significantly reduces the expression of the pro-inflammatory cytokine TNF-α in cancer cells<br>• Network pharmacology identified the PI3K-Akt pathway as a primary target, linking the plant's phytochemicals to the observed anti-inflammatory and anticancer activities	[123]
Homalanthus giganteus Zoll. ex Miq. (Leaves)	12-*O*-palmitoyl-7-oxo-5-ene-phorbol-13-acetate, 12-*O*-palmitoyl-phorbol-13-acetate, 7β-hydroxysitosterol, 7α-hydroxysitosterol, β-sitosterol-3-*O*-	STAT3, JUN, PRKCA, GSK3β	Pathways in cancer, Calcium signaling, cAMP signaling, Insulin resistance, Inflammatory mediator	Doxorubicin-sensitive (Colo 205) and doxorubicin-resistant (Colo 320) human colorectal cancer cells	None	• Six compounds isolated for the first time from *H. giganteus*; tigliane diterpene 4 most potent (IC_{50} = 3.58 μM Colo 205, 6.06 μM Colo 320)<br>• DFT confirmed compound 4 chemically more stable than 3 (HOMO-LUMO gap 5.236 vs 4.756 eV)<br>• Compound 4 preferentially binds kinases	[124]

	glucoside		regulation of TRP channels			PRKCA (−5.10) and GSK3β (−4.16 kcal/mol); compound 3 favors transcription factors STAT3/JUN, explaining 3.5-fold activity difference<br>• pkCSM predicted favorable oral bioavailability (74-79%) and acceptable toxicity profile	
Avicennia alba Blume (Leaves)	Avicequinone C, Avicequinone B, Avicenol C, Avicenol B	EGFR, PIK3CA, JAK2, MTOR, JUN	Pathways in cancer, PI3K-Akt signaling pathway, ErbB signaling pathway	Human colorectal adenocarcinoma cells (WiDr)	None	• Network pharmacology identified four key bioactives and 10 hub genes associated with colorectal cancer (CRC)<br>• Avicequinone C showed the highest binding affinity and most stable interactions with the top three hub genes (EGFR, PIK3CA, and JAK2) in molecular docking and dynamics simulations<br>• The ethanolic leaf extract of A. alba demonstrated dose- and time-dependent cytotoxic activity against WiDr colon cancer cells<br>• The IC50 value of the extract was 205.96 μg/mL after 48 hours of treatment<br>• The study validates the integration of computational and in vitro methods to identify the anticancer potential of A. alba	[125]
Saurauia vulcani Korth. (Leaves, fermented into Kombucha)	Beta amyrin, Maslinic acid, Ursolic acid, Beta-sitosterol, Pomolic acid	UBC, CTNNB, AKT1, CASP3, TP53	Pathways in cancer, PI3K-Akt signaling pathway, Wnt signaling pathway, MAPK signaling pathway, Apoptosis	None	Benzo(a) pyrene (B[a]P)-induced colorectal cancer model in male rats.	• Kombucha Pirdot (KP) countered B[a]P-induced damage to colon histoarchitecture<br>• KP treatment decreased the level of the pro-inflammatory cytokine IL-1β<br>• The therapeutic mechanism is linked to hub genes like UBC, CTNNB1, AKT1, TP53, and pathways such as PI3K-Akt and Wnt signaling<br>• In silico analysis suggests potential cross-reactivity between colorectal cancer epitopes and peptides from kombucha	[126]

						microorganisms (*Lactiplantibacillus plantarum* and *Saccharomyces cerevisiae*), indicating an immunomodulatory role.	
Eucommia ulmoides Oliv. (Bark)	Quercetin, Kaempferol, Medioresinol, Luteolin, Catechin	TP53, PPARG, CASP3, BCL2, PTGS1	Cancer-related pathways, Oxidative stress pathways	Murine colon carcinoma cells (CT26), Human liver cancer cells (HepG2)	None	• Steam explosion pretreatment significantly enhances the release of total phenolics, flavonoids, and quercetin from the bark<br>• The steam-exploded extract exhibits superior antioxidant (DPPH radical scavenging, reducing power) and antiproliferative properties compared to the native extract<br>• The extract inhibits the proliferation of CT26 and HepG2 cancer cells in a dose-dependent manner<br>• Network pharmacology analysis identified quercetin as a key compound responsible for the antioxidant and anticancer activities	[127]
Rhus chinensis Mill. (Roots and Stems)	Betulinic acid, Betulonic acid	ASIC2, Calcineurin, NFAT1, MMP-2, MMP-9	ASIC2-mediated calcineurin/NFAT pathway, Glycolysis	Human colorectal cancer cells (SW620, HCT116)	None	• Triterpenoid extract (TER) from Rhus chinensis inhibits proliferation, induces apoptosis, and reduces the invasion of colorectal cancer (CRC) cells<br>• Betulinic acid and betulonic acid were identified as the major active components of the extract<br>• The anticancer mechanism involves the regulation of glycolysis, evidenced by decreased glucose uptake, lactate production, and downregulation of key glycolytic proteins (GLUT1, LDHA, PKM2)<br>• TER targets the acidic tumor microenvironment by downregulating the acid-sensing ion channel 2 (ASIC2) and inhibiting the subsequent	[129]

						calcineurin/NFAT signaling pathway<br>• Inhibition of the ASIC2/calcineurin/NFAT pathway leads to decreased expression of MMP-2 and MMP-9, resulting in reduced cell invasion.	
Rhus chinensis Mill. (Roots and Stems)	Betulinic acid, Betulonic acid, Betulin, Semialactic acid	ENO1, ALDOA, PFKFB3, PKM2, LDHA	Glycolysis, Glutaminolysis, Metabolism of lipids and lipoproteins	Human colorectal cancer cell lines (SW620, HCT116)	SW620 cell xenograft model in BALB/c nude mice	• Triterpenoid extract (TER) inhibits proliferation and induces apoptosis in colorectal cancer cells<br>• The main active compounds are betulinic acid, betulonic acid, and botulin<br>• The anti-cancer effect is mediated by inhibiting key enzymes in glycolysis and glutaminolysis, such as ENO1, ALDOA, and PFKFB3<br>• TER treatment retarded tumor growth in vivo by downregulating these glycolytic enzymes	[128]
Euphorbia dentata Michx. (Whole plant)	Dentatacid A, 9,19-Cyclolanost-25-ene-3β,24R-diol	SRC, ESR1, PIK3CA	PI3K-Akt signaling pathway, SRC signaling pathway	Human colorectal adenocarcinoma cells (HT-29)	None	• Isolated a novel triterpenoid, Dentatacid A, with an unprecedented 2,3-seco-arbor-2,3-dioic skeleton<br>• Dentatacid A demonstrated excellent anti-proliferative activity against the HT-29 colon cancer cell line with an IC50 value of 2.64 μM<br>• Network pharmacology and molecular docking suggest the cytotoxic effect is mediated through the SRC/PI3K/Akt signaling pathway<br>• The study expands the known chemical diversity and potential for resource utilization of this invasive plant species	[130]
Gladiolus italicus Mill. (Corms)	Kaempferol-3-O-glucopyranoside, Quercetin-3-O-glucopyranoside,	PIK3CA, CASP3, EGFR, MMP9,	PI3K-Akt signaling pathway, Endocrine	Human colorectal carcinoma cells (Caco-2)	None	• Ethanolic corm extract and isolated kaempferol-3-O-glucopyranoside inhibit Caco-2 proliferation (IC50 = 7.4 and 6.2 μg/mL, respectively)	[131]

	Betulinic acid, Medicagenic acid, Deoxyerythrolaccin	PTGS2	resistance, Colorectal cancer pathway			• PI3K identified as dominant target via network pharmacology; kaempferol-3-O-glucopyranoside shows strongest docking among 14 dereplicated metabolites<br>• 100 ns MD simulation confirms stable PI3K binding (ΔGbinding = −10.33 kcal/mol)	
Astragalus mongholicus Bunge (Root); *Curcuma sp.* (Rhizome)	Quercetin, Kaempferol, Astragaloside IV, Curcumin, Calycosin	HIF-1α, PFKFB, VE-cadherin, MMPs	HIF-1 signaling pathway, Glycolysis, PI3K-Akt signaling pathway, FoxO signaling pathway	None	Orthotopic colon cancer model (CT26-Lucifer cell line) in BALB/c mice	• The combination (AC) normalizes tumor blood vessels and inhibits tumor metastasis<br>• AC directly targets and binds to HIF-1α, inhibiting its nuclear translocation<br>• This action regulates glycolysis by modulating the key enzyme PFKFB3<br>• It restores vascular integrity by strengthening tumor-associated endothelial cell (TEC) junctions and reducing VE-cadherin internalization	[115]
Glycyrrhiza sp. (Licorice; species not specified) (Root)	Ganodermanotriol, Isolongifolone-9-one, Nordihydroguaiaretic acid, Glabrone, Ammonium glycyrrhizate	ABL1, CTNNB1, FYN, HSP90AA1, EGFR (also HIF-1α)	RAS/RAF/MEK/ERK signaling pathway, HIF-1 signaling pathway, EGFR tyrosine kinase inhibitor regulation, Apoptosis, Pathways in cancer	Human colorectal cancer cells (HCT-116, DLD-1); normal colon cells (NCM460) for safety	AOM/DSS-induced colitis-associated CRC in C57BL/6J mice; HCT-116 xenograft in zebrafish (yolk-sac injection)	• Serum pharmacochemistry (UPLC-Q-Exactive Orbitrap MS) identified 129 compounds in LEs and 36 prototypes/metabolites absorbed into bloodstream<br>• LEs dose-dependently extended survival, reduced tumor number, restored mucosal integrity (goblet cells), induced apoptosis (↑Bax, ↑Caspase-3, ↓Bcl-2), and inhibited Ki67+ proliferation in AOM/DSS mice<br>• In zebrafish xenograft, LEs suppressed both tumor growth and distant micrometastasis<br>• Mechanism: downregulation of EGFR and HIF-1α, suppression of RAS/RAF/MEK/ERK pathway (rescue with RAS activator ML-099 was reversed by LEs-containing serum)	[132]

						• Molecular docking + qPCR validated four key actives binding ABL1, CTNNB1, FYN, HSP90AA1; three of these are co-metabolites, suggesting metabolites mediate direct anti-CRC effects<br>• LEs were non-toxic to normal NCM460 cells, zebrafish embryos and mouse organs	
Nandina domestica Thunb. / (Fruits)	Isoquercitrin, Quercitrin, Berberine, Chlorogenic acid; Caffeic acid	AKT1, CASP3, MAPK1, TP53	Colorectal cancer pathway, Apoptosis, MAPK signaling	Human colorectal carcinoma cell line (HCT-116)	None	• Network pharmacology revealed multi-target synergistic effects<br>• Compounds affect apoptosis and Akt-signaling pathways during early/intermediate adenoma stages (via CASP3, MAPK1); affect p53 and ErbB signaling during late adenoma/carcinoma stages (via AKT1, TP53, MAPK1)<br>• All compounds showed drug-likeness per Lipinski's rule	[50]
Elaeagnus caudata Schltdl. (Leaves)	Amorphigenin, Pinocembrin, Bergapten, Harman, 4'-Hydroxyflavone	HSP90AA1, CASP3, PIK3CA, MTOR, GRB2	PI3K/AKT/mTOR signaling pathway, RAF/ERK signaling pathway, MAPK signaling pathway, Apoptosis	Human CRC cells (HCT116); L929 normal fibroblasts	None	• B2 fraction of methanolic leaf extract is cytotoxic to HCT116 (IC_{50} = 25.70 μg/mL) but non-toxic to L929 normal cells • Induces apoptosis via caspase-3/6 activation, DNA damage, and oxidative stress (↑MDA, ↓GSH/GST/SOD) • Amorphigenin identified as top candidate with superior HSP90AA1 binding (−11.11 kcal/mol) vs reference geldanamycin; UALCAN confirmed HSP90AA1 upregulation in CRC linked to poor prognosis	[54]
Chrysopogon zizanioides (L.) Roberty (Roots, essential oil – CZEO)	Isovalencenol, α-Vetivol, Khusimol, Vetiselinenol, α-Vetivone	STAT3, AKT1, HSP90AA1, TNF, HIF1A	Prolactin signaling pathway, Estrogen signaling pathway,	Human colorectal carcinoma cells (HCT-116); HDFn (normal neonatal fibroblasts)	None	• CZEO (sesquiterpenoid-rich, 93%) selectively reduced HCT-116 viability (IC_{50} = 62.95 ± 2.19 μg/mL) without affecting normal HDFn cells<br>• 40 compounds identified by GC-MS, with isovalencenol, α-vetivol and khusimol	[59]

			Proteoglycans in cancer, IL-17 signaling pathway, PD-L1/PD-1 checkpoint pathway in cancer			as most abundant<br>• Network pharmacology identified STAT3 and AKT1 as central hub targets<br>• Docking + 200 ns MD + MM/GBSA: rosifoliol and α-vetivone outperformed reference inhibitors (MK-2206, Stattic), with α-vetivone preferentially stabilising AKT1 and rosifoliol favouring STAT3	
Inula aschersoniana Janka (Aerial parts)	Naringin, Rutin, Vanillic acid, Neohesperidin, o-Coumaric acid	AKT1, PPARG, EGFR, MMP9, SERPINE 1	Pathways in cancer, Estrogen signaling pathway, Endocrine resistance, AGE-RAGE signaling pathway, Nitrogen metabolism	Human colon adenocarcinoma cells (HT-29); HUVEC normal cells (selectivity control)	None	• Methanolic extract dose- and time-dependently reduced HT-29 viability (~52% at 5000 µg/mL, 72 h) without HUVEC toxicity<br>• HPLC profiling identified 10 phenolics with naringin (14.99 µg/g), rutin (9.6 µg/g) and vanillic acid (5.89 µg/g) as major compounds<br>• Naringin and rutin showed stronger docking to AKT1 (−11.7, −11.5 kcal/mol) and EGFR (−8.9, −9.0 kcal/mol) than reference inhibitors MK-2206 and gefitinib<br>• 50 ns MD and MM/GBSA confirmed AKT1–naringin as the most stable complex (ΔG_bind = −72.51 kcal/mol)	[84]
Gladiolus italicus Mill. (Aerial parts)	Hyperoside, Isoquercitrin, Delphinidin-3,5-diglucoside, Myricetin, Kaempferol-3-glucoside	KT1, TP53, STAT3, TNF, IL6	PI3K-Akt signaling pathway, AGE-RAGE signaling pathway in diabetic complications, Th17 cell differentiation, PD-L1/PD-1 checkpoint pathway in	Human colon adenocarcinoma cells (HT-29)	None	• Ethyl acetate and n-hexane extracts showed comparable cytotoxic effects on HT-29 (IC_{50} = 33.47 and 34.31 µg/mL, respectively)<br>• Network pharmacology with PD-1, VEGFR2 and FGFR2 identified 657 unique targets and core hub genes (AKT1, TP53, STAT3, TNF, IL6)<br>• Molecular docking and 100 ns MD simulations confirmed stable binding of phloretin (PD-1), kaempferol (VEGFR2) and isoquercitrin (FGFR2) to respective	[85]

			cancer, HIF-1 signaling pathway			targets	

Table S4. Network Pharmacology-Based Studies on Plant-Derived Compounds in Prostate Cancer.

**Representative Plant Species/Formula/ Compound / /Plant part**	**Major Compound Classes**	**Top 5 Hub Genes**	**Enriched Pathways**	**In vitro Models**	**In vivo Models**		Ref
Phoebe zhennan S.K.Lee & F.N.Wei (Fruit, essential oil – PZEO)	α-Eudesmol, γ-Eudesmol, Humulene, α-Copaen-11-ol, Elemol	GAPDH, AKT1, ESR1, EGFR, ALB	Pathways in cancer, PI3K/AKT/mTOR signaling pathway, Chemical carcinogenesis-receptor activation, Prolactin signaling pathway, Endocrine resistance	Human prostate cancer cells (LNCaP, PC3); also screened on A2780, A549, HeLa	None	• PZEO (steam-distilled fruit essential oil; sesquiterpenes 78.28%) inhibits LNCaP (IC_{50} = 0.144 μL/mL) and PC3 (IC_{50} = 0.555 μL/mL) cells<br>• Induces apoptosis (32-35% in LNCaP at 0.15-0.2 μL/mL), G2/M arrest in LNCaP, G0/G1 arrest in PC3, and dose/time-dependent migration suppression<br>• Network pharmacology + docking identified humulene, α-copaen-11-ol, and elemol as top active components binding AKT1 (−7.9 kcal/mol)	[135]
*Scleromitrion diffusum (*Willd.) R.J.Wang (Whole plant) & *Scutellaria barbata* D.Don (Whole plant)	Quercetin, Ursolic acid, Apigenin, Baicalein, Scutellarin	AKT1, BCL2, NFKB1, EGFR, ESR1	Apoptosis pathway, Prostate cancer pathway, TNF signaling pathway, PI3K-Akt signaling pathway, NF-κB signaling pathway	Human castration-resistant prostate cancer cells (PC-3)	None	• Network pharmacology identified 108 shared targets with AKT1/BCL2/NFKB axis as core; ursolic acid showed strongest docking to AKT1 (−7.76 kcal/mol), 100 ns MD confirmed AKT1-apigenin as most stable complex<br>• HDH-SBH inhibited PC-3 proliferation (48 h IC_{50} = 1.094 mg/mL), reduced migration by 64.2% at 36 h and induced apoptosis (13.57% vs 4.79%)	[136]

						• Mechanism: downregulation of BCL2 and p-65 protein/mRNA with suppression of AKT1 phosphorylation	
Osmanthus fragrans Lour. (Flower)	Rutin, Verbascoside, Ligustroside, Phillygenin, 4-hydroxyphenyl acetate	PIK3R1, GRB2, PDGFRB, AR	Pathways in cancer, Prostate cancer, EGFR tyrosine kinase inhibitor resistance	Human prostate cancer cells (DU-145), Murine macrophage cells (RAW-264.7)	None	• Ethanolic flower extract shows significant antioxidant, anti-inflammatory, and antiproliferative activity<br>• The extract inhibits the proliferation of DU-145 prostate cancer cells<br>• The anticancer effect is linked to the interaction of its metabolites with key proteins like PIK3R1, GRB2, PDGFRB, and AR<br>• Rutin showed the highest binding affinity to PIK3R1 and AR in molecular docking simulations	[137]
Vitex trifolia L. (Fruit)	Agnuside, 10-O-vanilloylaucubin, Luteolin, Casticin	AKT1, TP53, JUN, EGFR, AR	Pathways in cancer, Prostate cancer, PI3K-Akt signaling, ErbB signaling	Human prostate cancer cells (DU145)	Male Sprague-Dawley rats	• Pharmacokinetic profiles of four absorbed components differ significantly between aqueous and ethanolic extracts<br>• Network pharmacology analysis predicted 15 cancer-related pathways, with prostate cancer being prominent<br>• In vitro experiments showed that the ethanolic extract, casticin, and luteolin significantly inhibit proliferation and induce apoptosis in DU145 cells<br>• The anticancer mechanism is linked to the inhibition of the PI3K-Akt signaling pathway	[138]
Impatiens balsamina L. (Semen)	Glabrone, Solasodine, Aurantiamide, cis-9,10-Epoxystearic acid, Peimine	AR, AKT1, TP53, MAPK1, CCND1	Focal adhesion, Cell cycle, DNA replication, Cellular senescence	Human prostate cancer cells (LNCaP, 22Rv1, PC-3, DU145)	PC-3 xenograft tumor model in male BALB/c nude	• Ethyl acetate extract of Semen Impatientis (EAESI) exerts antitumor effects on prostate cancer (PCa) in vitro and in vivo<br>• EAESI inhibits proliferation and migration, and induces G0/G1-phase arrest and apoptosis in both AR+ and AR- PCa	[139]

					mice	cells • Activating transcription factor 3 (ATF3) is a critical target of EAESI<br>• EAESI downregulates androgen receptor (AR) expression and transcriptional activity via ATF3	
Aspilia pluriseta Schweinf. ex Engl. (Root)	1-Heneicosanol, Lanosterol, Andrographolide, Retinoic acid, 2-pyrrolidinone	MAPK3, MAPK1, IL6, TP53, ESR1	PI3K/Akt signaling pathway, MAPK signaling pathway, p53 signaling pathway	Human prostate cancer cells (DU-145), Non-cancerous kidney epithelial cells (Vero E6)	None	• Root extracts selectively inhibited the proliferation of DU-145 prostate cancer cells with low toxicity to non-cancerous Vero E6 cells<br>The ethyl acetate fraction showed the highest selectivity for prostate cancer cells<br>• Key compounds like lanosterol and retinoic acid showed strong binding affinity to hub proteins such as MAPK1, MAPK3, and ESR1<br>• The anticancer mechanism is linked to the regulation of the PI3K/Akt, MAPK, and p53 signaling pathways<br>• Treatment led to the downregulation of AR, BCL-2, and CDK1 expression and upregulation of p53 and caspase-3, indicating cell cycle inhibition and apoptosis induction	[140]
Tetrapleura tetraptera (Schumach. & Thonn.) Taub. (Fruit, pulp, seed)	Luteolin, Scopoletin	PIK3R1, EGFR, ERBB2, MMP9, AR, AKT1, IGF1R, GSK3B	MAPK, PI3K/AKT, P53	Human prostate cancer cells (PC3, LNCaP), Normal prostatic epithelial cells (PNT2)	None	• Ethyl acetate fruit extract showed the most potent and selective anticancer activity against PC3 and LNCaP prostate cancer cells<br>• Aqueous fruit extract demonstrated significant antioxidant activity<br>• Network pharmacology and molecular docking identified luteolin and scopoletin as key metabolites contributing to the cytotoxic effects<br>• Luteolin showed a strong binding affinity for MMP9 and AR	[141]

						• The anticancer mechanism is suggested to be through the modulation of MAPK, PI3K/AKT, and p53 signaling pathways	
Euphorbia ingens E.Mey. ex Boiss. (Root)	6-pentylidene-4,5-secoandrostane-4,17.beta.-diol, 2-bornanol, 1-octadecene, Andrographolide, Squalene	ESR1, IL6, MMP9, CDK2, MAP2K1	PI3K/Akt signaling, MAPK signaling, p53 signaling	Human prostate cancer cells (DU-145), Non-cancerous kidney epithelial cells (Vero E6)	None	• Ethyl acetate fraction of E. ingens root has selective antiproliferative activity against DU-145 prostate cancer cells<br>• The mechanism is linked to the regulation of PI3K/Akt, MAPK, and p53 signaling pathways<br>• The extract downregulates AR and BCL-2 expression while upregulating p53 and caspase-3<br>• Identified compounds like andrographolide and squalene likely contribute to the anticancer activity	[142]
Salvia rosmarinus Spenn. (Leaf)	Carnosol, Carnosic acid, Rosmarinic acid	EGFR, TP53, ERBB2, IGFBP3, MMP-2	MAPK signaling, PI3K/AKT signaling, Calcium signaling, Central carbon metabolism in cancer	Human prostate cancer cells (DU-145)	None	• Aqueous extract of rosemary leaf showed greater cytotoxicity against DU-145 cells than the ethanolic extract<br>• Carnosol and rosmarinic acid showed strong binding affinities for key androgen-independent targets like EGFR, ERBB2, and TP53<br>• Rosmarinic acid was identified as a promising candidate due to its strong target interaction and low toxicity profile<br>• The study demonstrates the potential of rosemary extracts for treating androgen receptor (AR)-negative prostate cancer	[143]
Perilla frutescens (L.) Britton (Leaf)	Rosmarinic acid, Tuberonic acid, Scutellarein-7-O-glucuronide, 5′-gluco-pyranosyoxyjasmanic acid, n-octanoylsucrose	FYN, PDGFRA, PDGFRB, PIK3R1, ITGB3	Prostate cancer, AMPK signaling, EGFR tyrosine kinase inhibitor resistance, PI3K/Akt signaling	Human prostate cancer cells (DU-145)	None	• Ethanolic extract of P. frutescens leaves was the most cytotoxic against DU-145 prostate cancer cells<br>• Rosmarinic acid was identified as the major active metabolite in the leaf extract<br>• Network analysis revealed that metabolites affect key targets like FYN,	[144]

						PDGFRA, PDGFRB, PIK3R1, and ITGB3<br>• Molecular simulation confirmed the stable interaction of key metabolites, particularly rosmarinic acid, with the identified protein targets	
Cissus trifoliata (L.) L. (Leaves)	Ursolic acid, Phytol, Stearic acid, γ-Linolenic acid, Presqualene diphosphate	CASP3, PARP1, NR3C1, BAX, BCL2	Sesquiterpenoid and triterpenoid biosynthesis, Steroid biosynthesis, Diterpenoid biosynthesis	Human cancer cell lines (HepG2, Hep3B, HeLa, A549, PC3, MCF7)	None	• Hexane extract showed the highest cytotoxic activity against hepatocellular carcinoma cell lines (Hep3B and HepG2).<br>• The chloroform/methanol extract was the most selective against Hep3B cells.<br>• The most significant metabolic pathway identified was the sesquiterpenoid and triterpenoid biosynthesis pathway.<br>• Network pharmacology identified targets involved in apoptosis (CASP3, BAX, BCL2) as central to the extract's activity	[51]
Coleus amboinicus Lour. (Leaves)	16-Hydroxy-7α-acetoxyroyleanone (single isolated abietane diterpenoid)	MMP2, PPARG, BCL2	Pathways in cancer, PPAR signaling pathway, Neuroactive ligand-receptor interaction	Human prostate cancer cells (Du-145); CV-1 normal cells	None	• Single compound isolated from methanolic leaf extract; structure confirmed by NMR/FTIR/GC-MS<br>• Strong selective cytotoxicity against Du-145 (IC_{50} = 4.67 μg/mL, SI = 4.13), outperforming cisplatin<br>• Docking confirmed binding to MMP2, PPARG and BCL2 with key H-bond interactions	[81]
Gladiolus italicus Mill. (Aerial parts)	Hyperoside, Isoquercitrin, Delphinidin-3,5-diglucoside, Myricetin, Kaempferol-3-glucoside	AKT1, TP53, STAT3, TNF, MYC	PI3K-Akt signaling pathway, VEGF signaling pathway, AGE-RAGE signaling pathway, PD-L1/PD-1 checkpoint	Human prostate carcinoma cells (DU-145)	None	• Ethanolic extract was most cytotoxic against DU-145 (IC_{50} = 27.92 μg/mL), followed by ethyl acetate (37.14 μg/mL)<br>• Network pharmacology screening of 657 targets identified core hub genes related to PD-1, VEGFR2 and FGFR2 signaling<br>• MD simulations (100 ns) confirmed structural stability of phloretin–PD-1, kaempferol–VEGFR2 and isoquercitrin–	[85]

			pathway in cancer, HIF-1 signaling pathway			FGFR2 complexes	
Artemisia annua L. (Aerial parts)	3-(2-methylpropanoyl)-4-cadinene-3,11-diol, Artemisinin G, *O*-(2-propenal) coniferaldehyde, (2-glyceryl)-*O*-coniferaldehyde, Arteamisinin III	Prostate cancer, Breast cancer, Melanoma, Pancreatic cancer	Prostate cancer, Breast cancer, Melanoma, Pancreatic cancer	Human prostate (PC-3), breast (MDA-MB-231), pancreatic (PANC-1), and melanoma (A375) cancer cells	None	• The ethanolic extract of aerial parts showed potent, dose-dependent cytotoxicity against various cancer cell lines, with the highest efficacy against prostate (PC-3) cells<br>• Network pharmacology identified key phytoconstituents and their primary cancer-related targets, including NFKB1, MAP2K1, and AR<br>• Molecular docking and dynamic simulations confirmed stable interactions between the plant's compounds (specifically 3-(2-methylpropanoyl)-4-cadinene-3,11-diol and O-(2-glyceryl) coniferaldehyde) and key cancer-related proteins<br>The study elucidated a multi-component, multi-target, and multi-pathway mechanism for the anticancer action of A. annua	[98]
